# Non-linear frequency-doubling up-conversion in sulfide minerals enables deep-sea oxygenic photosynthesis

**DOI:** 10.1093/nsr/nwaf219

**Published:** 2025-05-28

**Authors:** Yan Li, Jiaqi Zhu, Qi Li, Hao Hong, Tao Li, Haoning Jia, Bingxu Hou, Houze Lu, Yanzhang Li, Jin Xie, Fuchen Wang, Huan Ye, Kaihui Liu, Anhuai Lu, Jindong Zhao

**Affiliations:** State Key Laboratory of Deep Earth and Mineral Exploration, School of Earth and Space Sciences, Peking University, Beijing 100871, China; Beijing Key Laboratory of Mineral Environmental Function, School of Earth and Space Sciences, Peking University, Beijing 100871, China; State Key Laboratory of Deep Earth and Mineral Exploration, School of Earth and Space Sciences, Peking University, Beijing 100871, China; Beijing Key Laboratory of Mineral Environmental Function, School of Earth and Space Sciences, Peking University, Beijing 100871, China; Key Laboratory of Algal Biology, Institute of Hydrobiology, Chinese Academy of Sciences, Wuhan 430072, China; State Key Laboratory for Mesoscopic Physics, Frontiers Science Centre for Nano-optoelectronics, School of Physics, Peking University, Beijing 100871, China; Key Laboratory of Algal Biology, Institute of Hydrobiology, Chinese Academy of Sciences, Wuhan 430072, China; State Key Laboratory of Deep Earth and Mineral Exploration, School of Earth and Space Sciences, Peking University, Beijing 100871, China; Beijing Key Laboratory of Mineral Environmental Function, School of Earth and Space Sciences, Peking University, Beijing 100871, China; State Key Laboratory of Deep Earth and Mineral Exploration, School of Earth and Space Sciences, Peking University, Beijing 100871, China; Beijing Key Laboratory of Mineral Environmental Function, School of Earth and Space Sciences, Peking University, Beijing 100871, China; State Key Laboratory of Deep Earth and Mineral Exploration, School of Earth and Space Sciences, Peking University, Beijing 100871, China; Beijing Key Laboratory of Mineral Environmental Function, School of Earth and Space Sciences, Peking University, Beijing 100871, China; State Key Laboratory of Deep Earth and Mineral Exploration, School of Earth and Space Sciences, Peking University, Beijing 100871, China; Beijing Key Laboratory of Mineral Environmental Function, School of Earth and Space Sciences, Peking University, Beijing 100871, China; State Key Laboratory for Mesoscopic Physics, Frontiers Science Centre for Nano-optoelectronics, School of Physics, Peking University, Beijing 100871, China; International Centre for Quantum Materials, Collaborative Innovation Centre of Quantum Matter, Peking University, Beijing 100871, China; Key Laboratory of Algal Biology, Institute of Hydrobiology, Chinese Academy of Sciences, Wuhan 430072, China; State Key Laboratory of Deep Earth and Mineral Exploration, School of Earth and Space Sciences, Peking University, Beijing 100871, China; Beijing Key Laboratory of Mineral Environmental Function, School of Earth and Space Sciences, Peking University, Beijing 100871, China; State Key Laboratory for Mesoscopic Physics, Frontiers Science Centre for Nano-optoelectronics, School of Physics, Peking University, Beijing 100871, China; International Centre for Quantum Materials, Collaborative Innovation Centre of Quantum Matter, Peking University, Beijing 100871, China; State Key Laboratory of Deep Earth and Mineral Exploration, School of Earth and Space Sciences, Peking University, Beijing 100871, China; Beijing Key Laboratory of Mineral Environmental Function, School of Earth and Space Sciences, Peking University, Beijing 100871, China; Key Laboratory of Algal Biology, Institute of Hydrobiology, Chinese Academy of Sciences, Wuhan 430072, China; State Key Laboratory of Gene Function and Modulation Research, School of Life Sciences, Peking University, Beijing 100871, China

**Keywords:** second harmonic generation, sulfide mineral, chalcopyrite, deep-sea hydrothermal system, oxygenic photosynthesis, cyanobacteria

## Abstract

Visible light emission exceeding purely thermal radiation has been imaged at deep-sea hydrothermal vents, yet the underlying mechanisms remain unexplained. Here, we show that visible light can be produced from geothermal infrared radiation via nonlinear frequency-doubling up-conversion in sulfide minerals that are abundant in hydrothermal vents. Chalcopyrite exhibits significant second harmonic generation, which is further amplified under high pressure, yielding a 400–700 nm photon flux three orders of magnitude greater than blackbody emission. When exposed to 1064 nm of irradiation, chalcopyrite induces fluorescence responses in the cyanobacterium *Synechococcus* sp. PCC 7002 at 656 and 685 nm, suggesting that the up-converted 532 nm light is absorbed by phycobilisomes and transferred to photosystem II. Metagenomic analysis reveals a strong correlation between cyanobacteria and high-temperature, chalcopyrite-rich vents. Similar up-conversion processes have also been observed in other sulfide minerals, emitting wavelengths covering the entire visible spectrum. These findings unveil a novel mineral-mediated photonic mechanism that generates biologically relevant visible light at hydrothermal vents, which can be harnessed by oxygenic photosynthetic cyanobacteria in Earth's deep biosphere and possibly beyond.

## INTRODUCTION

Photosynthesis has fueled primary production on Earth for an estimated 3.3–3.4 billion years, continuously shaping the planet surface [1,2]. Surprisingly, photosynthetic prokaryotes like cyanobacteria have also been discovered in deep sulfide deposits associated with both modern submarine hydrothermal vents [3,4] and ancient hydrothermal activity zones [5]. These findings challenge the conventional understanding that oxygenic photosynthesis is impossible in deep hydrothermal systems that are extremely deficient in visible photon fluxes. Despite recent advances in the tolerance of photosynthetic microorganisms to hydrothermal extreme environmental factors, such as high temperatures, low light flux and exposure to metal sulfides [6–8], the long-term survival of photosynthetic cyanobacteria in such deep-earth environments remains a mystery.

The high-temperature upwelling fluids (up to 400°C) in hydrothermal vents not only bring abundant reductants (e.g. H_2_S, Fe^2+^) [9], but also, importantly, emit geothermal radiation [10–14]. Some anaerobic photosynthetic bacteria have been shown to survive through harvesting low-energy photons from the weak red and near-infrared spectrum of this geothermal radiation [7,8]. For instance, a green sulfur bacterium isolated from a hydrothermal vent in the East Pacific Rise area employs bacteriochlorophyll *c* (BChl *c*) to absorb and utilize red/infrared light [8]. However, the survival of oxygenic photosynthetic cyanobacteria in hydrothermal vents [3,4] poses greater challenges due to the requirement for higher-energy photons. According to blackbody radiation principles, photon emission in the visible-light range from hydrothermal vents is significantly limited [11–14].

Previous *in situ* underwater spectral cameras have recorded a remarkable photon flux of visible light (400–600 nm) around hydrothermal vents, reaching 10^4^ photons·cm^−2^·s^−1^·sr^−1^, which exceeds the upper limit of thermal blackbody radiation (10^3^ photons·cm^−2^·s^−1^·sr^−1^) [13,14]. The actual photon fluxes may be even higher, given the signal attenuation caused by light scattering over measurement distances [12] and data-processing losses during image flat-fielding correction and noise elimination [13,14]. The origin of this excess visible light remains unresolved. Proposed hypotheses, including crystalloluminescence [15] and triboluminescence [16], are typically associated with the presence of sugars or copper sulfate, while chemiluminescence [17] can only be efficient when induced by the rapid oxidation of sulfides in aerobic environments. Although vapor bubble luminescence [18] can rapidly generate intense flashes, its transient and intermittent nature precludes the sustained and stable photon flux necessary for photosynthesis.

Here, we present experimental evidence that sulfide minerals in hydrothermal vents exhibit an infrared-to-visible photon up-conversion property, which is greatly enhanced by pressure and can provide sustainable visible photons for cyanobacterial photosynthesis in deep-sea hydrothermal vents. This unique mineralogical mechanism of visible light generation provides insights into cyanobacterial growth in deep hydrothermal systems and has implications for the origin, evolution and even the current occurrence of oxygenic photosynthesis on Earth and other water worlds in the solar system.

## RESULTS AND DISCUSSION

### Characterization of second harmonic generation in natural sulfides

**Figure 1.**
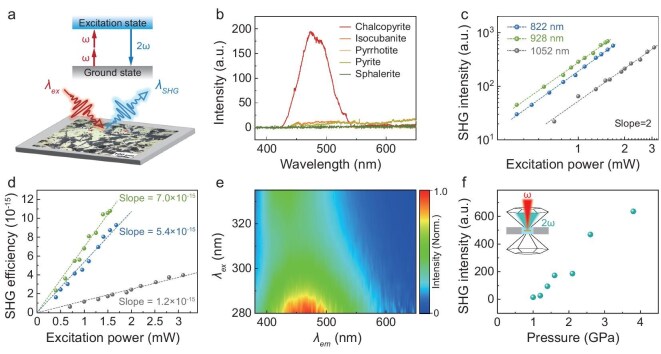
Optical spectrum analysis of chalcopyrite from deep-sea hydrothermal vents. (a) Schematic diagram of frequency-doubling measurement on the deep-sea hydrothermal sulfides. Two photons at frequency *ω* interact with the sulfide sample and generate a new photon with a doubled frequency (2*ω*) and half the wavelength of the excitation light. (b) Emission spectra of sulfide minerals under a supercontinuum laser (800–1500 nm). Compared with the other four sulfides, the spectrum of chalcopyrite shows an obvious peak at ∼471 nm. (c) Excitation-power-dependent second harmonic generation (SHG) intensity in chalcopyrite. Under different excitation wavelengths, all three curves show the expected quadratic law. (d) Excitation power-dependent frequency-doubling conversion efficiency in chalcopyrite. The conversion efficiency increases linearly with the excitation light power, arriving at over ∼10^−14^ at 1.5 mW with 928 nm of excitation. (e) 2D photoluminescence spectrum of chalcopyrite showing a peak at ∼471 nm, consistent with that from the SHG spectrum in (c), indicating a special excitation state at ∼2.63 eV. (f) Emission spectra of chalcopyrite measured in a diamond anvil cell (DAC), showing increasing SHG intensity with hydrostatic pressure.

Spectroscopic measurements were performed on sulfide minerals from high-temperature hydrothermal vents to explore their luminescent characteristics (Fig. 1). The sulfide samples were collected from the Longqi hydrothermal vent field in the Southwest Indian Ridge. Their phases, as identified by using X-ray diffraction (Fig. S1), mainly comprised chalcopyrite (CuFeS_2_), sphalerite (ZnS), pyrite (FeS_2_) and pyrrhotite (Fe_1__–_*_x_*S, *x* < 1) with mass percentages of 54%, 26%, 15% and 5%, respectively. Considering that hydrothermal vents mainly emit thermal radiation within the infrared spectrum, we used wide-band infrared light (800–1500 nm) to excite the sulfide minerals (Fig. 1b). The emission spectra revealed that chalcopyrite exhibited detectable light emissions in the range of 420–540 nm, with an emission peak at ∼471 nm (Fig. 1b). The maximum emission peak remained unchanged under different excitation light powers (Fig. S2). In contrast, the other four sulfide minerals exhibited no discernible visible light emissions. The spectral overlap between chalcopyrite second harmonic generation (SHG) emission and the excess photon flux (400–550 nm) observed at hydrothermal vents suggests that chalcopyrite is a desired candidate for efficiently up-converting infrared to visible light in deep-sea environments.

To study the infrared-to-visible photon up-conversion mechanism of chalcopyrite, we measured the emission spectra under three monochromatic lights (822, 928 and 1052 nm) and at different excitation powers (0.45–3.20 mW). The emission peak was exactly centered at half the excitation wavelength (411, 464 and 526 nm) and remained unchanged with increasing excitation power (Fig. S3). The double-frequency emission intensity of chalcopyrite exhibited a quadratic correlation with the excitation power (Fig. S4), as supported by fitting slope values of 2 in the logarithmic coordinate view (Fig. 1c). The frequency-doubling conversion and the quadratic relationship indicated that the up-conversion mechanism was attributed to the SHG effect [19]. In brief, two photons with frequency *ω* excited chalcopyrite from the ground state to an excited state, resulting in the emission of a new photon with twice the energy of the initial photon (i.e. double the frequency and half the wavelength, as shown in Fig. 1a). Based on the emission spectra under three monochromatic lights, we calculated the energy-conversion efficiency (*η*) of chalcopyrite at varying excitation powers (*P*_ex_) and derived linear relationships between *η* and *P*_ex_ (Fig. 1d). As *P*_ex_ gradually increased from 0.4 to 1.5 mW, *η* increased from 10^−15^ to 10^−14^. Notably, the SHG efficiency of chalcopyrite is strongly dependent on the excitation wavelength, as illustrated by diverging linear fitting slopes (Fig. 1d).

To further explore the excitation-wavelength-dependent SHG effect in chalcopyrite, we measured the photoluminescence (Fig. 1e) spectra of chalcopyrite to probe the electronic transitions between the specific excitation and ground states. The 2D photoluminescence spectrum showed that, under ultraviolet light excitation (*λ*_ex_ = 280–340 nm), chalcopyrite exhibited an intense and fixed emission in the range of 420–500 nm (*λ*_em_), peaking at ∼471 nm (Fig. 1e). This peak position was in accordance with the SHG emission peak position presented in Fig. 1c, indicating the existence of an energy gap at ∼2.63 eV (corresponding to a photon wavelength of 471 nm) between the excited state and the ground state of chalcopyrite. Upon irradiation at 928 nm, approximately double the wavelength of 471 nm, chalcopyrite underwent resonant excitation that maximized the up-converting efficiency at the energy gap, thereby producing a strong SHG emission at 471 nm. This up-conversion mechanism is distinct from fluorescence or down-conversion luminescence mechanisms (e.g. rare-earth element luminescence) previously found in minerals [20,21]. It enables chalcopyrite to constantly convert geothermal infrared radiation into visible light when exposed to a thermal radiation field.

### Pressure-enhanced SHG effect in chalcopyrite

Notably, the deep-sea environment is characterized by high-pressure conditions. To understand the relationship between the SHG efficiency of chalcopyrite and the hydrostatic pressure, pressure experimental simulation was performed in a diamond anvil cell (DAC). The results indicated a significant increase in the up-conversion efficiency of chalcopyrite from infrared to visible light under high hydrostatic pressure, with the maximum efficiency reaching 10^−7^, which is 66 times higher than its initial value (Fig. 1f). The enhanced SHG effect under pressure can be explained by Miller's rule, in which the increased material density and refractive index under pressure contribute to an augmented non-linear susceptibility [22]. Additionally, the cation displacement within the mineral lattice and the evolved band structure with increasing pressure further contribute to the enhancement of SHG (Fig. S5) [23]. This implies that the flux of visible photons converted by chalcopyrite could be substantially enhanced under real deep high-pressure environments, such as deep hydrothermal vents.

According to the blackbody curve, the photon flux of hydrothermal vents at 400°C is in the order of ∼10^12^ photons·cm^−2^·s^−1^·sr^−1^ in the range of 800–1100 nm. Considering the pressure effect, the photon fluxes in the range of 400–550 nm generated by the SHG effect of chalcopyrite (with maximum efficiency of ≤10^−7^) could reach 6.6 × 10^6^ photons·cm^−2^·s^−1^·sr^−1^, which is three orders of magnitude greater than the blackbody radiation [11–14]. Previous research on the self-focusing effect induced by thermal gradients suggests that localized photon flux could be further enhanced through mineral micropores in the steep temperature gradient field of hydrothermal vents [24]. Therefore, the pressure-enhanced SHG effect in chalcopyrite could significantly contribute to the anomalously high blue–green visible photon flux observed at hydrothermal vents.

### Fluorescence response of cyanobacteria to infrared-irradiated chalcopyrite

**Figure 2.**
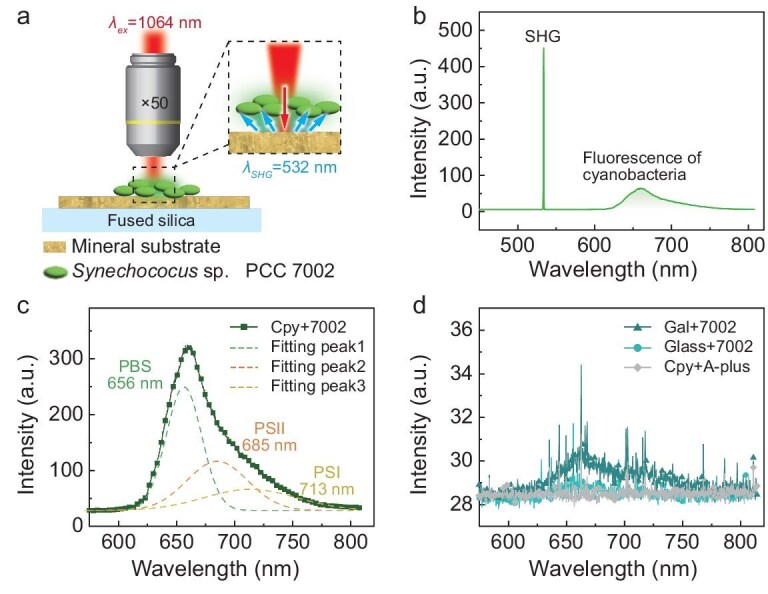
Fluorescence response of cyanobacteria to infrared-irradiated sulfide minerals. (a) Schematic diagram of the fluorescence experiments. The wavelength of excitation laser (*λ*_ex_) and the wavelength of SHG (*λ*_ex_) is 1064 and 532 nm, respectively. The magnification of objective lens is denoted as ×50. (b) Emission spectrum of *Synechococcus* sp. PCC 7002 (7002) upon a chalcopyrite substrate irradiated by 1064 nm infrared light. (c) Fluorescence spectra of 7002 recorded on chalcopyrite (Cpy, curve with square). The fluorescence emission from 7002 on Cpy can be well fitted with three component peaks at 656, 685 and 713 nm, indicative of the up-converted light is absorbed by phycobilisome (PBS) and the excitation energy is transferred to photosystem II (PSII) and photosystem I (PSI). (d) Fluorescence spectra of 7002 recorded on glass (Glass, curve with circle) and galena (Gal, curve with triangle). No detectable fluorescence was observed when substituting Cpy with a glass or Gal substrate, or replacing 7002 with a sterile culture medium (A-plus, curve with diamond).

Cyanobacterial pigments efficiently absorb visible light within the range of 400–710 nm [25]. While the majority of excitation energy transferred to photosystem II (PSII) from phycobilisomes (PBS) is used to drive electron transport for oxygen evolution, it can also produce fluorescence through a relaxation process [26]. To investigate whether infrared light can be used to excite PSII, the cyanobacterium *Synechococcus* sp. PCC 7002 (*Synechococcus* 7002) was placed on a chalcopyrite substrate and *in situ* measurement of its fluorescence response to infrared light was performed (Fig. 2a).

Upon excitation by using a 1064 nm monochromatic light (*P*_ex_ = 13 mW), we observed a sharp peak at 532 nm and a broad peak ranging from 610 to 810 nm in the emission spectrum (Fig. 2b). When chalcopyrite was replaced by a glass substrate or galena (PbS, with similar reflectivity and thermal conductivity to chalcopyrite), no significant fluorescence emission was recorded (Fig. 2d), contrasting sharply with the chalcopyrite substrate (Fig. 2c). If *Synechococcus* 7002 was replaced by a sterile culture medium (A-plus), the broad emission peak was not detected (Fig. 2d). Thus, the 532 nm peak corresponds to the SHG emission in chalcopyrite, while the broad emission peak comes from the cyanobacterial cells.

The cyanobacterial fluorescence spectrum can be fitted with three component peaks at 656, 685 and 713 nm (Fig. 2c). The peaks at 656 and 685 nm indicate that the up-converted light by chalcopyrite is successfully absorbed by the PBS and excitation energy is transferred to PSII [27,28]. Despite the partial overlap between the two peaks within the range of 680–800 nm, the component peak at 713 nm aligns with the characteristic fluorescence emission wavelength of photosystem I (PSI) in *Synechococcus* 7002 [29,30]. This observation suggests that excitation energy can be transferred to PSI, consistently with recent studies on interactions between rod-shaped phycobilisomes and PSI [31,32]. Distinct from Chl *d/f*-dominated infrared absorption supporting PSI centric cyclic electron transport (P700 → P700*), mineral-up-converted visible light can directly provide higher-energy photons to drive PSII charge separation and electron transport for oxygen-evolving reactions [33].

### Close correlation of deep cyanobacteria with high-temperature vents revealed by metagenomics

**Figure 3.**
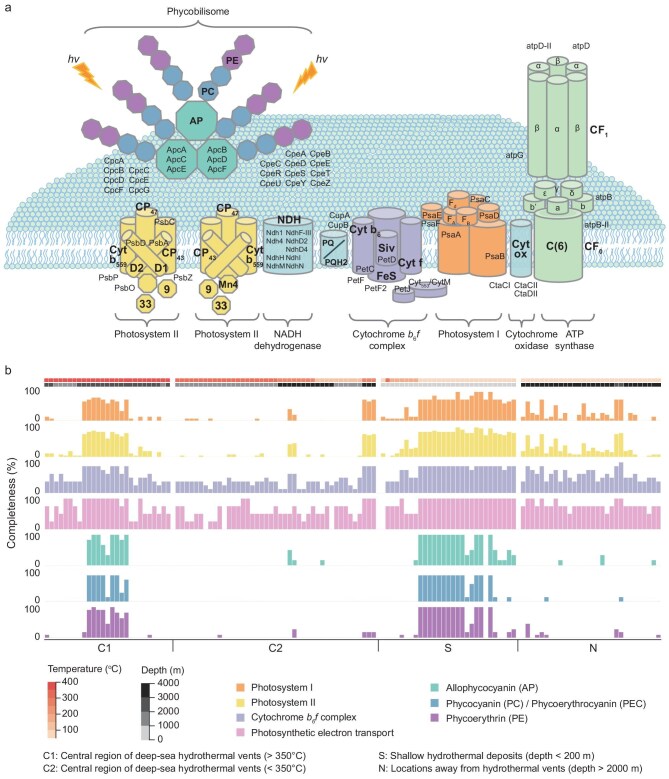
Metagenomic analysis of photosynthesis-related genes in marine systems. (a) Diagram illustrating photosynthetic electron transfer and functional genes associated with the phototrophic apparatus, including Photosystem I, Photosystem II, cytochrome *b_6_f* complex, photosynthetic electron transport, allophycocyanin (AP), phycocyanin (PC)/phycoerythrocyanin (PEC) and phycoerythrin (PE). (b) Completeness of functional genes related to the phototrophic apparatus of datasets across four marine environments. Metagenomic datasets are classified into: 27 from central region of deep-sea hydrothermal vents (C1, >350°C), 43 from central region of deep-sea hydrothermal vents (C2, <350°C), 30 from shallow hydrothermal deposits (S) and 42 from deep-sea locations distant from hydrothermal vents (N).

As no successful isolation and culture of oxygenic photosynthetic microorganisms from deep-sea hydrothermal systems have been reported, assessing the completeness of photosynthetic functional genes appears to be an ingenious suboptimal approach for investigating the potential presence of oxygenic photosynthetic bacteria in the deep sea. A total of 142 sets of metagenomic data were collected from 19 global hydrothermal vents and 24 deep-sea locations that were distant from hydrothermal vents to analyse photosynthesis-related genes. These datasets were classified into four types based on their mineral components, geographical locations and temperature ranges (Fig. 3b): hydrothermal vents with a central temperature of >350°C are denoted as Type C1, enriched in Cu- and Fe-sulfide minerals such as chalcopyrite, isocubanite and pyrrhotite; hydrothermal vents with a central temperature of <350°C are denoted as Type C2, generally enriched in Pb- and Zn-sulfide minerals such as sphalerite and galena; shallow-water hydrothermal deposits in the epipelagic zone are denoted as Type S; and deep-sea locations that are distant from hydrothermal vents are denoted as Type N [34,35]. The photosynthesis genes analysed in this metagenomic comparison include those encoding proteins of PSI, PSII, cytochrome *b_6_f* complex (Cyt*b_6_f*), photosynthetic electron transport, F-type ATPase and PBS, which function as light-harvesting antennas (Fig. 3a and Fig. S6).

The completeness of genes encoding each phototrophic apparatus in all metagenomic datasets is presented in Fig. 3b, including 27 datasets from Type C1, 43 datasets from Type C2, 30 datasets from Type S and 30 randomly selected datasets from Type N (out of 42 available). A comprehensive metagenomic analysis of photosynthesis-related genes in all datasets is illustrated in Fig. S6. Notably, 10 out of the 27 datasets from Type C1 (C1_9–18) exhibit a high degree of completeness in genes associated with phototrophic apparatuses. In these datasets, the genes encoding PSI, PSII, Cyt*b_6_f* and components of photosynthetic electron transport are nearly complete, with the average completeness of PBS genes approaching 80%. Specifically, these datasets contain a rich array of photosynthetic genes encoding the proteins involved in oxygenic photosynthesis, such as the *psa* and *psb* genes encoding PSI and PSII subunits, respectively. The majority of the *psa* genes, including *psaA, psaB, psaC, psaD, psaE, psaF, psaI, psaJ, psaK, psaL* and *psaM*, are found in these datasets. Moreover, the Type C1 datasets that have *psa* genes simultaneously possess high-abundance *psb* genes encoding PSII subunits, such as PSII reaction center proteins D1, D2, Cytb559, CP47 and CP43 [36]. Among other photosynthetic electron-transfer proteins, a complete set of Cyt*b_6_f* complex genes are found in these datasets from Type C1. The presence of Cyt*b_6_f* small subunit genes and *petH* (plant-type ferredoxin: reduced nicotinamide adenine dinucleotide phosphate (NADPH) oxidoreductase, FNR) is particularly interesting, as they are present only in the oxygenic photosynthetic electron transfer chain.

The datasets from Type C1 with the photosynthetic genes above also possessed a set of *apc, cpc* and *cpe* genes that encode allophycocyanin, phycocyanin and phycoerythrin of PBS, respectively. Notably, cyanobacteria can dynamically adjust PBS components to optimize the selective utilization of specific chromophore types, thereby maximizing the absorbance of the predominant wavelengths in the ambient light spectrum [37]. Geothermal radiation emitted by hydrothermal vents is dominated by infrared light and an excess photon flux in the range of 400–550 nm. While far-red-light photoacclimation (FaRLiP) could theoretically facilitate photosynthesis in such environments, metagenomic analysis revealed no FaRLiP-associated genes (*apcD2, apcD3* and *apcD5*) [38–40] in Type C1 samples (Fig. S6), excluding the potential distraction of FaRLiP. This finding suggests that hydrothermal photosynthesis relies on visible light rather than far-red/infrared light.

Metagenomic assembly demonstrated the consistent detection of key cyanobacterial photosynthetic genes (*psaABCD, psbABCD, apcABC, cpcABC* and *cpeABC*) in vent-associated samples (C1_10–18), while these genes were virtually absent in randomly selected control samples (Fig. S7). These findings are in accordance with those mentioned previously in this section, thereby collectively reinforcing the presence of cyanobacterial photosynthetic genes at high-temperature mineral-containing hydrothermal vents.

To distinguish between *in situ* activity and passively transported sources, we further traced the corresponding species for common photosynthetic genes, including *apcAB, cpcAB, psaABC* and *psbABC* (Data S2). Notably, *Prochlorococcus psb* genes, which are expected in settling biomass due to their marine abundance [41], are absent in the samples with complete oxygenic photosynthetic genes (*psa, psb, apc, cpc*) and electron-transport-chain components (C1_10/11/12/13/15/16/17). This discrepancy challenges passive settling hypotheses, supporting endogenous cyanobacterial DNA at vents.

Based on the *in situ* microbial information in datasets from Type C1, we can decode endemic ecological advantages for sustaining complete photosynthetic genes in high-temperature mineral-containing vents, by conducting comparisons with datasets from diverse geographical locations, including Type C2, Type S and Type N. The datasets from Type C2, featuring a lower temperature (<350°C) and different mineral components from Type C1, exhibit a general absence of PSI, PSII and Cyt*b_6_f* genes, except for 5 of 43 datasets (SRR14000033, SRR14000034, SRR7168047, SRR7168048 and SRR7168049). These five datasets, which were obtained from deep-sea locations (>3000 m) with temperatures of ∼290°C, do not contain a complete set of genes encoding the proteins of PBS or some photosynthetic electron-transfer chain (*psbX, psbY, psbZ*). This suggests that hydrothermal environments with higher central temperatures appear to be more favorable for preserving genes encoding light-harvest antenna proteins.

Our analysis further extended to Type S datasets, which contain a relatively complete set of photosynthetic genes, as previously reported [42,43]. By contrast, nearly all genes encoding PBS were absent in Type N datasets, with >70% of genes encoding PSI and PSII remaining incomplete. These findings further reveal the crucial role of hydrothermal-vent-derived geothermal radiation in maintaining complete photosynthetic functional genes in phototrophic microorganisms. Particularly, the cyanobacteria founded in deep subsurface rock have completely lost the fluorescence response of PSII [5]. This is consistent with the results of our metagenomic analysis of the two samples (T1_1/2): half of the genes encoding PSII and almost all the genes encoding phycocyanin, phycoerythrocyanin and phycoerythrin of PBS are missing. However, our findings with relatively complete genes encoding oxygenic photosynthesis and light-harvesting antenna proteins imply a more active phototrophic mode at hydrothermal vents.

Notably, certain metabolic strategies may bridge these ecological extremes. For example, some *Synechocystis* species (e.g. PCC 6803) can engage in light-activated heterotrophic growth (LAHG) [44–46], while metagenomic evidence from the active Jabberwocky vent reveals the co-occurrence of complete Calvin–Benson–Bassham and reductive tricarboxylic acid cycles [4], suggesting metabolic flexibility in these environments. These findings collectively point to hydrothermal vents as unique niches in which geothermal radiation sustains phototrophic potential, while fostering diverse energy-capture strategies.

Besides the datasets mentioned above, 19 additional sets of metagenomic data from the oxidation zone of hydrothermal minerals (denoted as O, enriched in Ba- and Ca-sulfate minerals) and the thermophilic microbial community after enrichment culture (denoted as T2) were analysed (Fig. S6). The photosynthetic genes were also observed in datasets from T2, although some *psa* and *psb* genes were not detected. Datasets from Type O exhibited a lower abundance of photosynthetic genes overall. While some datasets contained relatively complete sets of photosynthetic genes, they were almost entirely missing the *apc, cpc* and *cpe* genes that encode phycobiliproteins (Fig. S6).

As nearly all datasets contained an intact set of *atp* genes encoding F-type ATPase, reads mapped to these genes from various species were utilized to analyse community composition and diversity (Fig. S8). Cyanobacteria were observed in the Type C1 datasets, and the microbial composition of Type C1 showed similarities to that of Type S datasets from the shallow euphotic zone. Comparative analysis of Types C1, C2 and O suggests that vent-specific metal sulfide minerals may create unique ecological niches favoring oxygenic phototrophs in aphotic depths. While prior metagenomic studies have reported sporadic occurrences of oxygenic phototrophs in hydrothermal systems [3–5], our work provides the first comprehensive genomic evidence for the preservation of complete photosynthetic pathways in high-temperature-vent environments.

### Extensive visible emissions in hydrothermal sulfide minerals

We have demonstrated that natural chalcopyrite exhibited a strong SHG effect in the near-infrared region (Fig. 1), with a non-linear frequency-doubling up-conversion efficiency that was comparable to those of artificial chalcopyrite-structured materials, such as AgGaS_2_ [47], AgGaSe_2_ [47] and ZnGeP_2_ [48]. Theoretically, sulfide minerals with non-centrosymmetric crystal structures, high non-linear optical coefficients and suitable electronic band structures [49,50] may exhibit strong non-linear frequency-doubling up-conversion effects. Given the diversity of hydrothermal sulfide minerals, we investigated the potential of 12 additional sulfide minerals (Figs S8 and S10) to up-convert near-infrared light to visible light.

**Figure 4.**
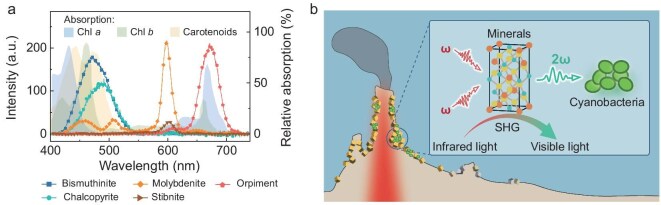
Extensive visible emissions in hydrothermal sulfide minerals. (a) SHG emission spectra of five sulfide minerals overlapping with the absorption spectra of photosynthetic pigments (chlorophyll *a*: blue area, chlorophyll *b*: green area, carotenoids: yellow area). (b) Schematic illustrating the mineralogical mechanism that converts infrared light into visible light via SHG, thereby supporting photosynthetic microorganisms in the deep sea.

Upon being triggered by light of 800–1500 nm, molybdenite (MoS_2_), stibnite (Sb_2_S_3_), bismuthinite (Bi_2_S_3_) and orpiment (As_2_S_3_) emitted light within the range of 420–725 nm (Fig. 4a). It indicates that, besides the superior up-conversion efficiency of chalcopyrite in the blue–green band (Fig. S11), other sulfide minerals could also contribute visible light sources in deep hydrothermal fields through broadening the up-conversion wavelength range. Collectively, their emission wavelengths would cover the full range of the visible-light spectrum from the blue–green band of 400–500 nm to the red band of 600–700 nm. The photon flux generated by SHG from various minerals could provide energy to support oxygenic photosynthesis in deep-sea environments (Fig. 4b) through diverse photosynthetic pigments such as chlorophyll *a*, chlorophyll *b* and carotenoids [51,52]. For example, the SHG emission spectra of chalcopyrite and bismuthinite offer visible light within the range of 400–550 nm, matching the absorption peak of chlorophyll *a* at ∼430 nm and that of phycourobilin at ∼495 nm [53]. Molybdenite and stibnite with SHG emission peaks at 570–620 nm can provide visible light for phycoerythrocyanin with an absorption peak at ∼575 nm [54]. These multi-sourced visible lights may also benefit other phototrophic bacteria in the deep sea that absorb light in the range of 450–700 nm.

### Geological implications

Chalcopyrite, as one of the earliest and major sulfide minerals in high-temperature hydrothermal environments, exhibits a strong SHG effect through non-linear optical processes that convert two infrared photons into a single visible photon with doubled frequency. This frequency-doubling up-conversion phenomenon is predominantly observed in minerals with broken inversion symmetry, such as the hydrothermal sulfide minerals in this study. With a pressure-enhanced effect, the chalcopyrite-mediated photon conversion at a 400°C vent can generate visible emission exceeding blackbody radiation values by three orders of magnitude, aligning with the excess blue–green light observed at hydrothermal vents. Additionally, previous studies have demonstrated that the metal sulfides at hydrothermal vents can exist as aggregates of nanoparticles (individual as small as 4 nm) [55,56]. The resonance enhancement resulting from the nano-microcavity structure on the mineral surface (Fig. S12) and the self-focus effect induced by the steep thermal gradient can further improve the up-conversion emission by three orders of magnitude [19,20,24,57]. Based on these findings, we propose that deep hydrothermal sulfide minerals such as chalcopyrite provide a significant flux of visible light around the vents by up-converting the abundant geothermal infrared light (Fig. 4b).

The fluorescence experimental evidence from *Synechococcus* 7002 demonstrates efficient energy transfer from mineral-converted visible photons to PBS, subsequently activating both PSII and PSI reaction centers (Fig. 3a). This offers a strong physical basis for understanding the discovery that most oxygenic photosynthetic genes—especially those encoding light-harvesting antenna proteins—are present in deep high-temperature hydrothermal vents. The observed correlation between cyanobacterial DNA and regions with high temperature (>350°C), high hydrostatic pressure and the occurrence of sulfide minerals suggests endemic ecological niches supporting deep-sea photosynthesis. These findings not only open up new pathways for exploring how deep-sea oxygenic photosynthesis functions, but also probe potential geochemical drivers for the evolution of oxygenic photosynthesis.

Cyanobacteria played a pivotal role in the Great Oxidation Event (GOE) at ∼2.4 Ga and their marine expansion is regarded as a key driver for atmospheric O_2_ rise and carbon burial [58,59]. Interestingly, the GOE coincided with the break-up of the supercontinent that spurred global magmatic processes and extensive deposition of hydrothermal minerals [60,61]. This deep temporal correlation suggests a possible causal relationship between hydrothermal activities and thriving cyanobacteria. Besides, given the geological hydrothermal activities prior to the GOE and their continuous eruption [61], the mechanism described in our report could also plausibly have contributed to the so-called whiffs of oxygen on early Earth before the GOE [62]. Specifically, with early Earth's geothermal heat flux that was two to three times higher than the modern value [63], the visible light flux produced by this up-conversion mechanism in sulfide minerals would have been higher. The abundant microcavities in the actual hydrothermal chimney (Fig. S11) can not only enhance the SHG effect [57], but also provide shelters against intense ultraviolet radiation [64] or the strong impact of hot fluids or toxic vapors for early oxygenic photosynthetic microorganisms.

Although previous studies have proposed alternative habitat preferences for the origin of early cyanobacteria (e.g. soil/freshwater, low-salinity environments) [65,66], our findings demonstrate that the mineral up-conversion mechanism could have provided a crucial energy source for the parallel evolution, adaptation and colonization of early oxygenic photosynthetic microorganisms in hydrothermal environments. Our results support the hypothesis that early cyanobacteria might have utilized excess light emission at hydrothermal vents to occupy ecological niches in the deep sea, where they thrived and potentially evolved sophisticated photosynthetic proteins within these deep hydrothermal environments.

While our findings show a blue–green light source, continuous light stimulation and selective pressure for photosynthesis at hydrothermal vents, several limitations warrant cautious interpretation. The *in situ* observed photon flux (>10^4^ photons·cm^−2^·s^−1^·sr^−1^) and mineral up-conversion emission evaluated in this work (∼10^7^ photons·cm^−2^·s^−1^·sr^−1^) exhibit measurable discrepancies from the photon requirement for cyanobacterial laboratory growth (∼10^13^ photons·cm^−2^·s^−1^·sr^−1^). However, emerging evidence suggests that natural phototrophs may operate at lower photon fluxes than laboratory models. For example, Hoppe *et al.* [67] demonstrated that oxygenic photosynthesis can persist at photon fluxes as low as 0.04 ± 0.02 µmol photons·m^−2^·s^−1^ (∼10^11^ photons·cm^−2^·s^−1^·sr^−1^), implying the adaptations of natural phototrophs to extreme low-light conditions that remain poorly understood [67]. Consequently, while the lack of *in situ* isolation/cultivation evidence and precise spectral emission measurements in deep-sea environments restricts our capacity to fully resolve this gap, our study establishes the intrinsic photon-generating capacity of minerals as a novel mechanism warranting further interdisciplinary investigation.

In summary, our findings on mineral up-conversion potentially contribute to the essential light source for the evolution and operating of oxygenic photosynthesis in Earth's deep hydrothermal vents. This also raises the possibility of discovering new lineages of photosynthetic microorganisms and even an ecosystem beneath the icy surfaces of other water worlds beyond Earth, such as Europa [68] and Enceladus [69], which potentially host ice-covered oceans supported by live hydrothermal vents. To facilitate future astronomical and astrobiological explorations, we propose establishing a coupled mapping framework of spectral signals between mineral SHG and photosynthetic pigments, providing a novel mineralogical approach for detecting potential photosynthetic life beyond Earth.

## METHODS

### Optical characterization of minerals

The SHG effects of natural black chimney samples and other sulfide minerals were measured by using an incident light source generated from the supercontinuum white light system (WhiteLaser SC400, Fianium) with a basic pulse width of 6 ps and a fundamental frequency of 40 MHz. The conversion-efficiency comparison experiment of chalcopyrite (CuFeS_2_), bismuthinite (Bi_2_S_3_), stibnite (Sb_2_S_3_), orpiment (As_2_S_3_) and molybdenite (MoS_2_) was conducted by using a titanium–sapphire pumped laser (Mira-HP, Coherent) as the incident light, with a basic pulse width of ∼130 fs and a fundamental frequency of 76 MHz. The SHG spectroscopies were measured by using a home-made set-up with reflective geometry at room temperature (Fig. S13). The spot size of the pumped laser was ∼10 μm^2^ under the focus of a 20× objective lens (Nikon) and ∼4 μm^2^ under the focus of a 50× objective lens (Nikon). The generated SHG signal was collected by using the same objective. After filtering out the excitation laser, the SHG signal was recorded by using a spectrometer (SP2500, Princeton Instruments) equipped with a nitrogen-cooled silicon charge coupled device (CCD) camera (PyLoN 400 BRX, Princeton Instruments). A monochromatic light signal was obtained by placing a bandpass filter according to the desired wavelength. The quantum-harvesting efficiency of the spectrometer and CCD was ∼55%. The fiber coupling efficiency was 60%. The loss efficiency of the beam-splitting prism was 50%. Based on the above loss, the output SHG power and photon emission power were calculated. The excitation power was measured by using an optical power meter (PM20CH, Thorlabs). The energy-conversion efficiency equals the ratio of the original outgoing light power to the excitation power.

The 2D photoluminescence spectrum was measured by using a spectrometer (FL3-TCSPC, Horiba Jobin Yvon). The excitation light source was monitored at 5-nm intervals from 280 to 340 nm and the photoluminescence signal was collected over a range of 350–650 nm. Ultraviolet–visible diffuse reflectance spectroscopy was measured by using a spectrometer equipped with a diffuse integrating sphere attachment (UV-3600 Plus, Shimadzu). BaSO_4_ was used as a reference. The slit width of the incident light was 2.00 nm.

### Cyanobacterial strain and room-temperature fluorescence spectra

The cyanobacterial strain *Synechococcus* sp. PCC 7002 was cultured in media A supplemented with 0.01 M NaNO_3_ (designated as A-plus media), bubbled with 1% CO_2_. The growth temperature was 34°C and the light intensity was 250 μmol m^−2^ s^−1^, provided by cool-white fluorescent lights. The fluorescence spectrum of cyanobacteria upon different substrates (including chalcopyrite, glass and galena) was measured by using a Witec system (Alpha-700R) at room temperature, with an excitation wavelength of 1064 nm (pulse duration: 10 ps). The cyanobacteria were subjected to 15-minute dark adaptation before the fluorescence experiments were conducted.

### DAC experiments

High-pressure SHG optical experiments were performed with the symmetric DAC. Ultralow fluorescence diamonds with a 400-μm culet was used. The sample chamber was defined by a hole with a diameter of ∼200 μm in a previously indented steel gasket. KBr and silicone oil were used as the pressure-transmitting medium. The pressure of the sample environment was determined by the fluorescence emission of a ruby sphere loaded near the center of the sample.

### Metagenomic analysis

We downloaded 163 published metagenomics datasets (Data S1) to analyse the existence of photosynthesis-related genes. The latitude and longitude information of the datasets is included in Data S1. All photosynthesis-related genes were obtained from the KEGG Orthology database [70] to construct a photosynthetic gene database. The species information corresponding to the key photosynthetic genes is listed in Data S2. Metagenomic assemblies were generated by using the MEGAHIT (v1.2.9) with default parameters [71]. The metagenomic reads from each dataset were mapped to this database by using the DIAMOND BLASTx (v2.0.15.153) [72] to find the best hits. When multiple genes shared an alignment, the numbers of read hits were averaged. Metagenomic coverage values of photosynthesis-related genes were normalized by using the number of metagenomic reads in each sample. Subsequently, a Perl script was used to calculate the maximum coverage for each gene and filter out results with coverage of <80%. Based on the above results, we drew heat maps by using the Pheatmap R package (v 1.0.12) [73].

## Supplementary Material

nwaf219_Supplemental_Files

## Data Availability

All data needed to evaluate the conclusions in the paper are present in the Supplementary information. The raw metagenome reads utilized in this study have been made publicly accessible through their respective original publications as referenced in the Supplementary Table. The raw sequencing reads of the metatranscriptome have been deposited in the NCBI SRA (Sequence Read Archive) under BioProject accession number PRJNA1133194.
