# Supplementary material for: Non-linear frequency-doubling up-conversion in sulfide minerals enables deep-sea oxygenic photosynthesis
Source: Natl Sci Rev. 2025 May 28;12(6):nwaf219. doi: 10.1093/nsr/nwaf219 (PMC12202869; doi:10.1093/nsr/nwaf219)
Supplement: nwaf219_Supplemental_Files [file nwaf219_supplemental_files.zip › Li et al. revised SI.pdf]

Supplementary Materials of

Nonlinear Frequency Doubling Up-conversion in Sulfide Minerals Enables Deep-sea Oxygenic Photosynthesis

Yan Li[1,2]†*, Jiaqi Zhu[1,2]†, Qi Li[3]†, Hao Hong[4]†, Tao Li[3]*, Haoning Jia[1,2], Bingxu Hou[1,2], Houze Lu[1,2], Yanzhang Li[1,2], Jin Xie[4,5], Fuchen Wang[3], Huan Ye[1,2], Kaihui Liu[4,5]*, Anhuai Lu[1,2]*, Jindong Zhao[3,6]*

[1] State Key Laboratory of Deep Earth and Mineral Exploration, School of Earth and Space Sciences, Peking University, Beijing 100871, China.

[2] Beijing Key Laboratory of Mineral Environmental Function, School of Earth and Space Sciences, Peking University, Beijing 100871, China.

[3] Key Laboratory of Algal Biology, Institute of Hydrobiology, Chinese Academy of Sciences, Wuhan 430072, China.

[4] State Key Laboratory for Mesoscopic Physics, Frontiers Science Centre for Nano-optoelectronics, School of Physics, Peking University, Beijing 100871, China.

[5] International Centre for Quantum Materials, Collaborative Innovation Centre of Quantum Matter, Peking University, Beijing 100871, China.

[6] State Key Laboratory of Gene Function and Modulation Research, School of Life Sciences, Peking University, Beijing 100871, China.

†These authors contributed equally to this work.

*Correspondence to: liyan-pku@pku.edu.cn; litao@ihb.ac.cn; khliu@pku.edu.cn; ahlu@pku.edu.cn; jzhao@pku.edu.cn.

SUPPLEMENTARY METHODS

Sample preparation and phase characterization

The deep-sea black chimney samples were collected from the Longqi hydrothermal area in the Southwestern Indian Ocean, and other sulfide mineral samples were mainly from the Beijing Key Laboratory of Mineral Environmental Function, Peking University. XRD was conducted using an X-ray diffractometer (X pert pro, Philips). The applied voltage was 40 kV, and the current was 40 Ma. The X-ray source was Cu anode with the characteristic wavelength of 0.15406 nm ($K\alpha_1$). The step size was 0.017° (2θ) in the scanning range of 3-90°. The scanning speed is 3°/min.

Second harmonic generation (SHG) measurement

SHG measurement was conducted through the home-made and well-designed system. During the SHG measurement of natural black chimney samples and other sulfides, the incident light source was generated by a WhiteLase_SC400_UV supercontinuum white light system (Fundamental Pulse width = 6 ps, Fundamental Repetition Rate = 40 MHz). For the conversion efficiency comparison experiments of chalcopyrite ($CuFeS_2$), bismuthinite (Bi_2S_3), stibnite (Sb_2S_3), orpiment (As_2S_3) and molybdenite (MoS_2), the incident light source was generated by an optical parameter oscillator (Mira-OPO-X, Coherent), pumped by a titanium-sapphire oscillator (Mira-HP, Coherent) with Fundamental Pulse width = 130 fs, Fundamental Repetition Rate = 76 MHz. The spot size of the pump laser is about 10 μm^2. SHG measurements use a reflective geometry with the excitation laser perpendicular to the sample at room temperature. The generated SHG signals were collected by spectrometer Princeton Instruments, SP2500 and PyLoN 400 BRX with a 20× objective (×20, Nikon). The monochromatic light signal was obtained by placing a long pass filter or a short pass filter according to the desired wavelength. The number of emitted photons during the integration time was obtained from the peak area integration, and the output of light power collected by the spectrometer was estimated by the corresponding peak wavelength. Incident optical power was determined by an optical power meter (PM20CH, Thorlab).

Calculation of energy conversion efficiency in sulfide minerals

The energy conversion efficiency is equal to the ratio of the original outgoing light power to the incident light. When excited by a laser (λ_{ex}, P_{ex}), sulfide minerals exhibited the secondary harmonic generation (SHG) effect and emitted photons with twice the energy of the initial photons (i.e., λ_{SHG}=1/2 λ_{ex}). Based on the collected emission spectra, the SHG photon count (N) in each time (t) can be obtained through peak fitting and integration of peak area. Considering the quantum efficiency of spectrometer (55%), the fiber coupling efficiency (60%) and the loss efficiency of beam splitter prism (50%), the total SHG photons generated by minerals (N_{total}) can be determined by Equation (1):

$$N_{total} = N / (55\% \times 60\% \times 50\%) \quad (1)$$

The average power of mineral SHG (P_{SHG}) can be calculated by Equation (2):

$$P_{SHG} = N_{total} \times \frac{hc}{\lambda_{SHG}} \times \frac{1}{t} \tag{2}$$

Here, h is Planck constant and c is velocity of light.

The SHG energy conversion efficiency (η_{SHG}) of minerals can be calculated by Equation (3):

$$\eta_{SHG} = \frac{P_{SHG}}{p_{ex}} \tag{3}$$

First-principles calculations

The first-principles calculations based on density functional theory (DFT) were performed by means of the projected augmented wave method using Vienna Ab initio Simulation Package [1-5]. The generalized gradient approximations (GGA) with the Perdew−Burke−Ernzerhof (PBE) version were adopted to treat the exchange and correlation (XC) functional [6]. GGA + U method was used to treat localized strongly correlated electrons, and the Coulomb parameter U and exchange parameter J was set as 2 and 0 for both Cu and Fe. We employed pseudopotentials to model the ion-electron interaction. Pseudoatom calculations are performed for Cu-$3d^{10}4s^{1}$, for Fe-$3d^{6}4s^{2}$, and S-$3s^{2}3p^{4}$. The plane wave energy cutoff was set to 800 eV and the energy convergence criterion for the electronic self-consistent calculations was 10^{-7} eV. The force difference was converged to 1×10^{-3} eV/Å (less than 0.1 GPa). The k-points grids were set as 4 × 4 × 2 in the Brillouin zone using the Monkhorst-Pack method for geometry optimitation, and 8 × 8 × 4 for statistic calculation [7]. The high-symmetry points in the Brillouin zones were considered in our band structure calculations.

Computed tomography (CT) reconstruction

The CT reconstruction of the black chimney sample was performed using a high-resolution three-dimensional X-ray microscopy imaging system (SkyScan 1272, Bruker). The source voltage was set at 100 kV with a source current of 45 μA. The image resolution was 2 μm, and the single exposure time was 5400 ms. A single image consisted of 2264 × 2236 pixels. The reconstruction angle range was 188.10°, and the scan step size was 0.3°.

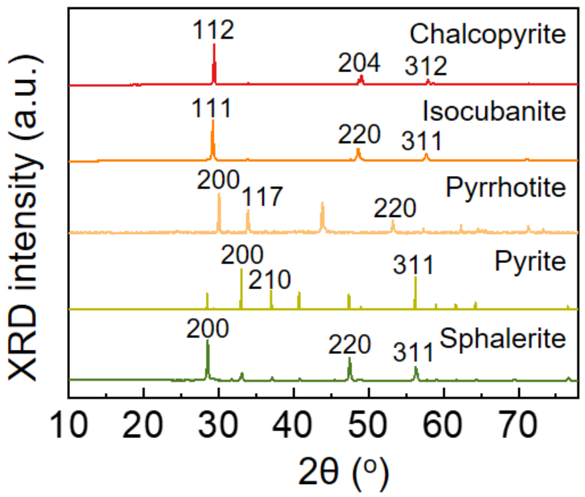

Figure S1. X-ray diffraction patterns of deep-sea sulfide minerals consisting of chalcopyrite, isocubanite, pyrrhotite, pyrite and sphalerite.

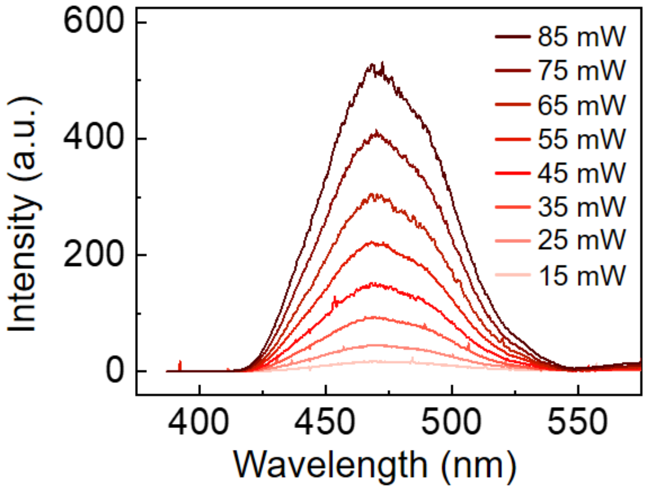

Figure S2. Emission spectra of chalcopyrite under a supercontinuum laser (800-1500 nm) with different excitation powers. With increasing power, the wavelength of the emission peak remained constant at 471 nm and the peak intensity increased proportionally.

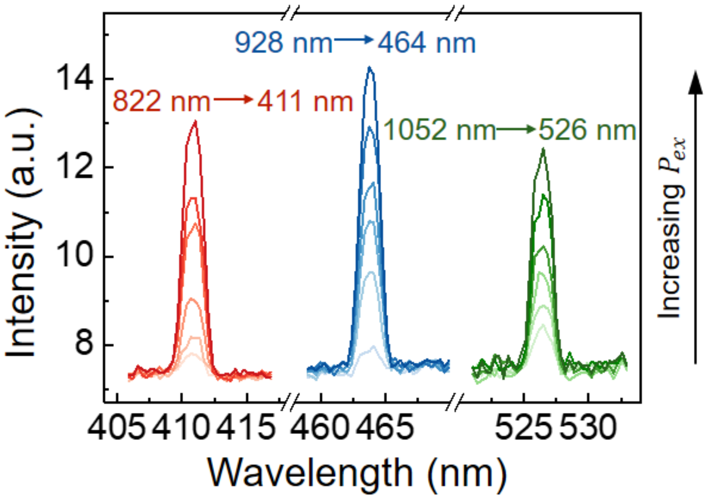

Figure S3. With increasing excitation power (P_{ex}, 0.45-3.20 mW), the emission peak remained at half of the excitation wavelength, and the enhanced intensity was proportional to P_{ex}.

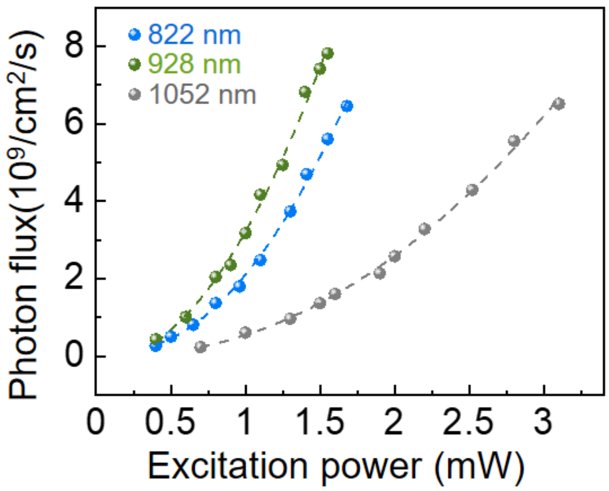

Figure S4. Excitation power-dependent SHG photon flux in chalcopyrite. SHG photon flux at three monochromatic wavelengths (822, 928 and 1052 nm) was represented by blue, green and grey sphere, respectively. With increasing light power, photon flux at each wavelength exhibited quadratic law, represented in blue, green and grey dashed curve, respectively.

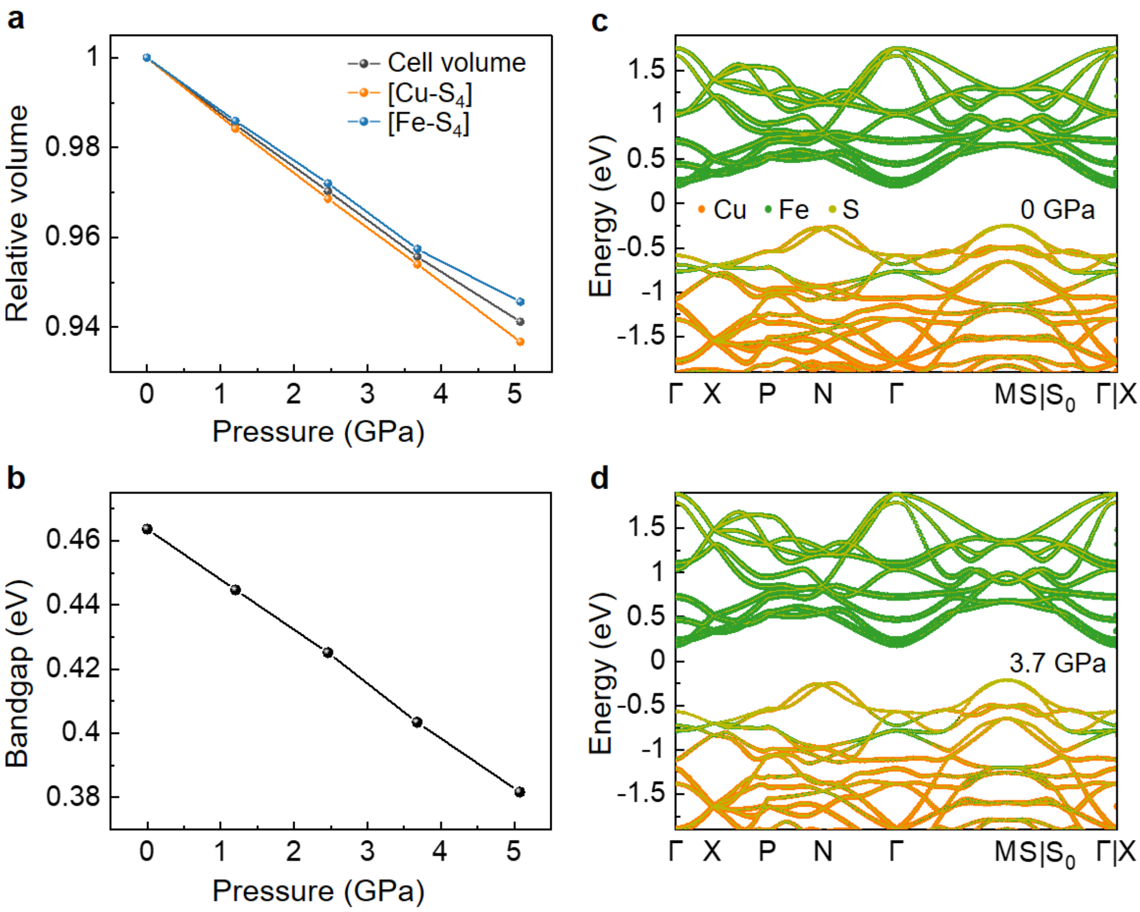

Figure S5. Theoretical calculations of chalcopyrite under pressure. (a) Decreased volume of copper-sulfur tetrahedron, iron-sulfur tetrahedron and cell in chalcopyrite with increasing pressure. (b) Bandgap of chalcopyrite under different pressure. (c) Electronic structure of chalcopyrite under ambient pressure. (d) Electronic structure of chalcopyrite under 3.7 GPa.

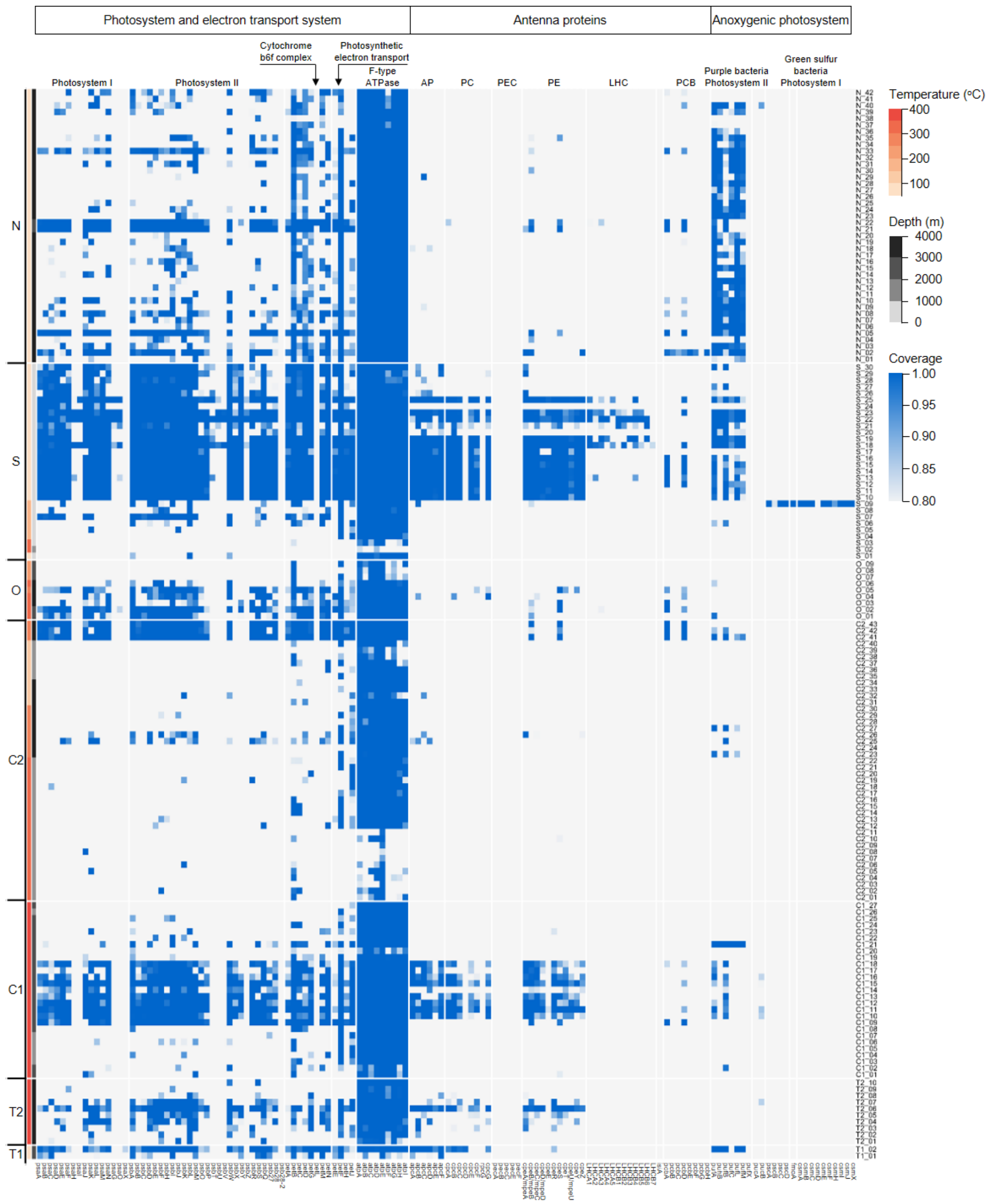

Figure S6. Metagenomic analysis of photosynthesis-related genes in 163 samples from different marine systems.

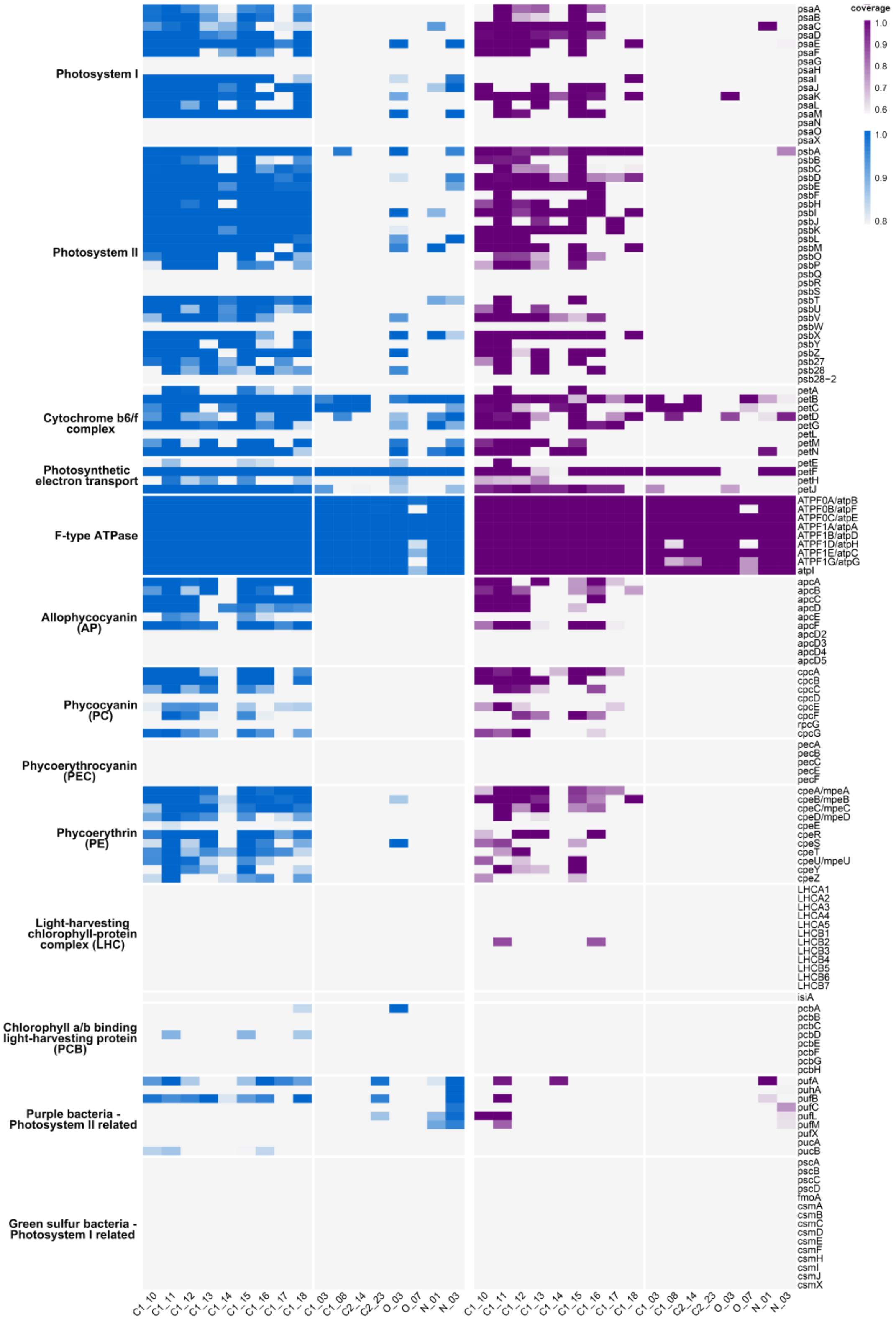

Figure S7. Metagenomic analysis of photosynthesis-related genes. Left panel (blue): the distribution of photosynthetic genes identified through alignment of metagenomic reads (threshold: ⩾80% sequence identity, ⩾80% coverage). Right panel (purple): the distribution of photosynthetic genes detected from metagenomic assemblies (threshold: ⩾80% sequence identity, ⩾60% coverage).

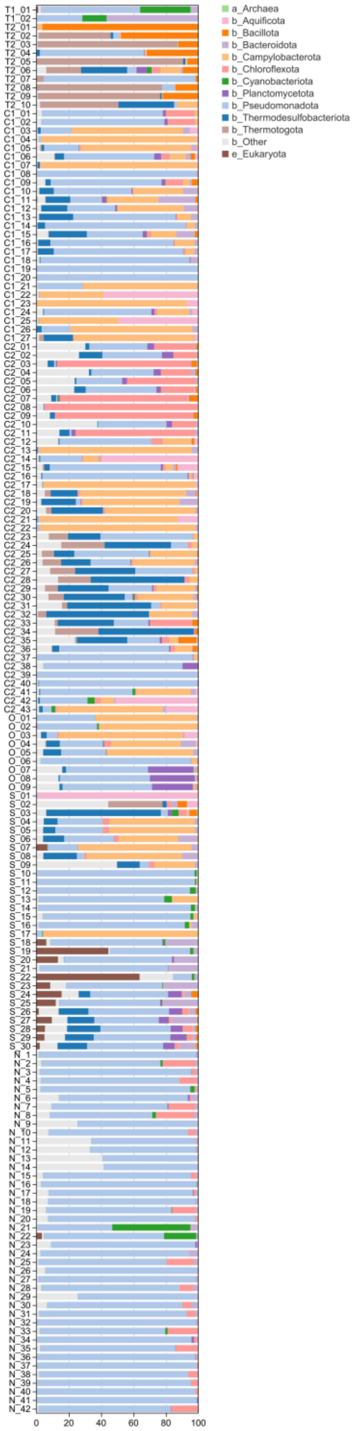

Figure S8. Taxonomic structure and community composition of metagenomic samples from different marine systems.

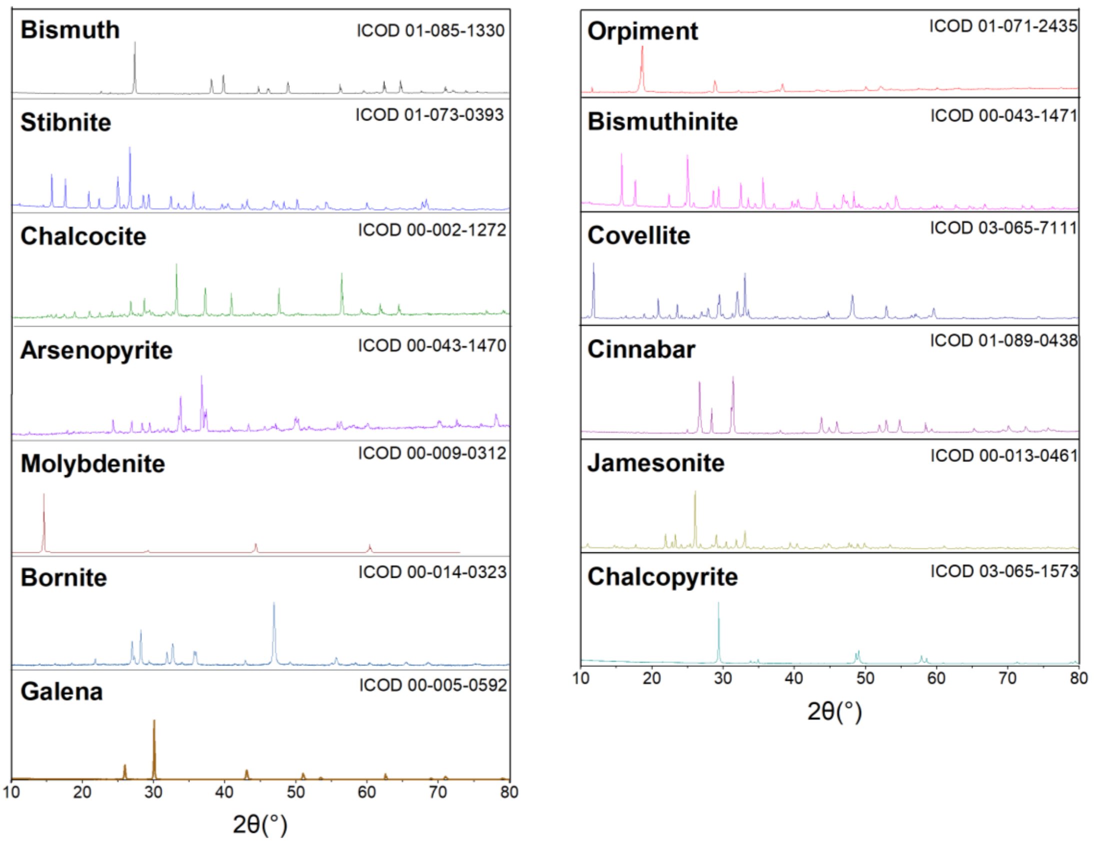

Figure S9. X-ray diffraction patterns of some selected sulfide minerals in SHG measurements.

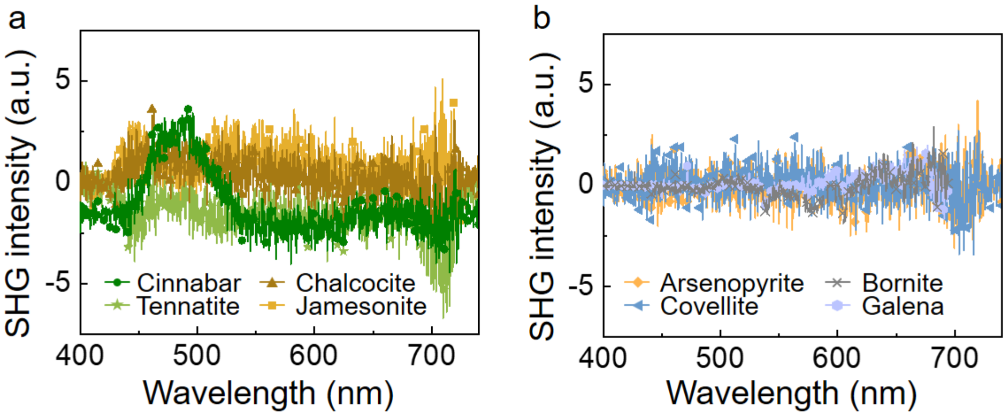

Figure S10. SHG emission spectra of eight sulfide minerals.

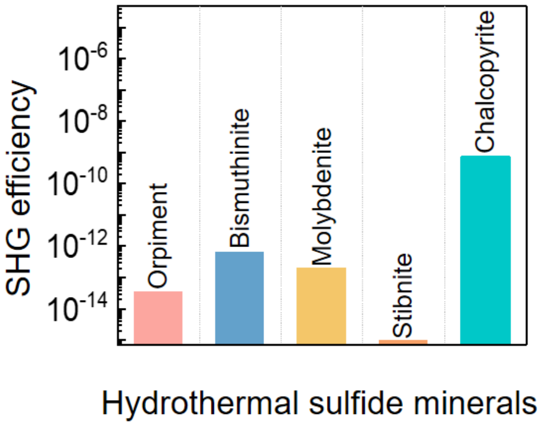

Figure S11. SHG Efficiency comparison of five sulfide minerals (orpiment, bismuthinite, molybdenite, stibnite and chalcopyrite) at their respective strongest emission peaks under an identical excitation power of 1.5 mW.

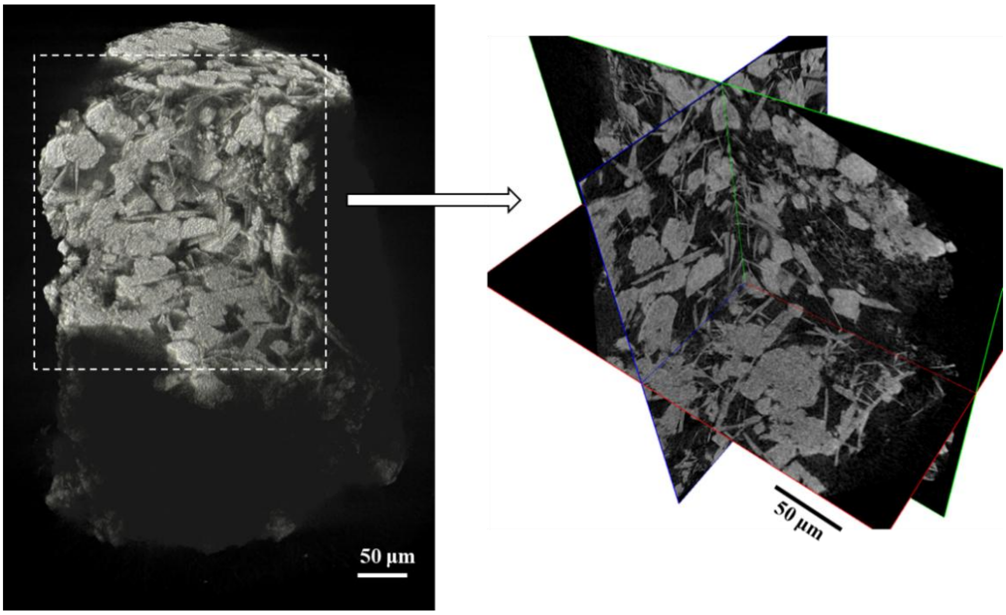

Figure S12. Local three-dimension computed tomography of the black chimney sample from Longqi hydrothermal area.

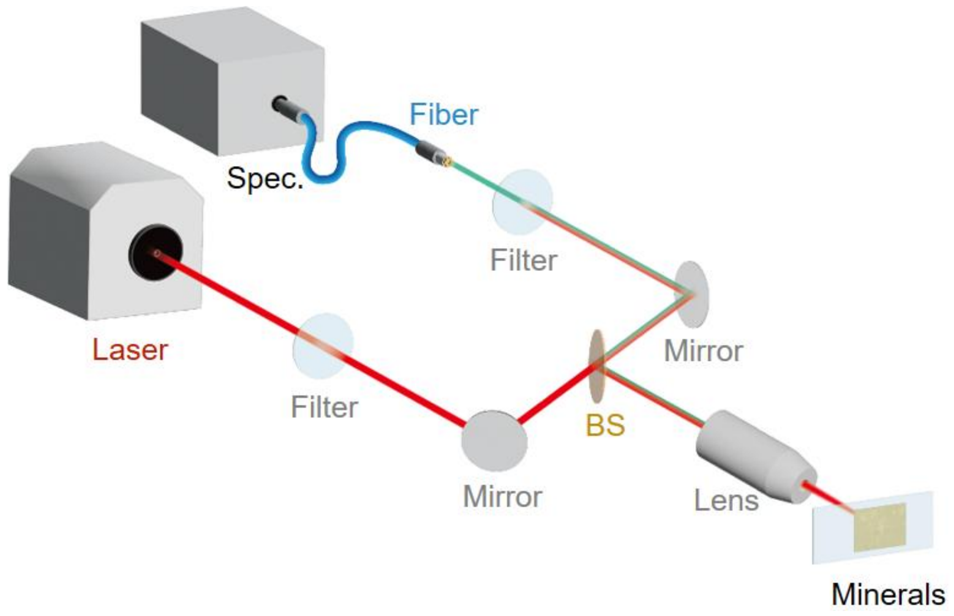

Figure S13. Schematic of the SHG experiment setup with reflective geometry. A 20× objective lens (Nikon) was used for laser focusing and SHG signal collection. After filtering out the excitation laser, SHG signal from minerals was recorded by a spectrometer (Spec.). BS: beam splitter.

REFERENCES

1. Hohenberg P, Kohn W. Inhomogeneous electron gas. *Phys Rev*. 1964; **136**(3B): B864.
2. Kohn W, Sham LJ. Self-consistent equations including exchange and correlation effects. *Phys Rev*. 1965; **140**(4A): A1133.
3. Kresse G, Furthmüller J. Efficiency of ab-initio total energy calculations for metals and semiconductors using a plane-wave basis set. *Comput Mater Sci*. 1996; **6**(1): 15-50.
4. Kresse G, Furthmüller J. Efficient iterative schemes for ab initio total-energy calculations using a plane-wave basis set. *Phys Rev B*. 1996; **54**(16): 11169.
5. Kresse G, Hafner J. Ab initio molecular dynamics for liquid metals. *Phys Rev B*. 1993; **47**(1): 558.
6. Perdew JP, Burke K, Ernzerhof M. Generalized gradient approximation made simple. *Phys Rev Lett*. 1996; **77**(18): 3865.
7. Monkhorst HJ, Pack JD. Special points for Brillouin-zone integrations. *Phys Rev B*. 1976; **13**(12): 5188.
