# Supplementary material for: Non-linear frequency-doubling up-conversion in sulfide minerals enables deep-sea oxygenic photosynthesis
Source: Natl Sci Rev. 2025 May 28;12(6):nwaf219. doi: 10.1093/nsr/nwaf219 (PMC12202869; doi:10.1093/nsr/nwaf219)
Supplement: nwaf219_Supplemental_Files [file nwaf219_supplemental_files.zip › Supplementary_Data1.pdf]

Sample	Sample	Latitude	Longitude	Depth	Temperature	Mineral	BioProject
T1_subsurface420	T1_01	37.729284	6.556527	D2	G	T1	PRJNA476489
T1_subsurface607	T1_02	37.729284	6.556527	D2	G	T1	PRJNA476489
T2_SRR13776092	T2_01	27.04618	-111.38483	D1	A	T2	PRJNA635695
T2_SRR13776093	T2_02	27.04618	-111.38483	D1	A	T2	PRJNA635695
T2_SRR13776094	T2_03	27.04618	-111.38483	D1	A	T2	PRJNA635695
T2_SRR13776095	T2_04	27.04618	-111.38483	D1	A	T2	PRJNA635695
T2_SRR13776096	T2_05	27.04618	-111.38483	D1	A	T2	PRJNA635695
T2_SRR13776097	T2_06	27.04618	-111.38483	D1	A	T2	PRJNA635695
T2_SRR13776098	T2_07	27.04618	-111.38483	D1	A	T2	PRJNA635695
T2_SRR13776099	T2_08	27.04618	-111.38483	D1	A	T2	PRJNA635695
T2_SRR13776100	T2_09	27.04618	-111.38483	D1	A	T2	PRJNA635695
T2_SRR13776101	T2_10	27.04618	-111.38483	D1	A	T2	PRJNA635695
C1_ERR4856265	C1_01	36.229	-33.902	D2	A	C1	PRJEB41358
C1_ERR4856266	C1_02	36.229	-33.902	D2	A	C1	PRJEB41358
C1_SRR13622958	C1_03	-22.1801	-176.6015	D3	A	C1	PRJNA495050
C1_SRR13622959	C1_04	-22.1802	-176.6012	D3	A	C1	PRJNA495050
C1_SRR13853571	C1_05	-22.1802	-176.6012	D3	A	C1	PRJNA495050
C1_SRR18689979	C1_06	-22.2157	-176.6086	D3	A	C1	PRJNA495050
C1_SRR18691003	C1_07	-22.1803	-176.6017	D3	A	C1	PRJNA495050
C1_SRR2080392	C1_08	18.54478	-81.7195	D2	A	C1	PRJNA283159
C1_SRR2080393	C1_09	18.54478	-81.7195	D2	A	C1	PRJNA283159
C1_SRR21545380	C1_10	27.00647191	-111.40935798	D2	A	C1	PRJNA879229
C1_SRR21545381	C1_11	27.00647191	-111.409358	D2	A	C1	PRJNA879229
C1_SRR21545382	C1_12	27.00647191	-111.409358	D2	A	C1	PRJNA879229
C1_SRR21545384	C1_13	27.00647191	-111.409358	D2	A	C1	PRJNA879229
C1_SRR21545385	C1_14	27.00647191	-111.409358	D2	A	C1	PRJNA879229
C1_SRR21545386	C1_15	27.00647191	-111.409358	D2	A	C1	PRJNA879229
C1_SRR21545387	C1_16	27.00647191	-111.409358	D2	A	C1	PRJNA879229
C1_SRR21545389	C1_17	27.00647191	-111.409358	D2	A	C1	PRJNA879229
C1_SRR3577362	C1_18	27.515833	-111.425	D2	A	C1	PRJNA314399
C1_SRR452448	C1_19	27.5060	-111.34697	D2	A	C1	PRJNA72707
C1_SRR453184	C1_20	27.4862	-111.36407	D2	A	C1	PRJNA72707
C1_SRR7968105	C1_21	-22.1802	-176.6008	D2	A	C1	PRJNA469232
C1_SRR7968106	C1_22	27.0078	-111.4071	D2	A	C1	PRJNA469234
C1_SRR7968107	C1_23	27.0078	-111.4071	D2	A	C1	PRJNA469235
C1_SRR7968108	C1_24	36.2297	-33.9011	D2	A	C1	PRJNA469237
C1_SRR7968109	C1_25	27.0105	-111.4068	D2	A	C1	PRJNA469236
C1_SRR7968110	C1_26	36.2294	-33.9028	D3	A	C1	PRJNA469238
C1_SRR7968125	C1_27	27.0067	-111.4094	D2	A	C1	PRJNA469240
C2_DRR122308	C2_01	27.77	126.90	D3	B	C2	PRJDB6717
C2_DRR122309	C2_02	27.77	126.90	D3	B	C2	PRJDB6717
C2_DRR122310	C2_03	27.77	126.90	D3	B	C2	PRJDB6717
C2_DRR122311	C2_04	27.77	126.90	D3	B	C2	PRJDB6717
C2_DRR122312	C2_05	27.77	126.90	D3	B	C2	PRJDB6717
C2_DRR122313	C2_06	27.77	126.90	D3	B	C2	PRJDB6717
C2_DRR122314	C2_07	27.77	126.90	D3	B	C2	PRJDB6717
C2_DRR122315	C2_08	27.77	126.90	D3	B	C2	PRJDB6717
C2_DRR122316	C2_09	27.77	126.90	D3	B	C2	PRJDB6717
C2_DRR122317	C2_10	27.77	126.90	D3	B	C2	PRJDB6717
C2_DRR122318	C2_11	27.77	126.90	D3	B	C2	PRJDB6717
C2_SRR10309773	C2_12	-34.862587	179.058108	D3	B	C2	PRJNA546572
C2_SRR10310285	C2_13	-34.88234384	179.0682732	D3	B	C2	PRJNA546572
C2_SRR10312489	C2_14	-34.86111866	179.0576651	D3	B	C2	PRJNA546572
C2_SRR10312490	C2_15	-34.86150855	179.0573001	D3	B	C2	PRJNA546572
C2_SRR10312491	C2_16	-34.85791311	179.0519346	D3	B	C2	PRJNA546572
C2_SRR10312492	C2_17	-34.861904	179.057875	D3	B	C2	PRJNA546572
C2_SRR10312493	C2_18	-34.878866	179.071394	D3	B	C2	PRJNA546572

C2_SRR1031249[illegible]	C2_19	-34.861552	179.057153	D3	B	C2	PRJNA546572
C2_SRR1031249[illegible]	C2_20	-34.878368	179.071443	D3	B	C2	PRJNA546572
C2_SRR1031249[illegible]	C2_21	-34.86107892	179.0576362	D3	B	C2	PRJNA546572
C2_SRR1031249[illegible]	C2_22	-34.882356	179.0682646	D3	B	C2	PRJNA546572
C2_SRR1400003[illegible]	C2_23	23.957043	-108.862273	D1	C	C2	PRJNA713414
C2_SRR1400003[illegible]	C2_24	23.957043	-108.862273	D1	C	C2	PRJNA713414
C2_SRR1400003[illegible]	C2_25	23.957043	-108.862273	D1	C	C2	PRJNA713414
C2_SRR1400003[illegible]	C2_26	23.957043	-108.862273	D1	C	C2	PRJNA713414
C2_SRR1400003[illegible]	C2_27	23.957043	-108.862273	D1	C	C2	PRJNA713414
C2_SRR1400003[illegible]	C2_28	23.957043	-108.862273	D1	C	C2	PRJNA713414
C2_SRR1400003[illegible]	C2_29	23.957043	-108.862273	D1	C	C2	PRJNA713414
C2_SRR1400003[illegible]	C2_30	23.957043	-108.862273	D1	C	C2	PRJNA713414
C2_SRR2141855[illegible]	C2_31	23.954027	-108.863324	D1	F	C2	PRJNA875076
C2_SRR2141855[illegible]	C2_32	23.942356	-108.855825	D1	F	C2	PRJNA875076
C2_SRR2141855[illegible]	C2_33	23.954036	-108.86296	D1	F	C2	PRJNA875076
C2_SRR2141856[illegible]	C2_34	23.956094	-108.86192	D1	F	C2	PRJNA875076
C2_SRR5189948	C2_35	18.902	-155.257	D3	F	C2	PRJNA361177
C2_SRR5189949	C2_36	18.902	-155.257	D3	F	C2	PRJNA361177
C2_SRR5189950	C2_37	18.902	-155.257	D3	F	C2	PRJNA361177
C2_SRR5189951	C2_38	18.903	-155.257	D3	F	C2	PRJNA361177
C2_SRR5189952	C2_39	18.903	-155.257	D3	F	C2	PRJNA361177
C2_SRR5189953	C2_40	18.903	-155.257	D3	F	C2	PRJNA361177
C2_SRR7168047	C2_41	18.21359	144.70748	D1	C	C2	PRJNA454888
C2_SRR7168048	C2_42	18.210325	-144.707309	D1	C	C2	PRJNA454888
C2_SRR7168049	C2_43	18.182568	-144.719893	D1	C	C2	PRJNA454888
O_DRR354676	O_01	27.7916	126.8967	D2	B	O	PRJDB13230
O_DRR354677	O_02	27.7916	126.8967	D2	B	O	PRJDB13230
O_ERR2021503	O_03	45.9332	-129.9822	D1	B	O	PRJEB19456
O_ERR2021505	O_04	45.9332	-130.0139	D1	B	O	PRJEB19456
O_ERR2021509	O_05	46.0747	-129.9950	D1	C	O	PRJEB19456
O_ERR2021511	O_06	45.9335667	-130.013667	D1	B	O	PRJEB19456
O_ERR2834528	O_07	36.52	25.48	D2	D	O	PRJEB29105
O_ERR2834530	O_08	36.52	25.48	D2	D	O	PRJEB29105
O_ERR2834531	O_09	36.52	25.48	D2	D	O	PRJEB29105
S_SRR10484890	S_01	-37.5248	177.1902	D4	G	S	PRJNA514927
S_SRR18053868	S_02	27.79026	126.900677	D3	B	S	PRJNA807686
S_SRR18053869	S_03	27.79026	126.900677	D4	B	S	PRJNA807686
S_SRR19843165	S_04	24.83422	121.96232	D4	E	S	PRJNA851985
S_SRR19843166	S_05	24.83421	121.96232	D4	E	S	PRJNA851985
S_SRR19843167	S_06	24.8342	121.96232	D4	E	S	PRJNA851985
S_SRR5149596	S_07	24.83499	121.96209	D4	E	S	PRJNA851985
S_SRR5149604	S_08	24.83471	121.96168	D4	E	S	PRJNA355347
S_SRR5149607	S_09	24.83499	121.96207	D4	E	S	PRJNA355347
S_SRR5229878	S_10	24.8344	121.96201	D4	G	S	PRJNA355347
S_SRR5229879	S_11	24.83414	121.96191	D4	G	S	PRJNA355347
S_SRR5229880	S_12	24.83414	121.96191	D4	G	S	PRJNA355347
S_SRR5229881	S_13	24.83414	121.96191	D4	G	S	PRJNA355347
S_SRR5229882	S_14	24.83478	121.96208	D4	G	S	PRJNA355347
S_SRR5229883	S_15	24.83499	121.96207	D4	G	S	PRJNA355347
S_SRR5229884	S_16	24.83499	121.96207	D4	G	S	PRJNA355347
S_SRR5229885	S_17	24.83499	121.96207	D4	G	S	PRJNA355347
S_SRR6441338	S_18	35.5	121.67	D4	G	S	PRJNA428417
S_SRR6441339	S_19	35.5	121.67	D4	G	S	PRJNA428417
S_SRR6441342	S_20	35.5	124.00	D4	G	S	PRJNA428417
S_SRR6441343	S_21	35.5	124.00	D4	G	S	PRJNA428417
S_SRR6441344	S_22	35.5	124.00	D4	G	S	PRJNA428417
S_SRR6441345	S_23	35.5	124.00	D4	G	S	PRJNA428417
S_SRR6441346	S_24	35.5	121.67	D4	G	S	PRJNA428417

S_SRR6441347	S_25	35.5	121.67	D4	G	S	PRJNA428417
S_SRR8443351	S_26	39.31	119.71	D4	G	S	PRJNA514927
S_SRR8443352	S_27	38.67	118.97	D4	G	S	PRJNA514927
S_SRR8443353	S_28	38.32	119.0	D4	G	S	PRJNA514927
S_SRR8443354	S_29	38.33	120.18	D4	G	S	PRJNA514927
S_SRR8443355	S_30	37.69	122.47	D4	G	S	PRJNA514927
N_SRR3960572	N_1	32.0788	-17.2662	D1	G	N	PRJNA330077
N_SRR3960575	N_2	19.9897	-52.6367	D1	G	N	PRJNA329999
N_SRR3960576	N_3	29.97	-23.69	D1	G	N	PRJNA330096
N_SRR3960577	N_4	32.0788	-17.2662	D1	G	N	PRJNA330149
N_SRR3960578	N_5	29.97	-23.69	D1	G	N	PRJNA330129
N_SRR3960580	N_6	19.9897	-52.6367	D1	G	N	PRJNA330088
N_SRR3961906	N_7	15.9087	-124.4738	D1	G	N	PRJNA330152
N_SRR3961935	N_8	18.04	-133.26	D1	G	N	PRJNA330009
N_SRR3961936	N_9	9.22	-163.53	D1	G	N	PRJNA330136
N_SRR3962293	N_10	9.22	-163.53	D1	G	N	PRJNA330126
N_SRR3962508	N_11	15.9087	-124.4738	D1	G	N	PRJNA330106
N_SRR3962771	N_12	21.0638	-150.3192	D1	G	N	PRJNA330119
N_SRR3962772	N_13	-5.75	-170.7407	D1	G	N	PRJNA330080
N_SRR3963457	N_14	-30.3327	103.3075	D1	G	N	PRJNA330001
N_SRR3963458	N_15	-39.23	135.19	D1	G	N	PRJNA330113
N_SRR3963498	N_16	-31.16	110.18	D1	G	N	PRJNA330140
N_SRR3963571	N_17	-30.3327	103.3075	D1	G	N	PRJNA330138
N_SRR3963599	N_18	-38.64	150.41	D1	G	N	PRJNA329988
N_SRR3963622	N_19	-29.81	82.62	D1	G	N	PRJNA330020
N_SRR3963658	N_20	-29.81	82.62	D1	G	N	PRJNA330091
N_SRR3963804	N_21	-29.6525	92.9852	D2	G	N	PRJNA329989
N_SRR3963805	N_22	-29.6525	92.9852	D2	G	N	PRJNA329993
N_SRR3965585	N_23	-33.55	39.89	D1	G	N	PRJNA330081
N_SRR3965586	N_24	-33.55	39.89	D1	G	N	PRJNA330006
N_SRR3965592	N_25	-26.91	-21.43	D1	G	N	PRJNA330027
N_SRR3965647	N_26	-31.81	6.84	D1	G	N	PRJNA330002
N_SRR3965676	N_27	-26.91	-21.43	D1	G	N	PRJNA330095
N_SRR3965758	N_28	-31.81	6.84	D1	G	N	PRJNA330094
N_SRR3965809	N_29	-32.8128	12.7692	D1	G	N	PRJNA330019
N_SRR3965873	N_30	-32.8128	12.7692	D1	G	N	PRJNA330079
N_SRR3966130	N_31	-22.97	-36.95	D1	G	N	PRJNA330097
N_SRR3967025	N_32	-22.97	-36.95	D1	G	N	PRJNA330154
N_SRR3967319	N_33	-15.83	-33.41	D1	G	N	PRJNA330007
N_SRR3967558	N_34	-15.83	-33.41	D1	G	N	PRJNA330083
N_SRR3967690	N_35	-9.12	-30.19	D1	G	N	PRJNA330161
N_SRR3967699	N_36	-9.12	-30.19	D1	G	N	PRJNA330089
N_SRR3967905	N_37	14.52	-26	D1	G	N	PRJNA330135
N_SRR3968061	N_38	14.52	-26	D1	G	N	PRJNA330017
N_SRR3968062	N_39	7.33	-26	D1	G	N	PRJNA330139
N_SRR3968063	N_40	7.33	-26	D1	G	N	PRJNA329987
N_SRR3968770	N_41	21.51	-23.45	D1	G	N	PRJNA330127
N_SRR3968777	N_42	21.51	-23.45	D1	G	N	PRJNA330076

D1: depth <100 m; D2: 100 m <depth <1500 m; D3: 1500 m <depth <4000 m.

A: temperature >350°C; B: 300°C <temperature <350°C; C: 250°C <temperature <300°C; D: 200°C <temperature <250°C; E: 150°C <temperature <200°C; F: 100°C <temperature <150°C; G: 0°C <temperature <100°C.

T1: deep surface rock samples; T2: thermophilic microbial community after enrichment culture; C1: hydrothermal vent with central temperature above 350°C; C2: hydrothermal vents with central temperature below 350°C; O: thermophilic enrichment culture from oxidation zone; S: shallow hydrothermal vents in
