# Supplementary material for: Non-linear frequency-doubling up-conversion in sulfide minerals enables deep-sea oxygenic photosynthesis
Source: Natl Sci Rev. 2025 May 28;12(6):nwaf219. doi: 10.1093/nsr/nwaf219 (PMC12202869; doi:10.1093/nsr/nwaf219)
Supplement: nwaf219_Supplemental_Files [file nwaf219_supplemental_files.zip › Supplementary_Data2.pdf]

Gene_Organisms: apcA

**Kingdom**	**Phylum**	**Class**	**Order**	**Family**	**Gunus**	**Species**
Bacteria	Cyanobacteriota	Cyanophyceae	Nostocales	Nostocaceae	Anabaena	Anabaena cylindrica
Bacteria	Cyanobacteriota	Cyanophyceae	Nostocales	Nodulariaceae	Anabaenopsis	Anabaenopsis elenkinii
Bacteria	Cyanobacteriota	Cyanophyceae	Nostocales	Aphanizomenonaceae	Aphanizomenon	Aphanizomenon flos-aquae
Bacteria	Cyanobacteriota	Cyanophyceae	Nostocales	Fortieaceae	Aulosira	Aulosira laxa
Bacteria	Cyanobacteriota	Cyanophyceae	Nostocales	Nostocaceae	Nostoc	Nostoc sp.
Bacteria	Cyanobacteriota	Cyanophyceae	Nostocales	Nostocaceae	Anabaena	Anabaena sp.
Bacteria	Cyanobacteriota	Cyanophyceae	Oscillatoriales	Microcoleaceae	Arthrospira	Arthrospira platensis
Bacteria	Cyanobacteriota	Cyanophyceae	Nostocales	Nostocaceae	Trichormus	Trichormus variabilis
Bacteria	Cyanobacteriota	Cyanophyceae	Nostocales	Scytonemataceae	Brasilonema	Brasilonema octagenarum
Bacteria	Cyanobacteriota	Cyanophyceae	Nostocales	Scytonemataceae	Brasilonema	Brasilonema sennae
Bacteria	Cyanobacteriota	Cyanophyceae	Nostocales	Calotrichaceae	Calothrix	Calothrix sp.
Bacteria	Cyanobacteriota	Cyanophyceae	Chroococcales	Geminocystaceae	Cyanobacterium	Cyanobacterium aponinum
Bacteria	Cyanobacteriota	Cyanophyceae	Nostocales	Aphanizomenonaceae	Cylindrospermopsis	Cylindrospermopsis curvispora
Bacteria	Cyanobacteriota	Cyanophyceae	Gomontiellales	Gomontiellaceae	Crinalium	Crinalium epipsammum
Bacteria	Cyanobacteriota	Cyanophyceae	Synechococcales	Prochlorococcaceae	Cyanobium	Cyanobium gracile
Bacteria	Cyanobacteriota	Cyanophyceae	Chroococcales	Chroococcaceae	Chondrocystis	unclassified Chondrocystis species
Bacteria	Cyanobacteriota	Cyanophyceae	Gomontiellales	Chamaesiphonaceae	Chamaesiphon	Chamaesiphon minutus
Bacteria	Cyanobacteriota	Cyanophyceae	Nostocales	Aphanizomenonaceae	Cylindrospermopsis	Cylindrospermopsis raciborskii
Bacteria	Cyanobacteriota	Cyanophyceae	Nostocales	Nostocaceae	Cylindrospermum	Cylindrospermum stagnale
Bacteria	Cyanobacteriota	Cyanophyceae	Chroococcales	Geminocystaceae	Cyanobacterium	Cyanobacterium stanieri
Bacteria	Cyanobacteriota	Cyanophyceae	Chroococcidiopsidales	Chroococcidiopsidaceae	Chroococcidiopsis	Chroococcidiopsis thermalis
Bacteria	Cyanobacteriota	Cyanophyceae	Chroococcales	Aphanothecaceae	Crocosphaera	Crocosphaera watsonii
Bacteria	Cyanobacteriota	Cyanophyceae	Synechococcales	Synechococcaceae	Synechococcus	Synechococcus sp.
Bacteria	Cyanobacteriota	Cyanophyceae	Chroococcales	Aphanothecaceae	Gloeothece	Gloeothece citriformis
Bacteria	Cyanobacteriota	Cyanophyceae	Chroococcales	Aphanothecaceae	Rippkaea	Rippkaea orientalis
Bacteria	Cyanobacteriota	Cyanophyceae	Synechococcales	Prochlorococcaceae	Cyanobium	Cyanobium sp.
Bacteria	Cyanobacteriota	Cyanophyceae	Chroococcales	Aphanothecaceae	Gloeothece	Gloeothece verrucosa
Bacteria	Cyanobacteriota	Cyanophyceae	Chroococcales	Geminocystaceae	Cyanobacterium	Cyanobacterium sp.
Bacteria	Cyanobacteriota	Cyanophyceae	Gomontiellales	Cyanothecaceae	Cyanothece	Cyanothece sp.
Bacteria	Cyanobacteriota	Cyanophyceae	Chroococcales	Aphanothecaceae	Crocosphaera	Crocosphaera subtropica
Bacteria	Cyanobacteriota	Cyanophyceae	Nostocales	Aphanizomenonaceae	Dolichospermum	Dolichospermum compactum
Bacteria	Cyanobacteriota	Cyanophyceae	Nostocales	Aphanizomenonaceae	Dolichospermum	Dolichospermum flos-aquae
Bacteria	Cyanobacteriota	Cyanophyceae	Nostocales	Aphanizomenonaceae	Dolichospermum	Dolichospermum heterosporum
Bacteria	Cyanobacteriota	Cyanophyceae	Nostocales	Aphanizomenonaceae	Dolichospermum	Dolichospermum sp.
Bacteria	Cyanobacteriota	Cyanophyceae	Synechococcales	Synechococcaceae	Dactylococcopsis	Dactylococcopsis salina
Bacteria	Cyanobacteriota	Cyanophyceae	Chroococcales	Halothecacae	Euhalothece	Euhalothece natronophila

Bacteria	Cyanobacteriota	Cyanophyceae	Nostocales	Hapalosiphonaceae	Fischerella	Fischerella sp.
Bacteria	Cyanobacteriota	Cyanophyceae	Chroococcales	Chroococcaceae	Gloeocapsopsis	Gloeocapsopsis dulcis
Bacteria	Cyanobacteriota	Cyanophyceae	Chroococcales	Geminocystaceae	Geminocystis	Geminocystis sp.
Bacteria	Cyanobacteriota	Cyanophyceae	Geitlerinematales	Geitlerinemataceae	Geitlerinema	Geitlerinema sp.
Bacteria	Cyanobacteriota	Cyanophyceae	Gloeobacterales	Gloeobacteraceae	Gloeobacter	Gloeobacter kilaueensis
Bacteria	Cyanobacteriota	Cyanophyceae	Chroococcales	Chroococcaceae	Gloeocapsa	unclassified Gloeocapsa species
Bacteria	Cyanobacteriota	Cyanophyceae	Gloeomargaritales	Gloeomargaritaceae	Gloeomargarita	Gloeomargarita lithophora
Bacteria	Cyanobacteriota	Cyanophyceae	Gloeobacterales	Gloeobacteraceae	Gloeobacter	Gloeobacter morelensis
Bacteria	Cyanobacteriota	Cyanophyceae	Gloeobacterales	Gloeobacteraceae	Gloeobacter	Gloeobacter violaceus
Bacteria	Cyanobacteriota	Cyanophyceae	Chroococcales	Halothecacae	Halothece	Halothece sp.
Bacteria	Cyanobacteriota	Cyanophyceae	Nostocales	Nodulariaceae	Halotia	Halotia branconii
Bacteria	Cyanobacteriota	Cyanophyceae	Nodosilineales	Nodosilineaceae	Halomicronema	Halomicronema hongdechloris
Bacteria	Cyanobacteriota	Cyanophyceae	Leptolyngbyales	Leptolyngbyaceae	Kovacikia	Kovacikia minuta
Bacteria	Cyanobacteriota	Cyanophyceae	Leptolyngbyales	Leptolyngbyaceae	Leptolyngbya	Leptolyngbya boryana
Bacteria	Cyanobacteriota	Cyanophyceae	Leptolyngbyales	Leptolyngbyaceae	Leptolyngbya	Leptolyngbya sp.
Bacteria	Cyanobacteriota	Cyanophyceae	Oscillatoriales	Sirenicapillariaceae	Limnospira	Limnospira fusiformis
Bacteria	Cyanobacteriota	Cyanophyceae	Oscillatoriales	Sirenicapillariaceae	Limnospira	Limnospira indica
Bacteria	Cyanobacteriota	Cyanophyceae	Leptolyngbyales	Leptolyngbyaceae	Leptodesmis	Leptodesmis sichuanensis
Bacteria	Cyanobacteriota	Cyanophyceae	Leptolyngbyales	Leptolyngbyaceae	Leptothermofonsia	Leptothermofonsia sichuanensis
Bacteria	Cyanobacteriota	Cyanophyceae	Chroococcales	Microcystaceae	Microcystis	Microcystis aeruginosa
Bacteria	Cyanobacteriota	Cyanophyceae	Nostocales	Rivulariaceae	Microchaete	Microchaete diplosiphon
Bacteria	Cyanobacteriota	Cyanophyceae	Coleofasciculales	Coleofasciculaceae	Allocoleopsis	Allocoleopsis franciscana
Bacteria	Cyanobacteriota	Cyanophyceae	Chroococcales	Microcystaceae	Microcystis	Microcystis sp.
Bacteria	Cyanobacteriota	Cyanophyceae	Chroococcales	Microcystaceae	Microcystis	Microcystis panniformis
Bacteria	Cyanobacteriota	Cyanophyceae	Oscillatoriales	Oscillatoriaceae	Moorena	Moorena producens
Bacteria	Cyanobacteriota	Cyanophyceae	Oscillatoriales	Microcoleaceae	Microcoleus	Microcoleus vaginatus
Bacteria	Cyanobacteriota	Cyanophyceae	Chroococcales	Microcystaceae	Microcystis	Microcystis viridis
Bacteria	Cyanobacteriota	Cyanophyceae	Nostocales	unclassified Nostocales family	unclassified Nostocales genus	Nostocales cyanobacterium
Bacteria	Cyanobacteriota	Cyanophyceae	Nostocales	Nostocaceae	Nostoc	Nostoc carneum
Bacteria	Cyanobacteriota	Cyanophyceae	Nostocales	Nostocaceae	Nostoc	Nostoc edaphicum
Bacteria	Cyanobacteriota	Cyanophyceae	Nostocales	Nostocaceae	Nostoc	Nostoc flagelliforme
Bacteria	Cyanobacteriota	Cyanophyceae	Nostocales	Nostocaceae	Nostoc	Nostoc linckia
Bacteria	Cyanobacteriota	Cyanophyceae	Nostocales	Nostocaceae	Nostoc	Nostoc punctiforme
Bacteria	Cyanobacteriota	Cyanophyceae	Nostocales	Nostocaceae	Nostoc	Nostoc piscinale
Bacteria	Cyanobacteriota	Cyanophyceae	Nostocales	Nostocaceae	Nostoc	Nostoc sphaeroides
Bacteria	Cyanobacteriota	Cyanophyceae	Nostocales	Nodulariaceae	Nodularia	Nodularia sphaerocarpa
Bacteria	Cyanobacteriota	Cyanophyceae	Oscillatoriales	Oscillatoriaceae	Oscillatoria	Oscillatoria acuminata
Bacteria	Cyanobacteriota	Cyanophyceae	Nostocales	Aphanizomenonaceae	Okeanomitos	Okeanomitos corallinicola

Bacteria	Cyanobacteriota	Cyanophyceae	Oscillatoriales	Oscillatoriaceae	Oscillatoria	Oscillatoria nigro-viridis
Bacteria	Cyanobacteriota	Cyanophyceae	Oscillatoriales	Oscillatoriaceae	Oxynema	Oxynema aestuarii
Bacteria	Cyanobacteriota	Cyanophyceae	Oscillatoriales	Microcoleaceae	Planktothrix	Planktothrix agardhii
Bacteria	Cyanobacteriota	Cyanophyceae	Pseudanabaenales	Pseudanabaenaceae	Pseudanabaena	Pseudanabaena galeata
Bacteria	Cyanobacteriota	Cyanophyceae	Oscillatoriales	Oscillatoriaceae	Phormidium	Phormidium sp.
Bacteria	Cyanobacteriota	Cyanophyceae	Chroococcales	Geminocystaceae	Picosynechococcus	unclassified Picosynechococcus species
Bacteria	Cyanobacteriota	Cyanophyceae	Pleurocapsales	Hyellaceae	Pleurocapsa	Pleurocapsa sp.
Bacteria	Cyanobacteriota	Cyanophyceae	Oscillatoriales	Microcoleaceae	Planktothrix	Planktothrix pseudagardhii
Bacteria	Cyanobacteriota	Cyanophyceae	Oscillatoriales	Microcoleaceae	Planktothrix	Planktothrix rubescens
Bacteria	Cyanobacteriota	Cyanophyceae	Pseudanabaenales	Pseudanabaenaceae	Pseudanabaena	Pseudanabaena sp.
Bacteria	Cyanobacteriota	Cyanophyceae	Oscillatoriales	Oscillatoriaceae	Phormidium	Phormidium yuhuli
Bacteria	Cyanobacteriota	Cyanophyceae	Nostocales	Aphanizomenonaceae	Raphidiopsis	Raphidiopsis curvata
Bacteria	Cyanobacteriota	Cyanophyceae	Nostocales	Nostocaceae	Richelia	Richelia sinica
Bacteria	Cyanobacteriota	Cyanophyceae	Pleurocapsales	Dermocarpellaceae	Stanieria	Stanieria cyanosphaera
Bacteria	Cyanobacteriota	Cyanophyceae	Nostocales	Scytonemataceae	Scytonema	Scytonema sp.
Bacteria	Cyanobacteriota	Cyanophyceae	Nostocales	Aphanizomenonaceae	Sphaerospermopsis	Sphaerospermopsis kisseleviana
Bacteria	Cyanobacteriota	Cyanophyceae	Acaryochloridales	Thermosynechococcaceae	Parathermosynechococcus	Parathermosynechococcus lividus
Bacteria	Cyanobacteriota	Cyanophyceae	Pleurocapsales	Dermocarpellaceae	Stanieria	Stanieria sp.
Bacteria	Cyanobacteriota	Cyanophyceae	Nostocales	Aphanizomenonaceae	Sphaerospermopsis	Sphaerospermopsis torques-reginae
Bacteria	Cyanobacteriota	Cyanophyceae	Synechococcales	Synechococcaceae	Synechococcus	Synechococcus elongatus
Bacteria	Cyanobacteriota	Cyanophyceae	Synechococcales	Merismopediaceae	Synechocystis	Synechocystis sp.
Bacteria	Cyanobacteriota	Cyanophyceae	Synechococcales	Prochlorococcaceae	Parasynechococcus	Parasynechococcus marenigrum
Bacteria	Cyanobacteriota	Cyanophyceae	Acaryochloridales	Thermosynechococcaceae	Thermosynechococcus	Thermosynechococcus vestitus
Bacteria	Cyanobacteriota	Cyanophyceae	Oscillatoriales	Microcoleaceae	Trichodesmium	Trichodesmium erythraeum
Bacteria	Cyanobacteriota	Cyanophyceae	Acaryochloridales	Thermosynechococcaceae	Thermosynechococcus	Thermosynechococcus sp.
Bacteria	Cyanobacteriota	Cyanophyceae	Oculatellales	Oculatellaceae	Thermoleptolyngbya	Thermoleptolyngbya sichuanensis
Bacteria	Cyanobacteriota	Cyanophyceae	Oculatellales	Oculatellaceae	Thermoleptolyngbya	Thermoleptolyngbya oregonensis
Bacteria	Cyanobacteriota	Cyanophyceae	Nostocales	Tolypothrichaceae	Tolypothrix	Tolypothrix sp.
Bacteria	Cyanobacteriota	Cyanophyceae	Oculatellales	Oculatellaceae	Thermocoleostomius	Thermocoleostomius sinensis
Bacteria	Cyanobacteriota	Cyanophyceae	Acaryochloridales	Thermosynechococcaceae	Thermosynechococcus	Thermosynechococcus sichuanensis
Bacteria	Cyanobacteriota	Cyanophyceae	Leptolyngbyales	Trichocoleusaceae	Trichothermofontia	Trichothermofontia sichuanensis
Bacteria	Cyanobacteriota	Cyanophyceae	Nostocales	Tolypothrichaceae	Tolypothrix	Tolypothrix tenuis
Bacteria	Cyanobacteriota	Cyanophyceae	Thermostichales	Thermostichaceae	Thermostichus	Thermostichus vulcanus
Bacteria	Cyanobacteriota	Cyanophyceae	Synechococcales	Coelosphaeriaceae	Woronichinia	Woronichinia naegeliana

Gene_Organisms: apcB

**Kingdom**	**Phylum**	**Class**	**Order**	**Family**	**Gunus**	**Species**
Bacteria	Cyanobacteriota	Cyanophyceae	Acaryochloridales	Acaryochloridaceae	Acaryochloris	unclassified Acaryochloris species
Bacteria	Cyanobacteriota	Cyanophyceae	Nostocales	Nostocaceae	Anabaena	Anabaena cylindrica
Bacteria	Cyanobacteriota	Cyanophyceae	Nostocales	Nodulariaceae	Anabaenopsis	Anabaenopsis elenkinii
Bacteria	Cyanobacteriota	Cyanophyceae	Nostocales	Aphanizomenonaceae	Aphanizomenon	Aphanizomenon flos-aquae
Bacteria	Cyanobacteriota	Cyanophyceae	Nostocales	Fortieaceae	Aulosira	Aulosira laxa
Bacteria	Cyanobacteriota	Cyanophyceae	Acaryochloridales	Acaryochloridaceae	Acaryochloris	Acaryochloris marina
Bacteria	Cyanobacteriota	Cyanophyceae	Nostocales	Nostocaceae	Nostoc	Nostoc sp.
Bacteria	Cyanobacteriota	Cyanophyceae	Nostocales	Nostocaceae	Anabaena	Anabaena sp.
Bacteria	Cyanobacteriota	Cyanophyceae	Oscillatoriales	Microcoleaceae	Arthrospira	Arthrospira platensis
Bacteria	Cyanobacteriota	Cyanophyceae	Nostocales	Nostocaceae	Trichormus	Trichormus variabilis
Bacteria	Cyanobacteriota	Cyanophyceae	Nostocales	Scytonemataceae	Brasilonema	Brasilonema octagenarum
Bacteria	Cyanobacteriota	Cyanophyceae	Nostocales	Scytonemataceae	Brasilonema	Brasilonema sennae
Bacteria	Cyanobacteriota	Cyanophyceae	Nostocales	Calotrichaceae	Calothrix	Calothrix sp.
Bacteria	Cyanobacteriota	Cyanophyceae	Chroococcales	Geminocystaceae	Cyanobacterium	Cyanobacterium aponinum
Bacteria	Cyanobacteriota	Cyanophyceae	Nostocales	Aphanizomenonaceae	Cylindrospermopsis	Cylindrospermopsis curvispora
Bacteria	Cyanobacteriota	Cyanophyceae	Gomontiellales	Gomontiellaceae	Crinalium	Crinalium epipsammum
Bacteria	Cyanobacteriota	Cyanophyceae	Synechococcales	Prochlorococcaceae	Cyanobium	Cyanobium gracile
Bacteria	Cyanobacteriota	Cyanophyceae	Chroococcales	Chroococcaceae	Chondrocystis	unclassified Chondrocystis species
Bacteria	Cyanobacteriota	Cyanophyceae	Gomontiellales	Chamaesiphonaceae	Chamaesiphon	Chamaesiphon minutus
Bacteria	Cyanobacteriota	Cyanophyceae	Nostocales	Aphanizomenonaceae	Cylindrospermopsis	Cylindrospermopsis raciborskii
Bacteria	Cyanobacteriota	Cyanophyceae	Nostocales	Nostocaceae	Cylindrospermum	Cylindrospermum stagnale
Bacteria	Cyanobacteriota	Cyanophyceae	Chroococcales	Geminocystaceae	Cyanobacterium	Cyanobacterium stanieri
Bacteria	Cyanobacteriota	Cyanophyceae	Chroococcidiopsidales	Chroococcidiopsidaceae	Chroococcidiopsis	Chroococcidiopsis thermalis
Bacteria	Cyanobacteriota	Cyanophyceae	Chroococcales	Aphanothecaceae	Crocosphaera	Crocosphaera watsonii
Bacteria	Cyanobacteriota	Cyanophyceae	Synechococcales	Synechococcaceae	Synechococcus	Synechococcus sp.
Bacteria	Cyanobacteriota	Cyanophyceae	Chroococcales	Aphanothecaceae	Gloeothece	Gloeothece citriformis
Bacteria	Cyanobacteriota	Cyanophyceae	Chroococcales	Aphanothecaceae	Rippkaea	Rippkaea orientalis
Bacteria	Cyanobacteriota	Cyanophyceae	Synechococcales	Prochlorococcaceae	Cyanobium	Cyanobium sp.
Bacteria	Cyanobacteriota	Cyanophyceae	Chroococcales	Aphanothecaceae	Gloeothece	Gloeothece verrucosa
Bacteria	Cyanobacteriota	Cyanophyceae	Chroococcales	Geminocystaceae	Cyanobacterium	Cyanobacterium sp.
Bacteria	Cyanobacteriota	Cyanophyceae	Gomontiellales	Cyanothecaceae	Cyanothece	Cyanothece sp.
Bacteria	Cyanobacteriota	Cyanophyceae	Chroococcales	Aphanothecaceae	Crocosphaera	Crocosphaera subtropica
Bacteria	Cyanobacteriota	Cyanophyceae	Nostocales	Aphanizomenonaceae	Dolichospermum	Dolichospermum compactum
Bacteria	Cyanobacteriota	Cyanophyceae	Nostocales	Aphanizomenonaceae	Dolichospermum	Dolichospermum flos-aquae
Bacteria	Cyanobacteriota	Cyanophyceae	Nostocales	Aphanizomenonaceae	Dolichospermum	Dolichospermum heterosporum
Bacteria	Cyanobacteriota	Cyanophyceae	Nostocales	Aphanizomenonaceae	Dolichospermum	Dolichospermum sp.

Bacteria	Cyanobacteriota	Cyanophyceae	Synechococcales	Synechococcaceae	Dactylococcopsis	Dactylococcopsis salina
Bacteria	Cyanobacteriota	Cyanophyceae	Chroococcales	Halothecacae	Euhalothece	Euhalothece natronophila
Bacteria	Cyanobacteriota	Cyanophyceae	Nostocales	Hapalosiphonaceae	Fischerella	Fischerella sp.
Bacteria	Cyanobacteriota	Cyanophyceae	Chroococcales	Chroococcaceae	Gloeocapsopsis	Gloeocapsopsis dulcis
Bacteria	Cyanobacteriota	Cyanophyceae	Chroococcales	Geminocystaceae	Geminocystis	Geminocystis sp.
Bacteria	Cyanobacteriota	Cyanophyceae	Geitlerinematales	Geitlerinemataceae	Geitlerinema	Geitlerinema sp.
Bacteria	Cyanobacteriota	Cyanophyceae	Gloeobacterales	Gloeobacteraceae	Gloeobacter	Gloeobacter kilaueensis
Bacteria	Cyanobacteriota	Cyanophyceae	Chroococcales	Chroococcaceae	Gloeocapsa	unclassified Gloeocapsa species
Bacteria	Cyanobacteriota	Cyanophyceae	Gloeomargaritales	Gloeomargaritaceae	Gloeomargarita	Gloeomargarita lithophora
Bacteria	Cyanobacteriota	Cyanophyceae	Gloeobacterales	Gloeobacteraceae	Gloeobacter	Gloeobacter morelensis
Eukaryota	Rhodophyta	Bangiophyceae	Galdieriales	Galdieriaceae	Galdieria	Galdieria sulphuraria
Bacteria	Cyanobacteriota	Cyanophyceae	Gloeobacterales	Gloeobacteraceae	Gloeobacter	Gloeobacter violaceus
Bacteria	Cyanobacteriota	Cyanophyceae	Chroococcales	Halothecacae	Halothece	Halothece sp.
Bacteria	Cyanobacteriota	Cyanophyceae	Nostocales	Nodulariaceae	Halotia	Halotia branconii
Bacteria	Cyanobacteriota	Cyanophyceae	Nodosilineales	Nodosilineaceae	Halomicronema	Halomicronema hongdechloris
Bacteria	Cyanobacteriota	Cyanophyceae	Leptolyngbyales	Leptolyngbyaceae	Kovacikia	Kovacikia minuta
Bacteria	Cyanobacteriota	Cyanophyceae	Leptolyngbyales	Leptolyngbyaceae	Leptolyngbya	Leptolyngbya boryana
Bacteria	Cyanobacteriota	Cyanophyceae	Leptolyngbyales	Leptolyngbyaceae	Leptolyngbya	Leptolyngbya sp.
Bacteria	Cyanobacteriota	Cyanophyceae	Oscillatoriales	Sirenicapillariaceae	Limnospira	Limnospira fusiformis
Bacteria	Cyanobacteriota	Cyanophyceae	Oscillatoriales	Sirenicapillariaceae	Limnospira	Limnospira indica
Bacteria	Cyanobacteriota	Cyanophyceae	Leptolyngbyales	Leptolyngbyaceae	Leptodesmis	Leptodesmis sichuanensis
Bacteria	Cyanobacteriota	Cyanophyceae	Leptolyngbyales	Leptolyngbyaceae	Leptothermofonsia	Leptothermofonsia sichuanensis
Bacteria	Cyanobacteriota	Cyanophyceae	Chroococcales	Microcystaceae	Microcystis	Microcystis aeruginosa
Bacteria	Cyanobacteriota	Cyanophyceae	Nostocales	Rivulariaceae	Microchaete	Microchaete diplosiphon
Bacteria	Cyanobacteriota	Cyanophyceae	Coleofasciculales	Coleofasciculaceae	Allocoleopsis	Allocoleopsis franciscana
Bacteria	Cyanobacteriota	Cyanophyceae	Chroococcales	Microcystaceae	Microcystis	Microcystis sp.
Bacteria	Cyanobacteriota	Cyanophyceae	Chroococcales	Microcystaceae	Microcystis	Microcystis panniformis
Bacteria	Cyanobacteriota	Cyanophyceae	Oscillatoriales	Oscillatoriaceae	Moorena	Moorena producens
Bacteria	Cyanobacteriota	Cyanophyceae	Oscillatoriales	Microcoleaceae	Microcoleus	Microcoleus vaginatus
Bacteria	Cyanobacteriota	Cyanophyceae	Chroococcales	Microcystaceae	Microcystis	Microcystis viridis
Bacteria	Cyanobacteriota	Cyanophyceae	Nostocales	unclassified Nostocales family	unclassified Nostocales genus	Nostocales cyanobacterium
Bacteria	Cyanobacteriota	Cyanophyceae	Nostocales	Nostocaceae	Nostoc	Nostoc carneum
Bacteria	Cyanobacteriota	Cyanophyceae	Nostocales	Nostocaceae	Nostoc	Nostoc edaphicum
Bacteria	Cyanobacteriota	Cyanophyceae	Nostocales	Nostocaceae	Nostoc	Nostoc flagelliforme
Bacteria	Cyanobacteriota	Cyanophyceae	Nostocales	Nostocaceae	Nostoc	Nostoc linckia
Bacteria	Cyanobacteriota	Cyanophyceae	Nostocales	Nostocaceae	Nostoc	Nostoc punctiforme
Bacteria	Cyanobacteriota	Cyanophyceae	Nostocales	Nostocaceae	Nostoc	Nostoc piscinale
Bacteria	Cyanobacteriota	Cyanophyceae	Nostocales	Nostocaceae	Nostoc	Nostoc sphaeroides

Bacteria	Cyanobacteriota	Cyanophyceae	Nostocales	Nodulariaceae	Nodularia	Nodularia sphaerocarpa
Bacteria	Cyanobacteriota	Cyanophyceae	Oscillatoriales	Oscillatoriaceae	Oscillatoria	Oscillatoria acuminata
Bacteria	Cyanobacteriota	Cyanophyceae	Nostocales	Aphanizomenonaceae	Okeanomitos	Okeanomitos corallinicola
Bacteria	Cyanobacteriota	Cyanophyceae	Oscillatoriales	Oscillatoriaceae	Oscillatoria	Oscillatoria nigro-viridis
Bacteria	Cyanobacteriota	Cyanophyceae	Oscillatoriales	Oscillatoriaceae	Oxynema	Oxynema aestuarii
Bacteria	Cyanobacteriota	Cyanophyceae	Oscillatoriales	Microcoleaceae	Planktothrix	Planktothrix agardhii
Bacteria	Cyanobacteriota	Cyanophyceae	Pseudanabaenales	Pseudanabaenaceae	Pseudanabaena	Pseudanabaena galeata
Bacteria	Cyanobacteriota	Cyanophyceae	Oscillatoriales	Oscillatoriaceae	Phormidium	Phormidium sp.
Bacteria	Cyanobacteriota	Cyanophyceae	Chroococcales	Geminocystaceae	Picosynechococcus	unclassified Picosynechococcus species
Bacteria	Cyanobacteriota	Cyanophyceae	Pleurocapsales	Hyellaceae	Pleurocapsa	Pleurocapsa sp.
Bacteria	Cyanobacteriota	Cyanophyceae	Oscillatoriales	Microcoleaceae	Planktothrix	Planktothrix pseudagardhii
Bacteria	Cyanobacteriota	Cyanophyceae	Oscillatoriales	Microcoleaceae	Planktothrix	Planktothrix rubescens
Bacteria	Cyanobacteriota	Cyanophyceae	Pseudanabaenales	Pseudanabaenaceae	Pseudanabaena	Pseudanabaena sp.
Bacteria	Cyanobacteriota	Cyanophyceae	Oscillatoriales	Oscillatoriaceae	Phormidium	Phormidium yuhuli
Bacteria	Cyanobacteriota	Cyanophyceae	Nostocales	Aphanizomenonaceae	Raphidiopsis	Raphidiopsis curvata
Bacteria	Cyanobacteriota	Cyanophyceae	Nostocales	Nostocaceae	Richelia	Richelia sinica
Bacteria	Cyanobacteriota	Cyanophyceae	Pleurocapsales	Dermocarpellaceae	Stanieria	Stanieria cyanosphaera
Bacteria	Cyanobacteriota	Cyanophyceae	Nostocales	Scytonemataceae	Scytonema	Scytonema sp.
Bacteria	Cyanobacteriota	Cyanophyceae	Nostocales	Aphanizomenonaceae	Sphaerospermopsis	Sphaerospermopsis kisseleviana
Bacteria	Cyanobacteriota	Cyanophyceae	Acaryochloridales	Thermosynechococcaceae	Parathermosynechococcus	Parathermosynechococcus lividus
Bacteria	Cyanobacteriota	Cyanophyceae	Pleurocapsales	Dermocarpellaceae	Stanieria	Stanieria sp.
Bacteria	Cyanobacteriota	Cyanophyceae	Nostocales	Aphanizomenonaceae	Sphaerospermopsis	Sphaerospermopsis torques-reginae
Bacteria	Cyanobacteriota	Cyanophyceae	Synechococcales	Synechococcaceae	Synechococcus	Synechococcus elongatus
Bacteria	Cyanobacteriota	Cyanophyceae	Synechococcales	Merismopediaceae	Synechocystis	Synechocystis sp.
Bacteria	Cyanobacteriota	Cyanophyceae	Synechococcales	Prochlorococcaceae	Parasynechococcus	Parasynechococcus marenigrum
Bacteria	Cyanobacteriota	Cyanophyceae	Acaryochloridales	Thermosynechococcaceae	Thermosynechococcus	Thermosynechococcus vestitus
Bacteria	Cyanobacteriota	Cyanophyceae	Oscillatoriales	Microcoleaceae	Trichodesmium	Trichodesmium erythraeum
Bacteria	Cyanobacteriota	Cyanophyceae	Acaryochloridales	Thermosynechococcaceae	Thermosynechococcus	Thermosynechococcus sp.
Bacteria	Cyanobacteriota	Cyanophyceae	Oculatellales	Oculatellaceae	Thermoleptolyngbya	Thermoleptolyngbya sichuanensis
Bacteria	Cyanobacteriota	Cyanophyceae	Oculatellales	Oculatellaceae	Thermoleptolyngbya	Thermoleptolyngbya oregonensis
Bacteria	Cyanobacteriota	Cyanophyceae	Nostocales	Tolypothrichaceae	Tolypothrix	Tolypothrix sp.
Bacteria	Cyanobacteriota	Cyanophyceae	Oculatellales	Oculatellaceae	Thermocoleostomius	Thermocoleostomius sinensis
Bacteria	Cyanobacteriota	Cyanophyceae	Acaryochloridales	Thermosynechococcaceae	Thermosynechococcus	Thermosynechococcus sichuanensis
Bacteria	Cyanobacteriota	Cyanophyceae	Leptolyngbyales	Trichocoleusaceae	Trichothermofontia	Trichothermofontia sichuanensis
Bacteria	Cyanobacteriota	Cyanophyceae	Nostocales	Tolypothrichaceae	Tolypothrix	Tolypothrix tenuis
Bacteria	Cyanobacteriota	Cyanophyceae	Thermostichales	Thermostichaceae	Thermostichus	Thermostichus vulcanus
Bacteria	Cyanobacteriota	Cyanophyceae	Synechococcales	Coelosphaeriaceae	Woronichinia	Woronichinia naegeliana

Gene_Organisms: cpcA

Kingdom	Phylum	Class	Order	Family	Gunus	Species
Bacteria	Cyanobacteriota	Cyanophyceae	Nostocales	Nostocaceae	Anabaena	Anabaena cylindrica
Bacteria	Cyanobacteriota	Cyanophyceae	Nostocales	Nodulariaceae	Anabaenopsis	Anabaenopsis elenkinii
Bacteria	Cyanobacteriota	Cyanophyceae	Nostocales	Aphanizomenonaceae	Aphanizomenon	Aphanizomenon flos-aquae
Bacteria	Cyanobacteriota	Cyanophyceae	Nostocales	Fortieaceae	Aulosira	Aulosira laxa
Bacteria	Cyanobacteriota	Cyanophyceae	Acaryochloridales	Acaryochloridaceae	Acaryochloris	Acaryochloris marina
Bacteria	Cyanobacteriota	Cyanophyceae	Nostocales	Nostocaceae	Nostoc	Nostoc sp.
Bacteria	Cyanobacteriota	Cyanophyceae	Nostocales	Nostocaceae	Anabaena	Anabaena sp.
Bacteria	Cyanobacteriota	Cyanophyceae	Oscillatoriales	Microcoleaceae	Arthrospira	Arthrospira platensis
Bacteria	Cyanobacteriota	Cyanophyceae	Nostocales	Nostocaceae	Trichormus	Trichormus variabilis
Bacteria	Cyanobacteriota	Cyanophyceae	Nostocales	Scytonemataceae	Brasilonema	Brasilonema octagenarum
Bacteria	Cyanobacteriota	Cyanophyceae	Nostocales	Scytonemataceae	Brasilonema	Brasilonema sennae
Bacteria	Cyanobacteriota	Cyanophyceae	Nostocales	Calotrichaceae	Calothrix	Calothrix sp.
Bacteria	Cyanobacteriota	Cyanophyceae	Chroococcales	Geminocystaceae	Cyanobacterium	Cyanobacterium aponinum
Eukaryota	Rhodophyta	Florideophyceae	Gigartinales	Gigartinaceae	Chondrus	Chondrus crispus
Bacteria	Cyanobacteriota	Cyanophyceae	Nostocales	Aphanizomenonaceae	Cylindrospermopsis	Cylindrospermopsis curvispora
Bacteria	Cyanobacteriota	Cyanophyceae	Gomontiellales	Gomontiellaceae	Crinalium	Crinalium epipsammum
Bacteria	Cyanobacteriota	Cyanophyceae	Synechococcales	Prochlorococcaceae	Cyanobium	Cyanobium gracile
Bacteria	Cyanobacteriota	Cyanophyceae	Chroococcales	Chroococcaceae	Chondrocystis	unclassified Chondrocystis species
Eukaryota	Rhodophyta	Bangiophyceae	Cyanidiales	Cyanidiaceae	Cyanidioschyzon	Cyanidioschyzon merolae
Bacteria	Cyanobacteriota	Cyanophyceae	Gomontiellales	Chamaesiphonaceae	Chamaesiphon	Chamaesiphon minutus
Bacteria	Cyanobacteriota	Cyanophyceae	Nostocales	Aphanizomenonaceae	Cylindrospermopsis	Cylindrospermopsis raciborskii
Bacteria	Cyanobacteriota	Cyanophyceae	Nostocales	Nostocaceae	Cylindrospermum	Cylindrospermum stagnale
Bacteria	Cyanobacteriota	Cyanophyceae	Chroococcales	Geminocystaceae	Cyanobacterium	Cyanobacterium stanieri
Bacteria	Cyanobacteriota	Cyanophyceae	Chroococcidiopsidales	Chroococcidiopsidaceae	Chroococcidiopsis	Chroococcidiopsis thermalis
Bacteria	Cyanobacteriota	Cyanophyceae	Chroococcales	Aphanothecaceae	Crocosphaera	Crocosphaera watsonii
Bacteria	Cyanobacteriota	Cyanophyceae	Synechococcales	Synechococcaceae	Synechococcus	Synechococcus sp.
Bacteria	Cyanobacteriota	Cyanophyceae	Chroococcales	Aphanothecaceae	Gloeothece	Gloeothece citriformis
Bacteria	Cyanobacteriota	Cyanophyceae	Chroococcales	Aphanothecaceae	Rippkaea	Rippkaea orientalis
Bacteria	Cyanobacteriota	Cyanophyceae	Synechococcales	Prochlorococcaceae	Cyanobium	Cyanobium sp.
Bacteria	Cyanobacteriota	Cyanophyceae	Chroococcales	Aphanothecaceae	Gloeothece	Gloeothece verrucosa
Bacteria	Cyanobacteriota	Cyanophyceae	Chroococcales	Geminocystaceae	Cyanobacterium	Cyanobacterium sp.
Bacteria	Cyanobacteriota	Cyanophyceae	Gomontiellales	Cyanothecaceae	Cyanothece	Cyanothece sp.
Bacteria	Cyanobacteriota	Cyanophyceae	Chroococcales	Aphanothecaceae	Crocosphaera	Crocosphaera subtropica
Bacteria	Cyanobacteriota	Cyanophyceae	Nostocales	Aphanizomenonaceae	Dolichospermum	Dolichospermum compactum
Bacteria	Cyanobacteriota	Cyanophyceae	Nostocales	Aphanizomenonaceae	Dolichospermum	Dolichospermum flos-aquae
Bacteria	Cyanobacteriota	Cyanophyceae	Nostocales	Aphanizomenonaceae	Dolichospermum	Dolichospermum heterosporum

Bacteria	Cyanobacteriota	Cyanophyceae	Nostocales	Aphanizomenonaceae	Dolichospermum	Dolichospermum sp.
Bacteria	Cyanobacteriota	Cyanophyceae	Synechococcales	Synechococcaceae	Dactylococcopsis	Dactylococcopsis salina
Bacteria	Cyanobacteriota	Cyanophyceae	Chroococcales	Halothecacae	Euhalothece	Euhalothece natronophila
Bacteria	Cyanobacteriota	Cyanophyceae	Nostocales	Hapalosiphonaceae	Fischerella	Fischerella sp.
Bacteria	Cyanobacteriota	Cyanophyceae	Chroococcales	Chroococcaceae	Gloeocapsopsis	Gloeocapsopsis dulcis
Bacteria	Cyanobacteriota	Cyanophyceae	Chroococcales	Geminocystaceae	Geminocystis	Geminocystis sp.
Bacteria	Cyanobacteriota	Cyanophyceae	Geitlerinematales	Geitlerinemataceae	Geitlerinema	Geitlerinema sp.
Bacteria	Cyanobacteriota	Cyanophyceae	Gloeobacterales	Gloeobacteraceae	Gloeobacter	Gloeobacter kilaueensis
Bacteria	Cyanobacteriota	Cyanophyceae	Chroococcales	Chroococcaceae	Gloeocapsa	unclassified Gloeocapsa species
Bacteria	Cyanobacteriota	Cyanophyceae	Gloeomargaritales	Gloeomargaritaceae	Gloeomargarita	Gloeomargarita lithophora
Bacteria	Cyanobacteriota	Cyanophyceae	Gloeobacterales	Gloeobacteraceae	Gloeobacter	Gloeobacter morelensis
Eukaryota	Rhodophyta	Bangiophyceae	Galdieriales	Galdieriaceae	Galdieria	Galdieria sulphuraria
Bacteria	Cyanobacteriota	Cyanophyceae	Gloeobacterales	Gloeobacteraceae	Gloeobacter	Gloeobacter violaceus
Bacteria	Cyanobacteriota	Cyanophyceae	Chroococcales	Halothecacae	Halothece	Halothece sp.
Bacteria	Cyanobacteriota	Cyanophyceae	Nostocales	Nodulariaceae	Halotia	Halotia branconii
Bacteria	Cyanobacteriota	Cyanophyceae	Nodosilineales	Nodosilineaceae	Halomicronema	Halomicronema hongdechloris
Bacteria	Cyanobacteriota	Cyanophyceae	Leptolyngbyales	Leptolyngbyaceae	Kovacikia	Kovacikia minuta
Bacteria	Cyanobacteriota	Cyanophyceae	Leptolyngbyales	Leptolyngbyaceae	Leptolyngbya	Leptolyngbya boryana
Bacteria	Cyanobacteriota	Cyanophyceae	Leptolyngbyales	Leptolyngbyaceae	Leptolyngbya	Leptolyngbya sp.
Bacteria	Cyanobacteriota	Cyanophyceae	Oscillatoriales	Sirenicapillariaceae	Limnospira	Limnospira fusiformis
Bacteria	Cyanobacteriota	Cyanophyceae	Oscillatoriales	Sirenicapillariaceae	Limnospira	Limnospira indica
Bacteria	Cyanobacteriota	Cyanophyceae	Leptolyngbyales	Leptolyngbyaceae	Leptothermofonsia	Leptothermofonsia sichuanensis
Bacteria	Cyanobacteriota	Cyanophyceae	Chroococcales	Microcystaceae	Microcystis	Microcystis aeruginosa
Bacteria	Cyanobacteriota	Cyanophyceae	Nostocales	Rivulariaceae	Microchaete	Microchaete diplosiphon
Bacteria	Cyanobacteriota	Cyanophyceae	Coleofasciculales	Coleofasciculaceae	Allocoleopsis	Allocoleopsis franciscana
Bacteria	Cyanobacteriota	Cyanophyceae	Chroococcales	Microcystaceae	Microcystis	Microcystis sp.
Bacteria	Cyanobacteriota	Cyanophyceae	Chroococcales	Microcystaceae	Microcystis	Microcystis panniformis
Bacteria	Cyanobacteriota	Cyanophyceae	Oscillatoriales	Oscillatoriaceae	Moorena	Moorena producens
Bacteria	Cyanobacteriota	Cyanophyceae	Oscillatoriales	Microcoleaceae	Microcoleus	Microcoleus vaginatus
Bacteria	Cyanobacteriota	Cyanophyceae	Chroococcales	Microcystaceae	Microcystis	Microcystis viridis
Bacteria	Cyanobacteriota	Cyanophyceae	Nostocales	unclassified Nostocales family	unclassified Nostocales genus	Nostocales cyanobacterium
Bacteria	Cyanobacteriota	Cyanophyceae	Nostocales	Nostocaceae	Nostoc	Nostoc carneum
Bacteria	Cyanobacteriota	Cyanophyceae	Nostocales	Nostocaceae	Nostoc	Nostoc edaphicum
Bacteria	Cyanobacteriota	Cyanophyceae	Nostocales	Nostocaceae	Nostoc	Nostoc flagelliforme
Bacteria	Cyanobacteriota	Cyanophyceae	Nostocales	Nostocaceae	Nostoc	Nostoc linckia
Bacteria	Cyanobacteriota	Cyanophyceae	Nostocales	Nostocaceae	Nostoc	Nostoc punctiforme
Bacteria	Cyanobacteriota	Cyanophyceae	Nostocales	Nostocaceae	Nostoc	Nostoc piscinale
Bacteria	Cyanobacteriota	Cyanophyceae	Nostocales	Nostocaceae	Nostoc	Nostoc sphaeroides

Bacteria	Cyanobacteriota	Cyanophyceae	Nostocales	Nodulariaceae	Nodularia	Nodularia spumigena
Bacteria	Cyanobacteriota	Cyanophyceae	Nostocales	Nodulariaceae	Nodularia	Nodularia sphaerocarpa
Bacteria	Cyanobacteriota	Cyanophyceae	Oscillatoriales	Oscillatoriaceae	Oscillatoria	Oscillatoria acuminata
Bacteria	Cyanobacteriota	Cyanophyceae	Nostocales	Aphanizomenonaceae	Okeanomitos	Okeanomitos corallinicola
Bacteria	Cyanobacteriota	Cyanophyceae	Oscillatoriales	Oscillatoriaceae	Oscillatoria	Oscillatoria nigro-viridis
Bacteria	Cyanobacteriota	Cyanophyceae	Oscillatoriales	Oscillatoriaceae	Oxynema	Oxynema aestuarii
Bacteria	Cyanobacteriota	Cyanophyceae	Oscillatoriales	Microcoleaceae	Planktothrix	Planktothrix agardhii
Bacteria	Cyanobacteriota	Cyanophyceae	Pseudanabaenales	Pseudanabaenaceae	Pseudanabaena	Pseudanabaena galeata
Bacteria	Cyanobacteriota	Cyanophyceae	Oscillatoriales	Oscillatoriaceae	Phormidium	Phormidium sp.
Bacteria	Cyanobacteriota	Cyanophyceae	Chroococcales	Geminocystaceae	Picosynechococcus	unclassified Picosynechococcus species
Bacteria	Cyanobacteriota	Cyanophyceae	Pleurocapsales	Hyellaceae	Pleurocapsa	Pleurocapsa sp.
Bacteria	Cyanobacteriota	Cyanophyceae	Oscillatoriales	Microcoleaceae	Planktothrix	Planktothrix pseudagardhii
Bacteria	Cyanobacteriota	Cyanophyceae	Oscillatoriales	Microcoleaceae	Planktothrix	Planktothrix rubescens
Bacteria	Cyanobacteriota	Cyanophyceae	Pseudanabaenales	Pseudanabaenaceae	Pseudanabaena	Pseudanabaena sp.
Bacteria	Cyanobacteriota	Cyanophyceae	Oscillatoriales	Oscillatoriaceae	Phormidium	Phormidium yuhuli
Bacteria	Cyanobacteriota	Cyanophyceae	Nostocales	Aphanizomenonaceae	Raphidiopsis	Raphidiopsis curvata
Bacteria	Cyanobacteriota	Cyanophyceae	Nostocales	Nostocaceae	Richelia	Richelia sinica
Bacteria	Cyanobacteriota	Cyanophyceae	Pleurocapsales	Dermocarpellaceae	Stanieria	Stanieria cyanosphaera
Bacteria	Cyanobacteriota	Cyanophyceae	Nostocales	Scytonemataceae	Scytonema	Scytonema sp.
Bacteria	Cyanobacteriota	Cyanophyceae	Nostocales	Aphanizomenonaceae	Sphaerospermopsis	Sphaerospermopsis kisseleviana
Bacteria	Cyanobacteriota	Cyanophyceae	Acaryochloridales	Thermosynechococcaceae	Parathermosynechococcus	Parathermosynechococcus lividus
Bacteria	Cyanobacteriota	Cyanophyceae	Pleurocapsales	Dermocarpellaceae	Stanieria	Stanieria sp.
Bacteria	Cyanobacteriota	Cyanophyceae	Nostocales	Aphanizomenonaceae	Sphaerospermopsis	Sphaerospermopsis torques-reginae
Bacteria	Cyanobacteriota	Cyanophyceae	Synechococcales	Synechococcaceae	Synechococcus	Synechococcus elongatus
Bacteria	Cyanobacteriota	Cyanophyceae	Synechococcales	Merismopediaceae	Synechocystis	Synechocystis sp.
Bacteria	Cyanobacteriota	Cyanophyceae	Synechococcales	Prochlorococcaceae	Parasynechococcus	Parasynechococcus marenigrum
Bacteria	Cyanobacteriota	Cyanophyceae	Acaryochloridales	Thermosynechococcaceae	Thermosynechococcus	Thermosynechococcus vestitus
Bacteria	Cyanobacteriota	Cyanophyceae	Oscillatoriales	Microcoleaceae	Trichodesmium	Trichodesmium erythraeum
Bacteria	Cyanobacteriota	Cyanophyceae	Acaryochloridales	Thermosynechococcaceae	Thermosynechococcus	Thermosynechococcus sp.
Bacteria	Cyanobacteriota	Cyanophyceae	Oculatellales	Oculatellaceae	Thermoleptolyngbya	Thermoleptolyngbya sichuanensis
Bacteria	Cyanobacteriota	Cyanophyceae	Oculatellales	Oculatellaceae	Thermoleptolyngbya	Thermoleptolyngbya oregonensis
Bacteria	Cyanobacteriota	Cyanophyceae	Nostocales	Tolypothrichaceae	Tolypothrix	Tolypothrix sp.
Bacteria	Cyanobacteriota	Cyanophyceae	Oculatellales	Oculatellaceae	Thermocoleostomius	Thermocoleostomius sinensis
Bacteria	Cyanobacteriota	Cyanophyceae	Acaryochloridales	Thermosynechococcaceae	Thermosynechococcus	Thermosynechococcus sichuanensis
Bacteria	Cyanobacteriota	Cyanophyceae	Leptolyngbyales	Trichocoleusaceae	Trichothermofontia	Trichothermofontia sichuanensis
Bacteria	Cyanobacteriota	Cyanophyceae	Nostocales	Tolypothrichaceae	Tolypothrix	Tolypothrix tenuis
Bacteria	Cyanobacteriota	Cyanophyceae	Thermostichales	Thermostichaceae	Thermostichus	Thermostichus vulcanus
Bacteria	Cyanobacteriota	Cyanophyceae	Synechococcales	Coelosphaeriaceae	Woronichinia	Woronichinia naegeliana

Gene_Organisms: cpcB

Kingdom	Phylum	Class	Order	Family	Gunus	Species
Bacteria	Cyanobacteriota	Cyanophyceae	Nostocales	Nostocaceae	Anabaena	Anabaena cylindrica
Bacteria	Cyanobacteriota	Cyanophyceae	Nostocales	Nodulariaceae	Anabaenopsis	Anabaenopsis elenkinii
Bacteria	Cyanobacteriota	Cyanophyceae	Nostocales	Aphanizomenonaceae	Aphanizomenon	Aphanizomenon flos-aquae
Bacteria	Cyanobacteriota	Cyanophyceae	Nostocales	Fortieaceae	Aulosira	Aulosira laxa
Bacteria	Cyanobacteriota	Cyanophyceae	Acaryochloridales	Acaryochloridaceae	Acaryochloris	Acaryochloris marina
Bacteria	Cyanobacteriota	Cyanophyceae	Nostocales	Nostocaceae	Nostoc	Nostoc sp.
Bacteria	Cyanobacteriota	Cyanophyceae	Nostocales	Nostocaceae	Anabaena	Anabaena sp.
Bacteria	Cyanobacteriota	Cyanophyceae	Oscillatoriales	Microcoleaceae	Arthrospira	Arthrospira platensis
Bacteria	Cyanobacteriota	Cyanophyceae	Nostocales	Nostocaceae	Trichormus	Trichormus variabilis
Bacteria	Cyanobacteriota	Cyanophyceae	Nostocales	Scytonemataceae	Brasilonema	Brasilonema octagenarum
Bacteria	Cyanobacteriota	Cyanophyceae	Nostocales	Scytonemataceae	Brasilonema	Brasilonema sennae
Bacteria	Cyanobacteriota	Cyanophyceae	Nostocales	Calotrichaceae	Calothrix	Calothrix sp.
Bacteria	Cyanobacteriota	Cyanophyceae	Chroococcales	Geminocystaceae	Cyanobacterium	Cyanobacterium aponinum
Eukaryota	Rhodophyta	Florideophyceae	Gigartinales	Gigartinaceae	Chondrus	Chondrus crispus
Bacteria	Cyanobacteriota	Cyanophyceae	Nostocales	Aphanizomenonaceae	Cylindrospermopsis	Cylindrospermopsis curvispora
Bacteria	Cyanobacteriota	Cyanophyceae	Gomontiellales	Gomontiellaceae	Crinalium	Crinalium epipsammum
Bacteria	Cyanobacteriota	Cyanophyceae	Synechococcales	Prochlorococcaceae	Cyanobium	Cyanobium gracile
Bacteria	Cyanobacteriota	Cyanophyceae	Chroococcales	Chroococcaceae	Chondrocystis	unclassified Chondrocystis species
Eukaryota	Rhodophyta	Bangiophyceae	Cyanidiales	Cyanidiaceae	Cyanidioschyzon	Cyanidioschyzon merolae
Bacteria	Cyanobacteriota	Cyanophyceae	Gomontiellales	Chamaesiphonaceae	Chamaesiphon	Chamaesiphon minutus
Bacteria	Cyanobacteriota	Cyanophyceae	Nostocales	Aphanizomenonaceae	Cylindrospermopsis	Cylindrospermopsis raciborskii
Bacteria	Cyanobacteriota	Cyanophyceae	Nostocales	Nostocaceae	Cylindrospermum	Cylindrospermum stagnale
Bacteria	Cyanobacteriota	Cyanophyceae	Chroococcales	Geminocystaceae	Cyanobacterium	Cyanobacterium stanieri
Bacteria	Cyanobacteriota	Cyanophyceae	Chroococcidiopsidales	Chroococcidiopsidaceae	Chroococcidiopsis	Chroococcidiopsis thermalis
Bacteria	Cyanobacteriota	Cyanophyceae	Chroococcales	Aphanothecaceae	Crocosphaera	Crocosphaera watsonii
Bacteria	Cyanobacteriota	Cyanophyceae	Synechococcales	Synechococcaceae	Synechococcus	Synechococcus sp.
Bacteria	Cyanobacteriota	Cyanophyceae	Chroococcales	Aphanothecaceae	Gloeothece	Gloeothece citriformis
Bacteria	Cyanobacteriota	Cyanophyceae	Chroococcales	Aphanothecaceae	Rippkaea	Rippkaea orientalis
Bacteria	Cyanobacteriota	Cyanophyceae	Synechococcales	Prochlorococcaceae	Cyanobium	Cyanobium sp.
Bacteria	Cyanobacteriota	Cyanophyceae	Chroococcales	Aphanothecaceae	Gloeothece	Gloeothece verrucosa
Bacteria	Cyanobacteriota	Cyanophyceae	Chroococcales	Geminocystaceae	Cyanobacterium	Cyanobacterium sp.
Bacteria	Cyanobacteriota	Cyanophyceae	Gomontiellales	Cyanothecaceae	Cyanothece	Cyanothece sp.
Bacteria	Cyanobacteriota	Cyanophyceae	Chroococcales	Aphanothecaceae	Crocosphaera	Crocosphaera subtropica
Bacteria	Cyanobacteriota	Cyanophyceae	Nostocales	Aphanizomenonaceae	Dolichospermum	Dolichospermum compactum
Bacteria	Cyanobacteriota	Cyanophyceae	Nostocales	Aphanizomenonaceae	Dolichospermum	Dolichospermum flos-aquae
Bacteria	Cyanobacteriota	Cyanophyceae	Nostocales	Aphanizomenonaceae	Dolichospermum	Dolichospermum heterosporum

Bacteria	Cyanobacteriota	Cyanophyceae	Nostocales	Aphanizomenonaceae	Dolichospermum	Dolichospermum sp.
Bacteria	Cyanobacteriota	Cyanophyceae	Synechococcales	Synechococcaceae	Dactylococcopsis	Dactylococcopsis salina
Bacteria	Cyanobacteriota	Cyanophyceae	Chroococcales	Halothecacae	Euhalothece	Euhalothece natronophila
Bacteria	Cyanobacteriota	Cyanophyceae	Nostocales	Hapalosiphonaceae	Fischerella	Fischerella sp.
Bacteria	Gemmatimonadota	Gemmatimonadia	Gemmatimonadales	Gemmatimonadaceae	Gemmatirosa	Gemmatirosa kalamazoonensis
Bacteria	Cyanobacteriota	Cyanophyceae	Chroococcales	Chroococcaceae	Gloeocapsopsis	Gloeocapsopsis dulcis
Bacteria	Cyanobacteriota	Cyanophyceae	Chroococcales	Geminocystaceae	Geminocystis	Geminocystis sp.
Bacteria	Cyanobacteriota	Cyanophyceae	Geitlerinematales	Geitlerinemataceae	Geitlerinema	Geitlerinema sp.
Bacteria	Cyanobacteriota	Cyanophyceae	Gloeobacterales	Gloeobacteraceae	Gloeobacter	Gloeobacter kilaueensis
Bacteria	Cyanobacteriota	Cyanophyceae	Chroococcales	Chroococcaceae	Gloeocapsa	unclassified Gloeocapsa species
Bacteria	Cyanobacteriota	Cyanophyceae	Gloeomargaritales	Gloeomargaritaceae	Gloeomargarita	Gloeomargarita lithophora
Bacteria	Cyanobacteriota	Cyanophyceae	Gloeobacterales	Gloeobacteraceae	Gloeobacter	Gloeobacter morelensis
Bacteria	Cyanobacteriota	Cyanophyceae	Gloeobacterales	Gloeobacteraceae	Gloeobacter	Gloeobacter violaceus
Bacteria	Cyanobacteriota	Cyanophyceae	Chroococcales	Halothecacae	Halothece	Halothece sp.
Bacteria	Cyanobacteriota	Cyanophyceae	Nostocales	Nodulariaceae	Halotia	Halotia branconii
Bacteria	Cyanobacteriota	Cyanophyceae	Nodosilineales	Nodosilineaceae	Halomicronema	Halomicronema hongdechloris
Bacteria	Cyanobacteriota	Cyanophyceae	Leptolyngbyales	Leptolyngbyaceae	Kovacikia	Kovacikia minuta
Bacteria	Cyanobacteriota	Cyanophyceae	Leptolyngbyales	Leptolyngbyaceae	Leptolyngbya	Leptolyngbya boryana
Bacteria	Cyanobacteriota	Cyanophyceae	Leptolyngbyales	Leptolyngbyaceae	Leptolyngbya	Leptolyngbya sp.
Bacteria	Cyanobacteriota	Cyanophyceae	Oscillatoriales	Sirenicapillariaceae	Limnospira	Limnospira fusiformis
Bacteria	Cyanobacteriota	Cyanophyceae	Oscillatoriales	Sirenicapillariaceae	Limnospira	Limnospira indica
Bacteria	Cyanobacteriota	Cyanophyceae	Leptolyngbyales	Leptolyngbyaceae	Leptothermofonsia	Leptothermofonsia sichuanensis
Bacteria	Cyanobacteriota	Cyanophyceae	Chroococcales	Microcystaceae	Microcystis	Microcystis aeruginosa
Bacteria	Cyanobacteriota	Cyanophyceae	Nostocales	Rivulariaceae	Microchaete	Microchaete diplosiphon
Bacteria	Cyanobacteriota	Cyanophyceae	Coleofasciculales	Coleofasciculaceae	Allocoleopsis	Allocoleopsis franciscana
Bacteria	Cyanobacteriota	Cyanophyceae	Chroococcales	Microcystaceae	Microcystis	Microcystis sp.
Bacteria	Cyanobacteriota	Cyanophyceae	Chroococcales	Microcystaceae	Microcystis	Microcystis panniformis
Bacteria	Cyanobacteriota	Cyanophyceae	Oscillatoriales	Oscillatoriaceae	Moorena	Moorena producens
Bacteria	Cyanobacteriota	Cyanophyceae	Oscillatoriales	Microcoleaceae	Microcoleus	Microcoleus vaginatus
Bacteria	Cyanobacteriota	Cyanophyceae	Chroococcales	Microcystaceae	Microcystis	Microcystis viridis
Bacteria	Cyanobacteriota	Cyanophyceae	Nostocales	unclassified Nostocales family	unclassified Nostocales genus	Nostocales cyanobacterium
Bacteria	Cyanobacteriota	Cyanophyceae	Nostocales	Nostocaceae	Nostoc	Nostoc carneum
Bacteria	Cyanobacteriota	Cyanophyceae	Nostocales	Nostocaceae	Nostoc	Nostoc edaphicum
Bacteria	Cyanobacteriota	Cyanophyceae	Nostocales	Nostocaceae	Nostoc	Nostoc flagelliforme
Bacteria	Cyanobacteriota	Cyanophyceae	Nostocales	Nostocaceae	Nostoc	Nostoc linckia
Bacteria	Cyanobacteriota	Cyanophyceae	Nostocales	Nostocaceae	Nostoc	Nostoc punctiforme
Bacteria	Cyanobacteriota	Cyanophyceae	Nostocales	Nostocaceae	Nostoc	Nostoc piscinale
Bacteria	Cyanobacteriota	Cyanophyceae	Nostocales	Nostocaceae	Nostoc	Nostoc sphaeroides

Bacteria	Cyanobacteriota	Cyanophyceae	Nostocales	Nodulariaceae	Nodularia	Nodularia spumigena
Bacteria	Cyanobacteriota	Cyanophyceae	Nostocales	Nodulariaceae	Nodularia	Nodularia sphaerocarpa
Bacteria	Cyanobacteriota	Cyanophyceae	Oscillatoriales	Oscillatoriaceae	Oscillatoria	Oscillatoria acuminata
Bacteria	Cyanobacteriota	Cyanophyceae	Nostocales	Aphanizomenonaceae	Okeanomitos	Okeanomitos corallinicola
Bacteria	Cyanobacteriota	Cyanophyceae	Oscillatoriales	Oscillatoriaceae	Oscillatoria	Oscillatoria nigro-viridis
Bacteria	Cyanobacteriota	Cyanophyceae	Oscillatoriales	Oscillatoriaceae	Oxynema	Oxynema aestuarii
Bacteria	Cyanobacteriota	Cyanophyceae	Oscillatoriales	Microcoleaceae	Planktothrix	Planktothrix agardhii
Bacteria	Cyanobacteriota	Cyanophyceae	Pseudanabaenales	Pseudanabaenaceae	Pseudanabaena	Pseudanabaena galeata
Bacteria	Cyanobacteriota	Cyanophyceae	Oscillatoriales	Oscillatoriaceae	Phormidium	Phormidium sp.
Bacteria	Cyanobacteriota	Cyanophyceae	Chroococcales	Geminocystaceae	Picosynechococcus	unclassified Picosynechococcus species
Bacteria	Cyanobacteriota	Cyanophyceae	Pleurocapsales	Hyellaceae	Pleurocapsa	Pleurocapsa sp.
Bacteria	Cyanobacteriota	Cyanophyceae	Oscillatoriales	Microcoleaceae	Planktothrix	Planktothrix pseudagardhii
Bacteria	Cyanobacteriota	Cyanophyceae	Oscillatoriales	Microcoleaceae	Planktothrix	Planktothrix rubescens
Bacteria	Cyanobacteriota	Cyanophyceae	Pseudanabaenales	Pseudanabaenaceae	Pseudanabaena	Pseudanabaena sp.
Bacteria	Cyanobacteriota	Cyanophyceae	Oscillatoriales	Oscillatoriaceae	Phormidium	Phormidium yuhuli
Bacteria	Cyanobacteriota	Cyanophyceae	Nostocales	Aphanizomenonaceae	Raphidiopsis	Raphidiopsis curvata
Bacteria	Cyanobacteriota	Cyanophyceae	Nostocales	Nostocaceae	Richelia	Richelia sinica
Bacteria	Cyanobacteriota	Cyanophyceae	Pleurocapsales	Dermocarpellaceae	Stanieria	Stanieria cyanosphaera
Bacteria	Cyanobacteriota	Cyanophyceae	Nostocales	Scytonemataceae	Scytonema	Scytonema sp.
Bacteria	Cyanobacteriota	Cyanophyceae	Nostocales	Aphanizomenonaceae	Sphaerospermopsis	Sphaerospermopsis kisseleviana
Bacteria	Cyanobacteriota	Cyanophyceae	Acaryochloridales	Thermosynechococcaceae	Parathermosynechococcus	Parathermosynechococcus lividus
Bacteria	Cyanobacteriota	Cyanophyceae	Pleurocapsales	Dermocarpellaceae	Stanieria	Stanieria sp.
Bacteria	Cyanobacteriota	Cyanophyceae	Nostocales	Aphanizomenonaceae	Sphaerospermopsis	Sphaerospermopsis torques-reginae
Bacteria	Cyanobacteriota	Cyanophyceae	Synechococcales	Synechococcaceae	Synechococcus	Synechococcus elongatus
Bacteria	Cyanobacteriota	Cyanophyceae	Synechococcales	Merismopediaceae	Synechocystis	Synechocystis sp.
Bacteria	Cyanobacteriota	Cyanophyceae	Synechococcales	Prochlorococcaceae	Parasynechococcus	Parasynechococcus marenigrum
Bacteria	Cyanobacteriota	Cyanophyceae	Acaryochloridales	Thermosynechococcaceae	Thermosynechococcus	Thermosynechococcus vestitus
Bacteria	Cyanobacteriota	Cyanophyceae	Oscillatoriales	Microcoleaceae	Trichodesmium	Trichodesmium erythraeum
Bacteria	Cyanobacteriota	Cyanophyceae	Acaryochloridales	Thermosynechococcaceae	Thermosynechococcus	Thermosynechococcus sp.
Bacteria	Cyanobacteriota	Cyanophyceae	Oculatellales	Oculatellaceae	Thermoleptolyngbya	Thermoleptolyngbya sichuanensis
Bacteria	Cyanobacteriota	Cyanophyceae	Oculatellales	Oculatellaceae	Thermoleptolyngbya	Thermoleptolyngbya oregonensis
Bacteria	Cyanobacteriota	Cyanophyceae	Nostocales	Tolypothrichaceae	Tolypothrix	Tolypothrix sp.
Bacteria	Cyanobacteriota	Cyanophyceae	Oculatellales	Oculatellaceae	Thermocoleostomius	Thermocoleostomius sinensis
Bacteria	Cyanobacteriota	Cyanophyceae	Acaryochloridales	Thermosynechococcaceae	Thermosynechococcus	Thermosynechococcus sichuanensis
Bacteria	Cyanobacteriota	Cyanophyceae	Leptolyngbyales	Trichocoleusaceae	Trichothermofontia	Trichothermofontia sichuanensis
Bacteria	Cyanobacteriota	Cyanophyceae	Nostocales	Tolypothrichaceae	Tolypothrix	Tolypothrix tenuis
Bacteria	Cyanobacteriota	Cyanophyceae	Thermostichales	Thermostichaceae	Thermostichus	Thermostichus vulcanus
Bacteria	Cyanobacteriota	Cyanophyceae	Synechococcales	Coelosphaeriaceae	Woronichinia	Woronichinia naegeliana

Gene_Organisms: psaA

**Kingdom**	**Phylum**	**Class**	**Order**	**Family**	**Gunus**	**Species**
Bacteria	Cyanobacteriota	Cyanophyceae	Acaryochloridales	Acaryochloridaceae	Acaryochloris	unclassified Acaryochloris species
Bacteria	Cyanobacteriota	Cyanophyceae	Nostocales	Nostocaceae	Anabaena	Anabaena cylindrica
Bacteria	Cyanobacteriota	Cyanophyceae	Nostocales	Nodulariaceae	Anabaenopsis	Anabaenopsis elenkinii
Bacteria	Cyanobacteriota	Cyanophyceae	Nostocales	Aphanizomenonaceae	Aphanizomenon	Aphanizomenon flos-aquae
Bacteria	Cyanobacteriota	Cyanophyceae	Nostocales	Fortieaceae	Aulosira	Aulosira laxa
Bacteria	Cyanobacteriota	Cyanophyceae	Acaryochloridales	Acaryochloridaceae	Acaryochloris	Acaryochloris marina
Bacteria	Cyanobacteriota	Cyanophyceae	Nostocales	Nostocaceae	Nostoc	Nostoc sp.
Bacteria	Cyanobacteriota	Cyanophyceae	Nostocales	Nostocaceae	Anabaena	Anabaena sp.
Bacteria	Cyanobacteriota	Cyanophyceae	Oscillatoriales	Microcoleaceae	Arthrospira	Arthrospira platensis
Bacteria	Cyanobacteriota	Cyanophyceae	Nostocales	Nostocaceae	Trichormus	Trichormus variabilis
Bacteria	Cyanobacteriota	Cyanophyceae	Nostocales	Scytonemataceae	Brasilonema	Brasilonema octagenarum
Bacteria	Cyanobacteriota	Cyanophyceae	Nostocales	Scytonemataceae	Brasilonema	Brasilonema sennae
Bacteria	Cyanobacteriota	Cyanophyceae	Nostocales	Calotrichaceae	Calothrix	Calothrix sp.
Bacteria	Cyanobacteriota	Cyanophyceae	Chroococcales	Geminocystaceae	Cyanobacterium	Cyanobacterium aponinum
Bacteria	Cyanobacteriota	Cyanophyceae	Nostocales	Aphanizomenonaceae	Cylindrospermopsis	Cylindrospermopsis curvispora
Bacteria	Cyanobacteriota	Cyanophyceae	Gomontiellales	Gomontiellaceae	Crinalium	Crinalium epipsammum
Bacteria	Cyanobacteriota	Cyanophyceae	Synechococcales	Prochlorococcaceae	Cyanobium	Cyanobium gracile
Bacteria	Cyanobacteriota	Cyanophyceae	Chroococcales	Chroococcaceae	Chondrocystis	unclassified Chondrocystis species
Bacteria	Cyanobacteriota	Cyanophyceae	Gomontiellales	Chamaesiphonaceae	Chamaesiphon	Chamaesiphon minutus
Bacteria	Cyanobacteriota	Cyanophyceae	Nostocales	Aphanizomenonaceae	Cylindrospermopsis	Cylindrospermopsis raciborskii
Bacteria	Cyanobacteriota	Cyanophyceae	Nostocales	Nostocaceae	Cylindrospermum	Cylindrospermum stagnale
Bacteria	Cyanobacteriota	Cyanophyceae	Chroococcales	Geminocystaceae	Cyanobacterium	Cyanobacterium stanieri
Bacteria	Cyanobacteriota	Cyanophyceae	Chroococcidiopsidales	Chroococcidiopsidaceae	Chroococcidiopsis	Chroococcidiopsis thermalis
Bacteria	Cyanobacteriota	Cyanophyceae	Chroococcales	Aphanothecaceae	Crocosphaera	Crocosphaera watsonii
Bacteria	Cyanobacteriota	Cyanophyceae	Synechococcales	Synechococcaceae	Synechococcus	Synechococcus sp.
Bacteria	Cyanobacteriota	Cyanophyceae	Chroococcales	Aphanothecaceae	Gloeothece	Gloeothece citriformis
Bacteria	Cyanobacteriota	Cyanophyceae	Chroococcales	Aphanothecaceae	Rippkaea	Rippkaea orientalis
Bacteria	Cyanobacteriota	Cyanophyceae	Synechococcales	Prochlorococcaceae	Cyanobium	Cyanobium sp.
Bacteria	Cyanobacteriota	Cyanophyceae	Chroococcales	Aphanothecaceae	Gloeothece	Gloeothece verrucosa
Bacteria	Cyanobacteriota	Cyanophyceae	Chroococcales	Geminocystaceae	Cyanobacterium	Cyanobacterium sp.
Bacteria	Cyanobacteriota	Cyanophyceae	Gomontiellales	Cyanothecaceae	Cyanothece	Cyanothece sp.
Bacteria	Cyanobacteriota	Cyanophyceae	Chroococcales	Aphanothecaceae	Crocosphaera	Crocosphaera subtropica
Bacteria	Cyanobacteriota	Cyanophyceae	Chroococcales	Aphanothecaceae	Candidatus Atelocyanobacterium	unclassified Candidatus Atelocyanobacterium species
Bacteria	Cyanobacteriota	Cyanophyceae	Nostocales	Aphanizomenonaceae	Dolichospermum	Dolichospermum compactum
Bacteria	Cyanobacteriota	Cyanophyceae	Nostocales	Aphanizomenonaceae	Dolichospermum	Dolichospermum flos-aquae
Bacteria	Cyanobacteriota	Cyanophyceae	Nostocales	Aphanizomenonaceae	Dolichospermum	Dolichospermum heterosporum

Bacteria	Cyanobacteriota	Cyanophyceae	Nostocales	Aphanizomenonaceae	Dolichospermum	Dolichospermum sp.
Bacteria	Cyanobacteriota	Cyanophyceae	Synechococcales	Synechococcaceae	Dactylococcopsis	Dactylococcopsis salina
Bacteria	Cyanobacteriota	Cyanophyceae	Chroococcales	Halothecacae	Euhalothece	Euhalothece natronophila
Bacteria	Cyanobacteriota	Cyanophyceae	Nostocales	Hapalosiphonaceae	Fischerella	Fischerella sp.
Bacteria	Cyanobacteriota	Cyanophyceae	Chroococcales	Chroococcaceae	Gloeocapsopsis	Gloeocapsopsis dulcis
Bacteria	Cyanobacteriota	Cyanophyceae	Chroococcales	Geminocystaceae	Geminocystis	Geminocystis sp.
Bacteria	Cyanobacteriota	Cyanophyceae	Geitlerinematales	Geitlerinemataceae	Geitlerinema	Geitlerinema sp.
Bacteria	Cyanobacteriota	Cyanophyceae	Gloeobacterales	Gloeobacteraceae	Gloeobacter	Gloeobacter kilaueensis
Bacteria	Cyanobacteriota	Cyanophyceae	Chroococcales	Chroococcaceae	Gloeocapsa	unclassified Gloeocapsa species
Bacteria	Cyanobacteriota	Cyanophyceae	Gloeomargaritales	Gloeomargaritaceae	Gloeomargarita	Gloeomargarita lithophora
Bacteria	Cyanobacteriota	Cyanophyceae	Gloeobacterales	Gloeobacteraceae	Gloeobacter	Gloeobacter morelensis
Bacteria	Cyanobacteriota	Cyanophyceae	Gloeobacterales	Gloeobacteraceae	Gloeobacter	Gloeobacter violaceus
Bacteria	Cyanobacteriota	Cyanophyceae	Chroococcales	Halothecacae	Halothece	Halothece sp.
Bacteria	Cyanobacteriota	Cyanophyceae	Nostocales	Nodulariaceae	Halotia	Halotia branconii
Bacteria	Cyanobacteriota	Cyanophyceae	Nodosilineales	Nodosilineaceae	Halomicronema	Halomicronema hongdechloris
Bacteria	Cyanobacteriota	Cyanophyceae	Leptolyngbyales	Leptolyngbyaceae	Kovacikia	Kovacikia minuta
Bacteria	Cyanobacteriota	Cyanophyceae	Leptolyngbyales	Leptolyngbyaceae	Leptolyngbya	Leptolyngbya boryana
Bacteria	Cyanobacteriota	Cyanophyceae	Leptolyngbyales	Leptolyngbyaceae	Leptolyngbya	Leptolyngbya sp.
Bacteria	Cyanobacteriota	Cyanophyceae	Oscillatoriales	Sirenicapillariaceae	Limnospira	Limnospira fusiformis
Bacteria	Cyanobacteriota	Cyanophyceae	Oscillatoriales	Sirenicapillariaceae	Limnospira	Limnospira indica
Bacteria	Cyanobacteriota	Cyanophyceae	Leptolyngbyales	Leptolyngbyaceae	Leptodesmis	Leptodesmis sichuanensis
Bacteria	Cyanobacteriota	Cyanophyceae	Leptolyngbyales	Leptolyngbyaceae	Leptothermofonsia	Leptothermofonsia sichuanensis
Bacteria	Cyanobacteriota	Cyanophyceae	Chroococcales	Microcystaceae	Microcystis	Microcystis aeruginosa
Bacteria	Cyanobacteriota	Cyanophyceae	Nostocales	Rivulariaceae	Microchaete	Microchaete diplosiphon
Bacteria	Cyanobacteriota	Cyanophyceae	Coleofasciculales	Coleofasciculaceae	Allocoleopsis	Allocoleopsis franciscana
Bacteria	Cyanobacteriota	Cyanophyceae	Chroococcales	Microcystaceae	Microcystis	Microcystis sp.
Bacteria	Cyanobacteriota	Cyanophyceae	Chroococcales	Microcystaceae	Microcystis	Microcystis panniformis
Bacteria	Cyanobacteriota	Cyanophyceae	Oscillatoriales	Oscillatoriaceae	Moorena	Moorena producens
Bacteria	Cyanobacteriota	Cyanophyceae	Oscillatoriales	Microcoleaceae	Microcoleus	Microcoleus vaginatus
Bacteria	Cyanobacteriota	Cyanophyceae	Chroococcales	Microcystaceae	Microcystis	Microcystis viridis
Bacteria	Cyanobacteriota	Cyanophyceae	Nostocales	unclassified Nostocales family	unclassified Nostocales genus	Nostocales cyanobacterium
Bacteria	Cyanobacteriota	Cyanophyceae	Nostocales	Nostocaceae	Nostoc	Nostoc carneum
Bacteria	Cyanobacteriota	Cyanophyceae	Nostocales	Nostocaceae	Nostoc	Nostoc edaphicum
Bacteria	Cyanobacteriota	Cyanophyceae	Nostocales	Nostocaceae	Nostoc	Nostoc flagelliforme
Bacteria	Cyanobacteriota	Cyanophyceae	Nostocales	Nostocaceae	Nostoc	Nostoc linckia
Bacteria	Cyanobacteriota	Cyanophyceae	Nostocales	Nostocaceae	Nostoc	Nostoc punctiforme
Bacteria	Cyanobacteriota	Cyanophyceae	Nostocales	Nostocaceae	Nostoc	Nostoc piscinale
Bacteria	Cyanobacteriota	Cyanophyceae	Nostocales	Nostocaceae	Nostoc	Nostoc sphaeroides

Bacteria	Cyanobacteriota	Cyanophyceae	Nostocales	Nodulariaceae	Nodularia	Nodularia spumigena
Bacteria	Cyanobacteriota	Cyanophyceae	Nostocales	Nodulariaceae	Nodularia	Nodularia sphaerocarpa
Bacteria	Cyanobacteriota	Cyanophyceae	Oscillatoriales	Oscillatoriaceae	Oscillatoria	Oscillatoria acuminata
Bacteria	Cyanobacteriota	Cyanophyceae	Nostocales	Aphanizomenonaceae	Okeanomitos	Okeanomitos corallinicola
Bacteria	Cyanobacteriota	Cyanophyceae	Oscillatoriales	Oscillatoriaceae	Oscillatoria	Oscillatoria nigro-viridis
Bacteria	Cyanobacteriota	Cyanophyceae	Oscillatoriales	Oscillatoriaceae	Oxynema	Oxynema aestuarii
Bacteria	Cyanobacteriota	Cyanophyceae	Oscillatoriales	Microcoleaceae	Planktothrix	Planktothrix agardhii
Bacteria	Cyanobacteriota	Cyanophyceae	Pseudanabaenales	Pseudanabaenaceae	Pseudanabaena	Pseudanabaena galeata
Bacteria	Cyanobacteriota	Cyanophyceae	Oscillatoriales	Oscillatoriaceae	Phormidium	Phormidium sp.
Bacteria	Cyanobacteriota	Cyanophyceae	Chroococcales	Geminocystaceae	Picosynechococcus	unclassified Picosynechococcus species
Bacteria	Cyanobacteriota	Cyanophyceae	Pleurocapsales	Hyellaceae	Pleurocapsa	Pleurocapsa sp.
Bacteria	Cyanobacteriota	Cyanophyceae	Synechococcales	Prochlorococcaceae	Prochlorococcus	Prochlorococcus marinus
Bacteria	Cyanobacteriota	Cyanophyceae	Oscillatoriales	Microcoleaceae	Planktothrix	Planktothrix pseudagardhii
Bacteria	Cyanobacteriota	Cyanophyceae	Synechococcales	Prochlorococcaceae	Prochlorococcus	Prochlorococcus sp.
Bacteria	Cyanobacteriota	Cyanophyceae	Oscillatoriales	Microcoleaceae	Planktothrix	Planktothrix rubescens
Bacteria	Cyanobacteriota	Cyanophyceae	Pseudanabaenales	Pseudanabaenaceae	Pseudanabaena	Pseudanabaena sp.
Bacteria	Cyanobacteriota	Cyanophyceae	Oscillatoriales	Oscillatoriaceae	Phormidium	Phormidium yuhuli
Bacteria	Cyanobacteriota	Cyanophyceae	Nostocales	Aphanizomenonaceae	Raphidiopsis	Raphidiopsis curvata
Bacteria	Cyanobacteriota	Cyanophyceae	Nostocales	Nostocaceae	Richelia	Richelia sinica
Bacteria	Cyanobacteriota	Cyanophyceae	Pleurocapsales	Dermocarpellaceae	Stanieria	Stanieria cyanosphaera
Bacteria	Cyanobacteriota	Cyanophyceae	Nostocales	Scytonemataceae	Scytonema	Scytonema sp.
Bacteria	Cyanobacteriota	Cyanophyceae	Nostocales	Aphanizomenonaceae	Sphaerospermopsis	Sphaerospermopsis kisseleviana
Bacteria	Cyanobacteriota	Cyanophyceae	Acaryochloridales	Thermosynechococcaceae	Parathermosynechococcus	Parathermosynechococcus lividus
Bacteria	Cyanobacteriota	Cyanophyceae	Pleurocapsales	Dermocarpellaceae	Stanieria	Stanieria sp.
Bacteria	Cyanobacteriota	Cyanophyceae	Nostocales	Aphanizomenonaceae	Sphaerospermopsis	Sphaerospermopsis torques-reginae
Bacteria	Cyanobacteriota	Cyanophyceae	Synechococcales	Synechococcaceae	Synechococcus	Synechococcus elongatus
Bacteria	Cyanobacteriota	Cyanophyceae	Synechococcales	Merismopediaceae	Synechocystis	Synechocystis sp.
Bacteria	Cyanobacteriota	Cyanophyceae	Synechococcales	Prochlorococcaceae	Parasynechococcus	Parasynechococcus marenigrum
Bacteria	Cyanobacteriota	Cyanophyceae	Acaryochloridales	Thermosynechococcaceae	Thermosynechococcus	Thermosynechococcus vestitus
Bacteria	Cyanobacteriota	Cyanophyceae	Oscillatoriales	Microcoleaceae	Trichodesmium	Trichodesmium erythraeum
Bacteria	Cyanobacteriota	Cyanophyceae	Acaryochloridales	Thermosynechococcaceae	Thermosynechococcus	Thermosynechococcus sp.
Bacteria	Cyanobacteriota	Cyanophyceae	Oculatellales	Oculatellaceae	Thermoleptolyngbya	Thermoleptolyngbya sichuanensis
Bacteria	Cyanobacteriota	Cyanophyceae	Oculatellales	Oculatellaceae	Thermoleptolyngbya	Thermoleptolyngbya oregonensis
Bacteria	Cyanobacteriota	Cyanophyceae	Nostocales	Tolypothrichaceae	Tolypothrix	Tolypothrix sp.
Bacteria	Cyanobacteriota	Cyanophyceae	Oculatellales	Oculatellaceae	Thermocoleostomius	Thermocoleostomius sinensis
Bacteria	Cyanobacteriota	Cyanophyceae	Acaryochloridales	Thermosynechococcaceae	Thermosynechococcus	Thermosynechococcus sichuanensis
Bacteria	Cyanobacteriota	Cyanophyceae	Leptolyngbyales	Trichocoleusaceae	Trichothermofontia	Trichothermofontia sichuanensis
Bacteria	Cyanobacteriota	Cyanophyceae	Nostocales	Tolypothrichaceae	Tolypothrix	Tolypothrix tenuis

Bacteria	Cyanobacteriota	Cyanophyceae	Thermostichales	Thermostichaceae	Thermostichus	Thermostichus vulcanus
Bacteria	Cyanobacteriota	Cyanophyceae	Synechococcales	Coelosphaeriaceae	Woronichinia	Woronichinia naegeliana
Eukaryota	unclassified Eukaryota	Pelagophyceae	Pelagomonadales	Pelagomonadaceae	Aureococcus	Aureococcus anophagefferens
Eukaryota	Streptophyta	Magnoliopsida	Fabales	Fabaceae	Arachis	Arachis duranensis
Eukaryota	Streptophyta	Magnoliopsida	Ericales	Actinidiaceae	Actinidia	Actinidia eriantha
Eukaryota	Streptophyta	Magnoliopsida	Fabales	Fabaceae	Arachis	Arachis hypogaea
Eukaryota	Streptophyta	Magnoliopsida	Fabales	Fabaceae	Arachis	Arachis ipaensis
Eukaryota	Streptophyta	Magnoliopsida	Brassicales	Brassicaceae	Arabidopsis	Arabidopsis lyrata
Eukaryota	Streptophyta	Magnoliopsida	Asparagales	Asparagaceae	Asparagus	Asparagus officinalis
Eukaryota	Streptophyta	Magnoliopsida	Lamiales	Acanthaceae	Andrographis	Andrographis paniculata
Eukaryota	Streptophyta	Magnoliopsida	Fabales	Fabaceae	Abrus	Abrus precatorius
Eukaryota	Chlorophyta	Trebouxiophyceae	Chlorellales	Chlorellaceae	Auxenochlorella	Auxenochlorella protothecoides
Eukaryota	Streptophyta	Magnoliopsida	Brassicales	Brassicaceae	Arabidopsis	Arabidopsis thaliana
Eukaryota	Streptophyta	Magnoliopsida	Amborellales	Amborellaceae	Amborella	Amborella trichopoda
Eukaryota	Streptophyta	Magnoliopsida	Caryophyllales	Amaranthaceae	Amaranthus	Amaranthus tricolor
Eukaryota	Streptophyta	Magnoliopsida	Poales	Poaceae	Brachypodium	Brachypodium distachyon
Eukaryota	Streptophyta	Magnoliopsida	Cucurbitales	Cucurbitaceae	Benincasa	Benincasa hispida
Eukaryota	Streptophyta	Magnoliopsida	Brassicales	Brassicaceae	Brassica	Brassica napus
Eukaryota	Streptophyta	Magnoliopsida	Brassicales	Brassicaceae	Brassica	Brassica rapa
Eukaryota	Streptophyta	Magnoliopsida	Fabales	Fabaceae	Cicer	Cicer arietinum
Eukaryota	Streptophyta	Magnoliopsida	Solanales	Solanaceae	Capsicum	Capsicum annuum
Eukaryota	Streptophyta	Magnoliopsida	Fagales	Betulaceae	Corylus	Corylus avellana
Eukaryota	Streptophyta	Magnoliopsida	Fabales	Fabaceae	Cajanus	Cajanus cajan
Eukaryota	Rhodophyta	Florideophyceae	Gigartinales	Gigartinaceae	Chondrus	Chondrus crispus
Eukaryota	Streptophyta	Magnoliopsida	Sapindales	Rutaceae	Citrus	Citrus sinensis
Eukaryota	Streptophyta	Magnoliopsida	Cucurbitales	Cucurbitaceae	Cucurbita	Cucurbita maxima
Eukaryota	Rhodophyta	Bangiophyceae	Cyanidiales	Cyanidiaceae	Cyanidioschyzon	Cyanidioschyzon merolae
Eukaryota	Streptophyta	Magnoliopsida	Cucurbitales	Cucurbitaceae	Cucurbita	Cucurbita moschata
Eukaryota	Streptophyta	Magnoliopsida	Brassicales	Caricaceae	Carica	Carica papaya
Eukaryota	Streptophyta	Magnoliopsida	Caryophyllales	Chenopodiaceae	Chenopodium	Chenopodium quinoa
Eukaryota	Streptophyta	Magnoliopsida	Brassicales	Brassicaceae	Capsella	Capsella rubella
Eukaryota	Chlorophyta	Chlorophyceae	Chlamydomonadales	Chlamydomonadaceae	Chlamydomonas	Chlamydomonas reinhardtii
Eukaryota	Streptophyta	Magnoliopsida	Brassicales	Brassicaceae	Camelina	Camelina sativa
Eukaryota	Streptophyta	Magnoliopsida	Ericales	Theaceae	Camellia	Camellia sinensis
Eukaryota	Chlorophyta	Trebouxiophyceae	unclassified Trebouxiophyceae	unclassified Trebouxiophyceae family	Coccomyxa	Coccomyxa subellipsoidea
Eukaryota	Streptophyta	Magnoliopsida	Cucurbitales	Cucurbitaceae	Cucumis	Cucumis sativus
Eukaryota	Chlorophyta	Trebouxiophyceae	Chlorellales	Chlorellaceae	Chlorella	Chlorella variabilis

Eukaryota	Streptophyta	Magnoliopsida	Apiales	Apiaceae	Daucus	Daucus carota
Eukaryota	Streptophyta	Magnoliopsida	Asparagales	Orchidaceae	Dendrobium	Dendrobium catenatum
Eukaryota	Chordata	Mammalia	Rodentia	Heteromyidae	Dipodomys	Dipodomys spectabilis
Eukaryota	Streptophyta	Magnoliopsida	Malvales	Malvaceae	Durio	Durio zibethinus
Eukaryota	Streptophyta	Magnoliopsida	Asterales	Asteraceae	Erigeron	Erigeron canadensis
Eukaryota	Streptophyta	Magnoliopsida	Myrtales	Myrtaceae	Eucalyptus	Eucalyptus grandis
Eukaryota	Streptophyta	Magnoliopsida	Arecales	Arecaceae	Elaeis	Elaeis guineensis
Eukaryota	Streptophyta	Magnoliopsida	Brassicales	Brassicaceae	Eutrema	Eutrema salsugineum
Eukaryota	Streptophyta	Magnoliopsida	Rosales	Rosaceae	Fragaria	Fragaria vesca
Eukaryota	Streptophyta	Magnoliopsida	Malvales	Malvaceae	Gossypium	Gossypium arboreum
Eukaryota	Streptophyta	Magnoliopsida	Malvales	Malvaceae	Gossypium	Gossypium hirsutum
Eukaryota	Streptophyta	Magnoliopsida	Fabales	Fabaceae	Glycine	Glycine max
Eukaryota	Streptophyta	Magnoliopsida	Malvales	Malvaceae	Gossypium	Gossypium raimondii
Eukaryota	Streptophyta	Magnoliopsida	Fabales	Fabaceae	Glycine	Glycine soja
Eukaryota	Rhodophyta	Bangiophyceae	Galdieriales	Galdieriaceae	Galdieria	Galdieria sulphuraria
Eukaryota	Streptophyta	Magnoliopsida	Asterales	Asteraceae	Helianthus	Helianthus annuus
Eukaryota	Streptophyta	Magnoliopsida	Malpighiales	Euphorbiaceae	Hevea	Hevea brasiliensis
Eukaryota	Streptophyta	Magnoliopsida	Malvales	Malvaceae	Hibiscus	Hibiscus syriacus
Eukaryota	Streptophyta	Magnoliopsida	Solanales	Convolvulaceae	Ipomoea	Ipomoea nil
Eukaryota	Streptophyta	Magnoliopsida	Solanales	Convolvulaceae	Ipomoea	Ipomoea triloba
Eukaryota	Streptophyta	Magnoliopsida	Malpighiales	Euphorbiaceae	Jatropha	Jatropha curcas
Eukaryota	Streptophyta	Magnoliopsida	Fagales	Juglandaceae	Juglans	Juglans regia
Eukaryota	Streptophyta	Magnoliopsida	Solanales	Solanaceae	Lycium	Lycium barbarum
Eukaryota	Streptophyta	Magnoliopsida	Poales	Poaceae	Lolium	Lolium perenne
Eukaryota	Streptophyta	Magnoliopsida	Asterales	Asteraceae	Lactuca	Lactuca sativa
Eukaryota	Streptophyta	Magnoliopsida	Cucurbitales	Cucurbitaceae	Momordica	Momordica charantia
Eukaryota	Streptophyta	Magnoliopsida	Malpighiales	Euphorbiaceae	Mercurialis	Mercurialis annua
Eukaryota	Streptophyta	Magnoliopsida	Malpighiales	Euphorbiaceae	Manihot	Manihot esculenta
Eukaryota	Streptophyta	Magnoliopsida	Poales	Poaceae	Miscanthus	Miscanthus floridulus
Eukaryota	Streptophyta	Magnoliopsida	Sapindales	Anacardiaceae	Mangifera	Mangifera indica
Eukaryota	Streptophyta	Magnoliopsida	Proteales	Proteaceae	Macadamia	Macadamia integrifolia
Eukaryota	Streptophyta	Magnoliopsida	Rosales	Moraceae	Morus	Morus notabilis
Eukaryota	Streptophyta	Magnoliopsida	Magnoliales	Magnoliaceae	Magnolia	Magnolia sinica
Eukaryota	Streptophyta	Magnoliopsida	Rosales	Rosaceae	Malus	Malus sylvestris
Eukaryota	Streptophyta	Magnoliopsida	Fabales	Fabaceae	Medicago	Medicago truncatula
Eukaryota	Streptophyta	Magnoliopsida	Solanales	Solanaceae	Nicotiana	Nicotiana attenuata
Eukaryota	Streptophyta	Magnoliopsida	Nymphaeales	Nymphaeaceae	Nymphaea	Nymphaea colorata
Eukaryota	Streptophyta	Magnoliopsida	Proteales	Nelumbonaceae	Nelumbo	Nelumbo nucifera

Eukaryota	Streptophyta	Magnoliopsida	Solanales	Solanaceae	Nicotiana	Nicotiana sylvestris
Eukaryota	Streptophyta	Magnoliopsida	Solanales	Solanaceae	Nicotiana	Nicotiana tabacum
Eukaryota	Streptophyta	Magnoliopsida	Solanales	Solanaceae	Nicotiana	Nicotiana tomentosiformis
Eukaryota	Streptophyta	Magnoliopsida	Poales	Poaceae	Oryza	Oryza brachyantha
Eukaryota	Streptophyta	Magnoliopsida	Poales	Poaceae	Oryza	Oryza glaberrima
Eukaryota	Streptophyta	Magnoliopsida	Poales	Poaceae	Oryza	Oryza sativa
Eukaryota	Chlorophyta	Mamiellophyceae	Mamiellales	Bathycoccaceae	Ostreococcus	Ostreococcus tauri
Eukaryota	Streptophyta	Magnoliopsida	Malpighiales	Salicaceae	Populus	Populus alba
Eukaryota	Streptophyta	Magnoliopsida	Fabales	Fabaceae	Prosopis	Prosopis cineraria
Eukaryota	Streptophyta	Magnoliopsida	Arecales	Arecaceae	Phoenix	Phoenix dactylifera
Eukaryota	Streptophyta	Magnoliopsida	Rosales	Rosaceae	Prunus	Prunus dulcis
Eukaryota	Streptophyta	Magnoliopsida	Asparagales	Orchidaceae	Phalaenopsis	Phalaenopsis equestris
Eukaryota	Streptophyta	Magnoliopsida	Malpighiales	Salicaceae	Populus	Populus euphratica
Eukaryota	Streptophyta	Magnoliopsida	Rosales	Rosaceae	Prunus	Prunus mume
Eukaryota	Streptophyta	Magnoliopsida	Malpighiales	Salicaceae	Populus	Populus trichocarpa
Eukaryota	Streptophyta	Magnoliopsida	Rosales	Rosaceae	Prunus	Prunus persica
Eukaryota	Streptophyta	Bryopsida	Funariales	Funariaceae	Physcomitrium	Physcomitrium patens
Eukaryota	Streptophyta	Magnoliopsida	Fabales	Fabaceae	Pisum	Pisum sativum
Eukaryota	Streptophyta	Magnoliopsida	Ranunculales	Papaveraceae	Papaver	Papaver somniferum
Eukaryota	Streptophyta	Magnoliopsida	Poales	Poaceae	Panicum	Panicum virgatum
Eukaryota	Streptophyta	Magnoliopsida	Fabales	Fabaceae	Phaseolus	Phaseolus vulgaris
Eukaryota	Streptophyta	Magnoliopsida	Sapindales	Anacardiaceae	Pistacia	Pistacia vera
Eukaryota	Streptophyta	Magnoliopsida	Fabales	Quillajaceae	Quillaja	Quillaja saponaria
Eukaryota	Streptophyta	Magnoliopsida	Malpighiales	Euphorbiaceae	Ricinus	Ricinus communis
Eukaryota	Streptophyta	Magnoliopsida	Brassicales	Brassicaceae	Raphanus	Raphanus sativus
Eukaryota	Streptophyta	Magnoliopsida	Poales	Poaceae	Sorghum	Sorghum bicolor
Eukaryota	Streptophyta	Magnoliopsida	Solanales	Solanaceae	Solanum	Solanum dulcamara
Eukaryota	Streptophyta	Magnoliopsida	Lamiales	Lamiaceae	Salvia	Salvia hispanica
Eukaryota	Streptophyta	Magnoliopsida	Lamiales	Pedaliaceae	Sesamum	Sesamum indicum
Eukaryota	Streptophyta	Magnoliopsida	Poales	Poaceae	Setaria	Setaria italica
Eukaryota	Streptophyta	Magnoliopsida	Solanales	Solanaceae	Solanum	Solanum lycopersicum
Eukaryota	Streptophyta	Magnoliopsida	Lamiales	Lamiaceae	Salvia	Salvia miltiorrhiza
Eukaryota	Streptophyta	Lycopodiopsida	Selaginellales	Selaginellaceae	Selaginella	Selaginella moellendorffii
Eukaryota	Streptophyta	Magnoliopsida	Caryophyllales	Chenopodiaceae	Spinacia	Spinacia oleracea
Eukaryota	Streptophyta	Magnoliopsida	Solanales	Solanaceae	Solanum	Solanum tuberosum
Eukaryota	Streptophyta	Magnoliopsida	Solanales	Solanaceae	Solanum	Solanum pennellii
Eukaryota	Streptophyta	Magnoliopsida	Lamiales	Lamiaceae	Salvia	Salvia splendens
Eukaryota	Streptophyta	Magnoliopsida	Solanales	Solanaceae	Solanum	Solanum stenotomum

Eukaryota	Streptophyta	Magnoliopsida	Poales	Poaceae	Setaria	Setaria viridis
Eukaryota	Streptophyta	Magnoliopsida	Poales	Poaceae	Triticum	Triticum aestivum
Eukaryota	Streptophyta	Magnoliopsida	Malvales	Malvaceae	Theobroma	Theobroma cacao
Eukaryota	Streptophyta	Magnoliopsida	Brassicales	Cleomaceae	Tarenaya	Tarenaya hassleriana
Eukaryota	Streptophyta	Magnoliopsida	Fabales	Fabaceae	Trifolium	Trifolium pratense
Eukaryota	Streptophyta	Magnoliopsida	Proteales	Proteaceae	Telopea	Telopea speciosissima
Eukaryota	Streptophyta	Magnoliopsida	Poales	Poaceae	Triticum	Triticum urartu
Eukaryota	Streptophyta	Magnoliopsida	Fabales	Fabaceae	Vigna	Vigna angularis
Eukaryota	Streptophyta	Magnoliopsida	Vitales	Vitaceae	Vitis	Vitis riparia
Eukaryota	Streptophyta	Magnoliopsida	Fabales	Fabaceae	Vigna	Vigna unguiculata
Eukaryota	Streptophyta	Magnoliopsida	Vitales	Vitaceae	Vitis	Vitis vinifera
Eukaryota	Streptophyta	Magnoliopsida	Rosales	Rhamnaceae	Ziziphus	Ziziphus jujuba
Eukaryota	Streptophyta	Magnoliopsida	Poales	Poaceae	Zea	Zea mays
Eukaryota	Streptophyta	Magnoliopsida	Zingiberales	Zingiberaceae	Zingiber	Zingiber officinale

Gene_Organisms: psaB

**Kingdom**	**Phylum**	**Class**	**Order**	**Family**	**Gunus**	**Species**
Bacteria	Cyanobacteriota	Cyanophyceae	Acaryochloridales	Acaryochloridaceae	Acaryochloris	unclassified Acaryochloris species
Bacteria	Cyanobacteriota	Cyanophyceae	Nostocales	Nostocaceae	Anabaena	Anabaena cylindrica
Bacteria	Cyanobacteriota	Cyanophyceae	Nostocales	Nodulariaceae	Anabaenopsis	Anabaenopsis elenkinii
Bacteria	Cyanobacteriota	Cyanophyceae	Nostocales	Aphanizomenonaceae	Aphanizomenon	Aphanizomenon flos-aquae
Bacteria	Cyanobacteriota	Cyanophyceae	Nostocales	Fortieaceae	Aulosira	Aulosira laxa
Bacteria	Cyanobacteriota	Cyanophyceae	Acaryochloridales	Acaryochloridaceae	Acaryochloris	Acaryochloris marina
Bacteria	Cyanobacteriota	Cyanophyceae	Nostocales	Nostocaceae	Nostoc	Nostoc sp.
Bacteria	Cyanobacteriota	Cyanophyceae	Nostocales	Nostocaceae	Anabaena	Anabaena sp.
Bacteria	Cyanobacteriota	Cyanophyceae	Oscillatoriales	Microcoleaceae	Arthrospira	Arthrospira platensis
Bacteria	Cyanobacteriota	Cyanophyceae	Nostocales	Nostocaceae	Trichormus	Trichormus variabilis
Bacteria	Cyanobacteriota	Cyanophyceae	Nostocales	Scytonemataceae	Brasilonema	Brasilonema octagenarum
Bacteria	Cyanobacteriota	Cyanophyceae	Nostocales	Scytonemataceae	Brasilonema	Brasilonema sennae
Bacteria	Cyanobacteriota	Cyanophyceae	Nostocales	Calotrichaceae	Calothrix	Calothrix sp.
Bacteria	Cyanobacteriota	Cyanophyceae	Chroococcales	Geminocystaceae	Cyanobacterium	Cyanobacterium aponinum
Bacteria	Cyanobacteriota	Cyanophyceae	Nostocales	Aphanizomenonaceae	Cylindrospermopsis	Cylindrospermopsis curvispora
Bacteria	Cyanobacteriota	Cyanophyceae	Gomontiellales	Gomontiellaceae	Crinalium	Crinalium epipsammum
Bacteria	Cyanobacteriota	Cyanophyceae	Synechococcales	Prochlorococcaceae	Cyanobium	Cyanobium gracile
Bacteria	Cyanobacteriota	Cyanophyceae	Chroococcales	Chroococcaceae	Chondrocystis	unclassified Chondrocystis species
Bacteria	Cyanobacteriota	Cyanophyceae	Gomontiellales	Chamaesiphonaceae	Chamaesiphon	Chamaesiphon minutus
Bacteria	Cyanobacteriota	Cyanophyceae	Nostocales	Aphanizomenonaceae	Cylindrospermopsis	Cylindrospermopsis raciborskii
Bacteria	Cyanobacteriota	Cyanophyceae	Nostocales	Nostocaceae	Cylindrospermum	Cylindrospermum stagnale
Bacteria	Cyanobacteriota	Cyanophyceae	Chroococcales	Geminocystaceae	Cyanobacterium	Cyanobacterium stanieri
Bacteria	Cyanobacteriota	Cyanophyceae	Chroococcidiopsidales	Chroococcidiopsidaceae	Chroococcidiopsis	Chroococcidiopsis thermalis
Bacteria	Cyanobacteriota	Cyanophyceae	Chroococcales	Aphanothecaceae	Crocosphaera	Crocosphaera watsonii
Bacteria	Cyanobacteriota	Cyanophyceae	Synechococcales	Synechococcaceae	Synechococcus	Synechococcus sp.
Bacteria	Cyanobacteriota	Cyanophyceae	Chroococcales	Aphanothecaceae	Gloeothece	Gloeothece citriformis
Bacteria	Cyanobacteriota	Cyanophyceae	Chroococcales	Aphanothecaceae	Rippkaea	Rippkaea orientalis
Bacteria	Cyanobacteriota	Cyanophyceae	Synechococcales	Prochlorococcaceae	Cyanobium	Cyanobium sp.
Bacteria	Cyanobacteriota	Cyanophyceae	Chroococcales	Aphanothecaceae	Gloeothece	Gloeothece verrucosa
Bacteria	Cyanobacteriota	Cyanophyceae	Chroococcales	Geminocystaceae	Cyanobacterium	Cyanobacterium sp.
Bacteria	Cyanobacteriota	Cyanophyceae	Gomontiellales	Cyanothecaceae	Cyanothece	Cyanothece sp.
Bacteria	Cyanobacteriota	Cyanophyceae	Chroococcales	Aphanothecaceae	Crocosphaera	Crocosphaera subtropica
Bacteria	Cyanobacteriota	Cyanophyceae	Chroococcales	Aphanothecaceae	Candidatus Atelocyanobacterium	unclassified Candidatus Atelocyanobacterium species
Bacteria	Cyanobacteriota	Cyanophyceae	Nostocales	Aphanizomenonaceae	Dolichospermum	Dolichospermum compactum
Bacteria	Cyanobacteriota	Cyanophyceae	Nostocales	Aphanizomenonaceae	Dolichospermum	Dolichospermum flos-aquae
Bacteria	Cyanobacteriota	Cyanophyceae	Nostocales	Aphanizomenonaceae	Dolichospermum	Dolichospermum heterosporum
Bacteria	Cyanobacteriota	Cyanophyceae	Nostocales	Aphanizomenonaceae	Dolichospermum	Dolichospermum sp.
Bacteria	Cyanobacteriota	Cyanophyceae	Synechococcales	Synechococcaceae	Dactylococcopsis	Dactylococcopsis salina
Bacteria	Cyanobacteriota	Cyanophyceae	Chroococcales	Halothecacae	Euhalothece	Euhalothece natronophila

Bacteria	Cyanobacteriota	Cyanophyceae	Nostocales	Hapalosiphonaceae	Fischerella	Fischerella sp.
Bacteria	Cyanobacteriota	Cyanophyceae	Chroococcales	Chroococcaceae	Gloeocapsopsis	Gloeocapsopsis dulcis
Bacteria	Cyanobacteriota	Cyanophyceae	Chroococcales	Geminocystaceae	Geminocystis	Geminocystis sp.
Bacteria	Cyanobacteriota	Cyanophyceae	Geitlerinematales	Geitlerinemataceae	Geitlerinema	Geitlerinema sp.
Bacteria	Cyanobacteriota	Cyanophyceae	Gloeobacterales	Gloeobacteraceae	Gloeobacter	Gloeobacter kilaueensis
Bacteria	Cyanobacteriota	Cyanophyceae	Chroococcales	Chroococcaceae	Gloeocapsa	unclassified Gloeocapsa species
Bacteria	Cyanobacteriota	Cyanophyceae	Gloeomargaritales	Gloeomargaritaceae	Gloeomargarita	Gloeomargarita lithophora
Bacteria	Cyanobacteriota	Cyanophyceae	Gloeobacterales	Gloeobacteraceae	Gloeobacter	Gloeobacter morelensis
Bacteria	Cyanobacteriota	Cyanophyceae	Gloeobacterales	Gloeobacteraceae	Gloeobacter	Gloeobacter violaceus
Bacteria	Cyanobacteriota	Cyanophyceae	Chroococcales	Halothecacae	Halothece	Halothece sp.
Bacteria	Cyanobacteriota	Cyanophyceae	Nostocales	Nodulariaceae	Halotia	Halotia branconii
Bacteria	Cyanobacteriota	Cyanophyceae	Nodosilineales	Nodosilineaceae	Halomicronema	Halomicronema hongdechloris
Bacteria	Cyanobacteriota	Cyanophyceae	Leptolyngbyales	Leptolyngbyaceae	Kovacikia	Kovacikia minuta
Bacteria	Cyanobacteriota	Cyanophyceae	Leptolyngbyales	Leptolyngbyaceae	Leptolyngbya	Leptolyngbya boryana
Bacteria	Cyanobacteriota	Cyanophyceae	Leptolyngbyales	Leptolyngbyaceae	Leptolyngbya	Leptolyngbya sp.
Bacteria	Cyanobacteriota	Cyanophyceae	Oscillatoriales	Sirenicapillariaceae	Limnospira	Limnospira fusiformis
Bacteria	Cyanobacteriota	Cyanophyceae	Oscillatoriales	Sirenicapillariaceae	Limnospira	Limnospira indica
Bacteria	Cyanobacteriota	Cyanophyceae	Leptolyngbyales	Leptolyngbyaceae	Leptodesmis	Leptodesmis sichuanensis
Bacteria	Cyanobacteriota	Cyanophyceae	Leptolyngbyales	Leptolyngbyaceae	Leptothermofonsia	Leptothermofonsia sichuanensis
Bacteria	Cyanobacteriota	Cyanophyceae	Chroococcales	Microcystaceae	Microcystis	Microcystis aeruginosa
Bacteria	Cyanobacteriota	Cyanophyceae	Nostocales	Rivulariaceae	Microchaete	Microchaete diplosiphon
Bacteria	Cyanobacteriota	Cyanophyceae	Coleofasciculales	Coleofasciculaceae	Allocoleopsis	Allocoleopsis franciscana
Bacteria	Cyanobacteriota	Cyanophyceae	Chroococcales	Microcystaceae	Microcystis	Microcystis sp.
Bacteria	Cyanobacteriota	Cyanophyceae	Chroococcales	Microcystaceae	Microcystis	Microcystis panniformis
Bacteria	Cyanobacteriota	Cyanophyceae	Oscillatoriales	Oscillatoriaceae	Moorena	Moorena producens
Bacteria	Cyanobacteriota	Cyanophyceae	Oscillatoriales	Microcoleaceae	Microcoleus	Microcoleus vaginatus
Bacteria	Cyanobacteriota	Cyanophyceae	Chroococcales	Microcystaceae	Microcystis	Microcystis viridis
Bacteria	Cyanobacteriota	Cyanophyceae	Nostocales	unclassified Nostocales family	unclassified Nostocales genus	Nostocales cyanobacterium
Bacteria	Cyanobacteriota	Cyanophyceae	Nostocales	Nostocaceae	Nostoc	Nostoc carneum
Bacteria	Cyanobacteriota	Cyanophyceae	Nostocales	Nostocaceae	Nostoc	Nostoc edaphicum
Bacteria	Cyanobacteriota	Cyanophyceae	Nostocales	Nostocaceae	Nostoc	Nostoc flagelliforme
Bacteria	Cyanobacteriota	Cyanophyceae	Nostocales	Nostocaceae	Nostoc	Nostoc linckia
Bacteria	Cyanobacteriota	Cyanophyceae	Nostocales	Nostocaceae	Nostoc	Nostoc punctiforme
Bacteria	Cyanobacteriota	Cyanophyceae	Nostocales	Nostocaceae	Nostoc	Nostoc piscinale
Bacteria	Cyanobacteriota	Cyanophyceae	Nostocales	Nostocaceae	Nostoc	Nostoc sphaeroides
Bacteria	Cyanobacteriota	Cyanophyceae	Nostocales	Nodulariaceae	Nodularia	Nodularia spumigena
Bacteria	Cyanobacteriota	Cyanophyceae	Nostocales	Nodulariaceae	Nodularia	Nodularia sphaerocarpa
Bacteria	Cyanobacteriota	Cyanophyceae	Oscillatoriales	Oscillatoriaceae	Oscillatoria	Oscillatoria acuminata
Bacteria	Cyanobacteriota	Cyanophyceae	Nostocales	Aphanizomenonaceae	Okeanomitos	Okeanomitos corallinicola
Bacteria	Cyanobacteriota	Cyanophyceae	Oscillatoriales	Oscillatoriaceae	Oscillatoria	Oscillatoria nigro-viridis
Bacteria	Cyanobacteriota	Cyanophyceae	Oscillatoriales	Oscillatoriaceae	Oxynema	Oxynema aestuarii

Bacteria	Cyanobacteriota	Cyanophyceae	Oscillatoriales	Microcoleaceae	Planktothrix	Planktothrix agardhii
Bacteria	Cyanobacteriota	Cyanophyceae	Pseudanabaenales	Pseudanabaenaceae	Pseudanabaena	Pseudanabaena galeata
Bacteria	Cyanobacteriota	Cyanophyceae	Oscillatoriales	Oscillatoriaceae	Phormidium	Phormidium sp.
Bacteria	Cyanobacteriota	Cyanophyceae	Chroococcales	Geminocystaceae	Picosynechococcus	unclassified Picosynechococcus species
Bacteria	Cyanobacteriota	Cyanophyceae	Pleurocapsales	Hyellaceae	Pleurocapsa	Pleurocapsa sp.
Bacteria	Cyanobacteriota	Cyanophyceae	Synechococcales	Prochlorococcaceae	Prochlorococcus	Prochlorococcus marinus
Bacteria	Cyanobacteriota	Cyanophyceae	Oscillatoriales	Microcoleaceae	Planktothrix	Planktothrix pseudagardhii
Bacteria	Cyanobacteriota	Cyanophyceae	Synechococcales	Prochlorococcaceae	Prochlorococcus	Prochlorococcus sp.
Bacteria	Cyanobacteriota	Cyanophyceae	Oscillatoriales	Microcoleaceae	Planktothrix	Planktothrix rubescens
Bacteria	Cyanobacteriota	Cyanophyceae	Pseudanabaenales	Pseudanabaenaceae	Pseudanabaena	Pseudanabaena sp.
Bacteria	Cyanobacteriota	Cyanophyceae	Oscillatoriales	Oscillatoriaceae	Phormidium	Phormidium yuhuli
Bacteria	Cyanobacteriota	Cyanophyceae	Nostocales	Aphanizomenonaceae	Raphidiopsis	Raphidiopsis curvata
Bacteria	Cyanobacteriota	Cyanophyceae	Nostocales	Nostocaceae	Richelia	Richelia sinica
Bacteria	Cyanobacteriota	Cyanophyceae	Pleurocapsales	Dermocarpellaceae	Stanieria	Stanieria cyanosphaera
Bacteria	Cyanobacteriota	Cyanophyceae	Nostocales	Scytonemataceae	Scytonema	Scytonema sp.
Bacteria	Cyanobacteriota	Cyanophyceae	Nostocales	Aphanizomenonaceae	Sphaerospermopsis	Sphaerospermopsis kisseleviana
Bacteria	Cyanobacteriota	Cyanophyceae	Acaryochloridales	Thermosynechococcaceae	Parathermosynechococcus	Parathermosynechococcus lividus
Bacteria	Cyanobacteriota	Cyanophyceae	Pleurocapsales	Dermocarpellaceae	Stanieria	Stanieria sp.
Bacteria	Cyanobacteriota	Cyanophyceae	Nostocales	Aphanizomenonaceae	Sphaerospermopsis	Sphaerospermopsis torques-reginae
Bacteria	Cyanobacteriota	Cyanophyceae	Synechococcales	Synechococcaceae	Synechococcus	Synechococcus elongatus
Bacteria	Cyanobacteriota	Cyanophyceae	Synechococcales	Merismopediaceae	Synechocystis	Synechocystis sp.
Bacteria	Cyanobacteriota	Cyanophyceae	Synechococcales	Prochlorococcaceae	Parasynechococcus	Parasynechococcus marenigrum
Bacteria	Cyanobacteriota	Cyanophyceae	Acaryochloridales	Thermosynechococcaceae	Thermosynechococcus	Thermosynechococcus vestitus
Bacteria	Cyanobacteriota	Cyanophyceae	Oscillatoriales	Microcoleaceae	Trichodesmium	Trichodesmium erythraeum
Bacteria	Cyanobacteriota	Cyanophyceae	Acaryochloridales	Thermosynechococcaceae	Thermosynechococcus	Thermosynechococcus sp.
Bacteria	Cyanobacteriota	Cyanophyceae	Oculatellales	Oculatellaceae	Thermoleptolyngbya	Thermoleptolyngbya sichuanensis
Bacteria	Cyanobacteriota	Cyanophyceae	Oculatellales	Oculatellaceae	Thermoleptolyngbya	Thermoleptolyngbya oregonensis
Bacteria	Cyanobacteriota	Cyanophyceae	Nostocales	Tolypothrichaceae	Tolypothrix	Tolypothrix sp.
Bacteria	Cyanobacteriota	Cyanophyceae	Oculatellales	Oculatellaceae	Thermocoleostomius	Thermocoleostomius sinensis
Bacteria	Cyanobacteriota	Cyanophyceae	Acaryochloridales	Thermosynechococcaceae	Thermosynechococcus	Thermosynechococcus sichuanensis
Bacteria	Cyanobacteriota	Cyanophyceae	Leptolyngbyales	Trichocoleusaceae	Trichothermofontia	Trichothermofontia sichuanensis
Bacteria	Cyanobacteriota	Cyanophyceae	Nostocales	Tolypothrichaceae	Tolypothrix	Tolypothrix tenuis
Bacteria	Cyanobacteriota	Cyanophyceae	Thermostichales	Thermostichaceae	Thermostichus	Thermostichus vulcanus
Bacteria	Cyanobacteriota	Cyanophyceae	Synechococcales	Coelosphaeriaceae	Woronichinia	Woronichinia naegeliana
Eukaryota	unclassified Eukaryota	Pelagophyceae	Pelagomonadales	Pelagomonadaceae	Aureococcus	Aureococcus anophagefferens
Eukaryota	Streptophyta	Magnoliopsida	Fabales	Fabaceae	Arachis	Arachis duranensis
Eukaryota	Streptophyta	Magnoliopsida	Ericales	Actinidiaceae	Actinidia	Actinidia eriantha
Eukaryota	Streptophyta	Magnoliopsida	Fabales	Fabaceae	Arachis	Arachis hypogaea
Eukaryota	Streptophyta	Magnoliopsida	Brassicales	Brassicaceae	Arabidopsis	Arabidopsis lyrata
Eukaryota	Streptophyta	Magnoliopsida	Asparagales	Asparagaceae	Asparagus	Asparagus officinalis
Eukaryota	Streptophyta	Magnoliopsida	Lamiales	Acanthaceae	Andrographis	Andrographis paniculata

Eukaryota	Streptophyta	Magnoliopsida	Fabales	Fabaceae	Abrus	Abrus precatorius
Eukaryota	Chlorophyta	Trebouxiophyceae	Chlorellales	Chlorellaceae	Auxenochlorella	Auxenochlorella protothecoides
Eukaryota	Streptophyta	Magnoliopsida	Brassicales	Brassicaceae	Arabidopsis	Arabidopsis thaliana
Eukaryota	Streptophyta	Magnoliopsida	Amborellales	Amborellaceae	Amborella	Amborella trichopoda
Eukaryota	Streptophyta	Magnoliopsida	Caryophyllales	Amaranthaceae	Amaranthus	Amaranthus tricolor
Eukaryota	Streptophyta	Magnoliopsida	Poales	Poaceae	Brachypodium	Brachypodium distachyon
Eukaryota	Streptophyta	Magnoliopsida	Cucurbitales	Cucurbitaceae	Benincasa	Benincasa hispida
Eukaryota	Streptophyta	Magnoliopsida	Brassicales	Brassicaceae	Brassica	Brassica napus
Eukaryota	Streptophyta	Magnoliopsida	Brassicales	Brassicaceae	Brassica	Brassica rapa
Eukaryota	Streptophyta	Magnoliopsida	Fabales	Fabaceae	Cicer	Cicer arietinum
Eukaryota	Streptophyta	Magnoliopsida	Solanales	Solanaceae	Capsicum	Capsicum annuum
Eukaryota	Streptophyta	Magnoliopsida	Fagales	Betulaceae	Corylus	Corylus avellana
Eukaryota	Streptophyta	Magnoliopsida	Fabales	Fabaceae	Cajanus	Cajanus cajan
Eukaryota	Rhodophyta	Florideophyceae	Gigartinales	Gigartinaceae	Chondrus	Chondrus crispus
Eukaryota	Streptophyta	Magnoliopsida	Sapindales	Rutaceae	Citrus	Citrus sinensis
Eukaryota	Streptophyta	Magnoliopsida	Cucurbitales	Cucurbitaceae	Cucurbita	Cucurbita maxima
Eukaryota	Rhodophyta	Bangiophyceae	Cyanidiales	Cyanidiaceae	Cyanidioschyzon	Cyanidioschyzon merolae
Eukaryota	Streptophyta	Magnoliopsida	Cucurbitales	Cucurbitaceae	Cucurbita	Cucurbita moschata
Eukaryota	Streptophyta	Magnoliopsida	Brassicales	Caricaceae	Carica	Carica papaya
Eukaryota	Streptophyta	Magnoliopsida	Caryophyllales	Chenopodiaceae	Chenopodium	Chenopodium quinoa
Eukaryota	Streptophyta	Magnoliopsida	Brassicales	Brassicaceae	Capsella	Capsella rubella
Eukaryota	Chlorophyta	Chlorophyceae	Chlamydomonadales	Chlamydomonadaceae	Chlamydomonas	Chlamydomonas reinhardtii
Eukaryota	Streptophyta	Magnoliopsida	Brassicales	Brassicaceae	Camelina	Camelina sativa
Eukaryota	Streptophyta	Magnoliopsida	Ericales	Theaceae	Camellia	Camellia sinensis
Eukaryota	Chlorophyta	Trebouxiophyceae	unclassified Trebouxiophyceae	unclassified Trebouxiophyceae family	Coccomyxa	Coccomyxa subellipsoidea
Eukaryota	Streptophyta	Magnoliopsida	Cucurbitales	Cucurbitaceae	Cucumis	Cucumis sativus
Eukaryota	Chlorophyta	Trebouxiophyceae	Chlorellales	Chlorellaceae	Chlorella	Chlorella variabilis
Eukaryota	Streptophyta	Magnoliopsida	Apiales	Apiaceae	Daucus	Daucus carota
Eukaryota	Streptophyta	Magnoliopsida	Asparagales	Orchidaceae	Dendrobium	Dendrobium catenatum
Eukaryota	Chordata	Mammalia	Rodentia	Heteromyidae	Dipodomys	Dipodomys spectabilis
Eukaryota	Streptophyta	Magnoliopsida	Malvales	Malvaceae	Durio	Durio zibethinus
Eukaryota	Streptophyta	Magnoliopsida	Asterales	Asteraceae	Erigeron	Erigeron canadensis
Eukaryota	Streptophyta	Magnoliopsida	Myrtales	Myrtaceae	Eucalyptus	Eucalyptus grandis
Eukaryota	Streptophyta	Magnoliopsida	Arecales	Arecaceae	Elaeis	Elaeis guineensis
Eukaryota	Streptophyta	Magnoliopsida	Brassicales	Brassicaceae	Eutrema	Eutrema salsugineum
Eukaryota	Streptophyta	Magnoliopsida	Rosales	Rosaceae	Fragaria	Fragaria vesca
Eukaryota	Streptophyta	Magnoliopsida	Malvales	Malvaceae	Gossypium	Gossypium arboreum
Eukaryota	Streptophyta	Magnoliopsida	Malvales	Malvaceae	Gossypium	Gossypium hirsutum
Eukaryota	Streptophyta	Magnoliopsida	Fabales	Fabaceae	Glycine	Glycine max
Eukaryota	Streptophyta	Magnoliopsida	Malvales	Malvaceae	Gossypium	Gossypium raimondii
Eukaryota	Streptophyta	Magnoliopsida	Fabales	Fabaceae	Glycine	Glycine soja

Eukaryota	Rhodophyta	Bangiophyceae	Galdieriales	Galdieriaceae	Galdieria	Galdieria sulphuraria
Eukaryota	Streptophyta	Magnoliopsida	Asterales	Asteraceae	Helianthus	Helianthus annuus
Eukaryota	Streptophyta	Magnoliopsida	Malpighiales	Euphorbiaceae	Hevea	Hevea brasiliensis
Eukaryota	Streptophyta	Magnoliopsida	Malvales	Malvaceae	Hibiscus	Hibiscus syriacus
Eukaryota	Streptophyta	Magnoliopsida	Solanales	Convolvulaceae	Ipomoea	Ipomoea nil
Eukaryota	Streptophyta	Magnoliopsida	Solanales	Convolvulaceae	Ipomoea	Ipomoea triloba
Eukaryota	Streptophyta	Magnoliopsida	Malpighiales	Euphorbiaceae	Jatropha	Jatropha curcas
Eukaryota	Streptophyta	Magnoliopsida	Fagales	Juglandaceae	Juglans	Juglans regia
Eukaryota	Streptophyta	Magnoliopsida	Solanales	Solanaceae	Lycium	Lycium barbarum
Eukaryota	Streptophyta	Magnoliopsida	Fabales	Fabaceae	Lotus	Lotus japonicus
Eukaryota	Streptophyta	Magnoliopsida	Poales	Poaceae	Lolium	Lolium perenne
Eukaryota	Streptophyta	Magnoliopsida	Asterales	Asteraceae	Lactuca	Lactuca sativa
Eukaryota	Streptophyta	Magnoliopsida	Cucurbitales	Cucurbitaceae	Momordica	Momordica charantia
Eukaryota	Streptophyta	Magnoliopsida	Malpighiales	Euphorbiaceae	Mercurialis	Mercurialis annua
Eukaryota	Streptophyta	Magnoliopsida	Malpighiales	Euphorbiaceae	Manihot	Manihot esculenta
Eukaryota	Streptophyta	Magnoliopsida	Poales	Poaceae	Miscanthus	Miscanthus floridulus
Eukaryota	Streptophyta	Magnoliopsida	Sapindales	Anacardiaceae	Mangifera	Mangifera indica
Eukaryota	Streptophyta	Magnoliopsida	Proteales	Proteaceae	Macadamia	Macadamia integrifolia
Eukaryota	Streptophyta	Magnoliopsida	Rosales	Moraceae	Morus	Morus notabilis
Eukaryota	Streptophyta	Magnoliopsida	Santalales	Ximeniaceae	Malania	Malania oleifera
Eukaryota	Streptophyta	Magnoliopsida	Magnoliales	Magnoliaceae	Magnolia	Magnolia sinica
Eukaryota	Streptophyta	Magnoliopsida	Rosales	Rosaceae	Malus	Malus sylvestris
Eukaryota	Streptophyta	Magnoliopsida	Fabales	Fabaceae	Medicago	Medicago truncatula
Eukaryota	Streptophyta	Magnoliopsida	Solanales	Solanaceae	Nicotiana	Nicotiana attenuata
Eukaryota	Streptophyta	Magnoliopsida	Nymphaeales	Nymphaeaceae	Nymphaea	Nymphaea colorata
Eukaryota	Streptophyta	Magnoliopsida	Proteales	Nelumbonaceae	Nelumbo	Nelumbo nucifera
Eukaryota	Streptophyta	Magnoliopsida	Solanales	Solanaceae	Nicotiana	Nicotiana sylvestris
Eukaryota	Streptophyta	Magnoliopsida	Solanales	Solanaceae	Nicotiana	Nicotiana tabacum
Eukaryota	Streptophyta	Magnoliopsida	Solanales	Solanaceae	Nicotiana	Nicotiana tomentosiformis
Eukaryota	Streptophyta	Magnoliopsida	Poales	Poaceae	Oryza	Oryza brachyantha
Eukaryota	Streptophyta	Magnoliopsida	Poales	Poaceae	Oryza	Oryza glaberrima
Eukaryota	Streptophyta	Magnoliopsida	Poales	Poaceae	Oryza	Oryza sativa
Eukaryota	Chlorophyta	Mamiellophyceae	Mamiellales	Bathycoccaceae	Ostreococcus	Ostreococcus tauri
Eukaryota	Streptophyta	Magnoliopsida	Malpighiales	Salicaceae	Populus	Populus alba
Eukaryota	Streptophyta	Magnoliopsida	Fabales	Fabaceae	Prosopis	Prosopis cineraria
Eukaryota	Streptophyta	Magnoliopsida	Arecales	Arecaceae	Phoenix	Phoenix dactylifera
Eukaryota	Streptophyta	Magnoliopsida	Rosales	Rosaceae	Prunus	Prunus dulcis
Eukaryota	Streptophyta	Magnoliopsida	Asparagales	Orchidaceae	Phalaenopsis	Phalaenopsis equestris
Eukaryota	Streptophyta	Magnoliopsida	Malpighiales	Salicaceae	Populus	Populus euphratica
Eukaryota	Streptophyta	Magnoliopsida	Rosales	Rosaceae	Prunus	Prunus mume
Eukaryota	Streptophyta	Magnoliopsida	Malpighiales	Salicaceae	Populus	Populus trichocarpa
Eukaryota	Streptophyta	Magnoliopsida	Rosales	Rosaceae	Prunus	Prunus persica

Eukaryota	Streptophyta	Bryopsida	Funariales	Funariaceae	Physcomitrium	Physcomitrium patens
Eukaryota	Streptophyta	Magnoliopsida	Fabales	Fabaceae	Pisum	Pisum sativum
Eukaryota	Streptophyta	Magnoliopsida	Ranunculales	Papaveraceae	Papaver	Papaver somniferum
Eukaryota	Streptophyta	Magnoliopsida	Poales	Poaceae	Panicum	Panicum virgatum
Eukaryota	Streptophyta	Magnoliopsida	Fabales	Fabaceae	Phaseolus	Phaseolus vulgaris
Eukaryota	Streptophyta	Magnoliopsida	Sapindales	Anacardiaceae	Pistacia	Pistacia vera
Eukaryota	Streptophyta	Magnoliopsida	Fabales	Quillajaceae	Quillaja	Quillaja saponaria
Eukaryota	Streptophyta	Magnoliopsida	Malpighiales	Euphorbiaceae	Ricinus	Ricinus communis
Eukaryota	Streptophyta	Magnoliopsida	Brassicales	Brassicaceae	Raphanus	Raphanus sativus
Eukaryota	Streptophyta	Magnoliopsida	Poales	Poaceae	Sorghum	Sorghum bicolor
Eukaryota	Streptophyta	Magnoliopsida	Solanales	Solanaceae	Solanum	Solanum dulcamara
Eukaryota	Streptophyta	Magnoliopsida	Lamiales	Lamiaceae	Salvia	Salvia hispanica
Eukaryota	Streptophyta	Magnoliopsida	Lamiales	Pedaliaceae	Sesamum	Sesamum indicum
Eukaryota	Streptophyta	Magnoliopsida	Poales	Poaceae	Setaria	Setaria italica
Eukaryota	Streptophyta	Magnoliopsida	Solanales	Solanaceae	Solanum	Solanum lycopersicum
Eukaryota	Streptophyta	Magnoliopsida	Lamiales	Lamiaceae	Salvia	Salvia miltiorrhiza
Eukaryota	Streptophyta	Lycopodiopsida	Selaginellales	Selaginellaceae	Selaginella	Selaginella moellendorffii
Eukaryota	Streptophyta	Magnoliopsida	Caryophyllales	Chenopodiaceae	Spinacia	Spinacia oleracea
Eukaryota	Streptophyta	Magnoliopsida	Solanales	Solanaceae	Solanum	Solanum tuberosum
Eukaryota	Streptophyta	Magnoliopsida	Solanales	Solanaceae	Solanum	Solanum pennellii
Eukaryota	Streptophyta	Magnoliopsida	Lamiales	Lamiaceae	Salvia	Salvia splendens
Eukaryota	Streptophyta	Magnoliopsida	Solanales	Solanaceae	Solanum	Solanum stenotomum
Eukaryota	Streptophyta	Magnoliopsida	Poales	Poaceae	Setaria	Setaria viridis
Eukaryota	Streptophyta	Magnoliopsida	Poales	Poaceae	Triticum	Triticum aestivum
Eukaryota	Streptophyta	Magnoliopsida	Malvales	Malvaceae	Theobroma	Theobroma cacao
Eukaryota	Streptophyta	Magnoliopsida	Poales	Poaceae	Triticum	Triticum dicoccoides
Eukaryota	Streptophyta	Magnoliopsida	Brassicales	Cleomaceae	Tarenaya	Tarenaya hassleriana
Eukaryota	Streptophyta	Magnoliopsida	Fabales	Fabaceae	Trifolium	Trifolium pratense
Eukaryota	Bacillariophyta	Coscinodiscophyceae	Thalassiosirales	Thalassiosiraceae	Thalassiosira	Thalassiosira pseudonana
Eukaryota	Streptophyta	Magnoliopsida	Proteales	Proteaceae	Telopea	Telopea speciosissima
Eukaryota	Streptophyta	Magnoliopsida	Poales	Poaceae	Triticum	Triticum urartu
Eukaryota	Streptophyta	Magnoliopsida	Fabales	Fabaceae	Vigna	Vigna angularis
Eukaryota	Streptophyta	Magnoliopsida	Vitales	Vitaceae	Vitis	Vitis riparia
Eukaryota	Streptophyta	Magnoliopsida	Fabales	Fabaceae	Vigna	Vigna unguiculata
Eukaryota	Streptophyta	Magnoliopsida	Vitales	Vitaceae	Vitis	Vitis vinifera
Eukaryota	Streptophyta	Magnoliopsida	Rosales	Rhamnaceae	Ziziphus	Ziziphus jujuba
Eukaryota	Streptophyta	Magnoliopsida	Poales	Poaceae	Zea	Zea mays
Eukaryota	Streptophyta	Magnoliopsida	Zingiberales	Zingiberaceae	Zingiber	Zingiber officinale

Gene_Organisms: psaC

Kingdom	Phylum	Class	Order	Family	Gunus	Species
Archaea	Thermoproteota	Thermoprotei	Desulfurococcales	Desulfurococcaceae	Ignicoccus	Ignicoccus hospitalis
Archaea	Thermoproteota	Thermoprotei	Desulfurococcales	Desulfurococcaceae	Ignicoccus	Ignicoccus islandicus
Bacteria	Cyanobacteriota	Cyanophyceae	Acaryochloridales	Acaryochloridaceae	Acaryochloris	unclassified Acaryochloris species
Bacteria	Cyanobacteriota	Cyanophyceae	Nostocales	Nostocaceae	Anabaena	Anabaena cylindrica
Bacteria	Cyanobacteriota	Cyanophyceae	Nostocales	Nodulariaceae	Anabaenopsis	Anabaenopsis elenkinii
Bacteria	Cyanobacteriota	Cyanophyceae	Nostocales	Aphanizomenonaceae	Aphanizomenon	Aphanizomenon flos-aquae
Bacteria	Cyanobacteriota	Cyanophyceae	Nostocales	Fortieaceae	Aulosira	Aulosira laxa
Bacteria	Cyanobacteriota	Cyanophyceae	Acaryochloridales	Acaryochloridaceae	Acaryochloris	Acaryochloris marina
Bacteria	Cyanobacteriota	Cyanophyceae	Nostocales	Nostocaceae	Nostoc	Nostoc sp.
Bacteria	Cyanobacteriota	Cyanophyceae	Nostocales	Nostocaceae	Anabaena	Anabaena sp.
Bacteria	Cyanobacteriota	Cyanophyceae	Oscillatoriales	Microcoleaceae	Arthrospira	Arthrospira platensis
Bacteria	Cyanobacteriota	Cyanophyceae	Nostocales	Nostocaceae	Trichormus	Trichormus variabilis
Bacteria	Cyanobacteriota	Cyanophyceae	Nostocales	Scytonemataceae	Brasilonema	Brasilonema octagenarum
Bacteria	Cyanobacteriota	Cyanophyceae	Nostocales	Scytonemataceae	Brasilonema	Brasilonema sennae
Bacteria	Cyanobacteriota	Cyanophyceae	Nostocales	Calotrichaceae	Calothrix	Calothrix sp.
Bacteria	Cyanobacteriota	Cyanophyceae	Chroococcales	Geminocystaceae	Cyanobacterium	Cyanobacterium aponinum
Bacteria	Cyanobacteriota	Cyanophyceae	Nostocales	Aphanizomenonaceae	Cylindrospermopsis	Cylindrospermopsis curvispora
Bacteria	Cyanobacteriota	Cyanophyceae	Gomontiellales	Gomontiellaceae	Crinalium	Crinalium epipsammum
Bacteria	Cyanobacteriota	Cyanophyceae	Synechococcales	Prochlorococcaceae	Cyanobium	Cyanobium gracile
Bacteria	Cyanobacteriota	Cyanophyceae	Chroococcales	Chroococcaceae	Chondrocystis	unclassified Chondrocystis species
Bacteria	Cyanobacteriota	Cyanophyceae	Gomontiellales	Chamaesiphonaceae	Chamaesiphon	Chamaesiphon minutus
Bacteria	Cyanobacteriota	Cyanophyceae	Nostocales	Aphanizomenonaceae	Cylindrospermopsis	Cylindrospermopsis raciborskii
Bacteria	Cyanobacteriota	Cyanophyceae	Nostocales	Nostocaceae	Cylindrospermum	Cylindrospermum stagnale
Bacteria	Cyanobacteriota	Cyanophyceae	Chroococcales	Geminocystaceae	Cyanobacterium	Cyanobacterium stanieri
Bacteria	Cyanobacteriota	Cyanophyceae	Chroococcidiopsidales	Chroococcidiopsidaceae	Chroococcidiopsis	Chroococcidiopsis thermalis
Bacteria	Cyanobacteriota	Cyanophyceae	Chroococcales	Aphanothecaceae	Crocosphaera	Crocosphaera watsonii
Bacteria	Cyanobacteriota	Cyanophyceae	Synechococcales	Synechococcaceae	Synechococcus	Synechococcus sp.
Bacteria	Cyanobacteriota	Cyanophyceae	Chroococcales	Aphanothecaceae	Gloeothece	Gloeothece citriformis
Bacteria	Cyanobacteriota	Cyanophyceae	Chroococcales	Aphanothecaceae	Rippkaea	Rippkaea orientalis
Bacteria	Cyanobacteriota	Cyanophyceae	Synechococcales	Prochlorococcaceae	Cyanobium	Cyanobium sp.
Bacteria	Cyanobacteriota	Cyanophyceae	Chroococcales	Aphanothecaceae	Gloeothece	Gloeothece verrucosa
Bacteria	Cyanobacteriota	Cyanophyceae	Chroococcales	Geminocystaceae	Cyanobacterium	Cyanobacterium sp.
Bacteria	Cyanobacteriota	Cyanophyceae	Gomontiellales	Cyanothecaceae	Cyanothece	Cyanothece sp.
Bacteria	Cyanobacteriota	Cyanophyceae	Chroococcales	Aphanothecaceae	Crocosphaera	Crocosphaera subtropica
Bacteria	Cyanobacteriota	Cyanophyceae	Chroococcales	Aphanothecaceae	Candidatus Atelocyanobacterium	unclassified Candidatus Atelocyanobacterium species
Bacteria	Cyanobacteriota	Cyanophyceae	Nostocales	Aphanizomenonaceae	Dolichospermum	Dolichospermum compactum
Bacteria	Cyanobacteriota	Cyanophyceae	Nostocales	Aphanizomenonaceae	Dolichospermum	Dolichospermum flos-aquae
Bacteria	Cyanobacteriota	Cyanophyceae	Nostocales	Aphanizomenonaceae	Dolichospermum	Dolichospermum heterosporum
Bacteria	Cyanobacteriota	Cyanophyceae	Nostocales	Aphanizomenonaceae	Dolichospermum	Dolichospermum sp.

Bacteria	Cyanobacteriota	Cyanophyceae	Synechococcales	Synechococcaceae	Dactylococcopsis	Dactylococcopsis salina
Bacteria	Cyanobacteriota	Cyanophyceae	Chroococcales	Halothecacae	Euhalothece	Euhalothece natronophila
Bacteria	Cyanobacteriota	Cyanophyceae	Nostocales	Hapalosiphonaceae	Fischerella	Fischerella sp.
Bacteria	Cyanobacteriota	Cyanophyceae	Chroococcales	Chroococcaceae	Gloeocapsopsis	Gloeocapsopsis dulcis
Bacteria	Cyanobacteriota	Cyanophyceae	Chroococcales	Geminocystaceae	Geminocystis	Geminocystis sp.
Bacteria	Cyanobacteriota	Cyanophyceae	Geitlerinematales	Geitlerinemataceae	Geitlerinema	Geitlerinema sp.
Bacteria	Cyanobacteriota	Cyanophyceae	Gloeobacterales	Gloeobacteraceae	Gloeobacter	Gloeobacter kilaueensis
Bacteria	Cyanobacteriota	Cyanophyceae	Chroococcales	Chroococcaceae	Gloeocapsa	unclassified Gloeocapsa species
Bacteria	Cyanobacteriota	Cyanophyceae	Gloeomargaritales	Gloeomargaritaceae	Gloeomargarita	Gloeomargarita lithophora
Bacteria	Cyanobacteriota	Cyanophyceae	Gloeobacterales	Gloeobacteraceae	Gloeobacter	Gloeobacter morelensis
Bacteria	Planctomycetota	Planctomycetia	Gemmatales	Gemmataceae	Gemmata	Gemmata massiliana
Bacteria	Cyanobacteriota	Cyanophyceae	Gloeobacterales	Gloeobacteraceae	Gloeobacter	Gloeobacter violaceus
Bacteria	Cyanobacteriota	Cyanophyceae	Chroococcales	Halothecacae	Halothece	Halothece sp.
Bacteria	Cyanobacteriota	Cyanophyceae	Nostocales	Nodulariaceae	Halotia	Halotia branconii
Bacteria	Cyanobacteriota	Cyanophyceae	Nodosilineales	Nodosilineaceae	Halomicronema	Halomicronema hongdechloris
Bacteria	Cyanobacteriota	Cyanophyceae	Leptolyngbyales	Leptolyngbyaceae	Kovacikia	Kovacikia minuta
Bacteria	Cyanobacteriota	Cyanophyceae	Leptolyngbyales	Leptolyngbyaceae	Leptolyngbya	Leptolyngbya boryana
Bacteria	Cyanobacteriota	Cyanophyceae	Leptolyngbyales	Leptolyngbyaceae	Leptolyngbya	Leptolyngbya sp.
Bacteria	Cyanobacteriota	Cyanophyceae	Oscillatoriales	Sirenicapillariaceae	Limnospira	Limnospira fusiformis
Bacteria	Cyanobacteriota	Cyanophyceae	Oscillatoriales	Sirenicapillariaceae	Limnospira	Limnospira indica
Bacteria	Cyanobacteriota	Cyanophyceae	Leptolyngbyales	Leptolyngbyaceae	Leptodesmis	Leptodesmis sichuanensis
Bacteria	Cyanobacteriota	Cyanophyceae	Leptolyngbyales	Leptolyngbyaceae	Leptothermofonsia	Leptothermofonsia sichuanensis
Bacteria	Cyanobacteriota	Cyanophyceae	Chroococcales	Microcystaceae	Microcystis	Microcystis aeruginosa
Bacteria	Cyanobacteriota	Cyanophyceae	Nostocales	Rivulariaceae	Microchaete	Microchaete diplosiphon
Bacteria	Cyanobacteriota	Cyanophyceae	Coleofasciculales	Coleofasciculaceae	Allocoleopsis	Allocoleopsis franciscana
Bacteria	Cyanobacteriota	Cyanophyceae	Chroococcales	Microcystaceae	Microcystis	Microcystis sp.
Bacteria	Cyanobacteriota	Cyanophyceae	Chroococcales	Microcystaceae	Microcystis	Microcystis panniformis
Bacteria	Cyanobacteriota	Cyanophyceae	Oscillatoriales	Oscillatoriaceae	Moorena	Moorena producens
Bacteria	Cyanobacteriota	Cyanophyceae	Oscillatoriales	Microcoleaceae	Microcoleus	Microcoleus vaginatus
Bacteria	Cyanobacteriota	Cyanophyceae	Chroococcales	Microcystaceae	Microcystis	Microcystis viridis
Bacteria	Cyanobacteriota	Cyanophyceae	Nostocales	unclassified Nostocales family	unclassified Nostocales genus	Nostocales cyanobacterium
Bacteria	Cyanobacteriota	Cyanophyceae	Nostocales	Nostocaceae	Nostoc	Nostoc carneum
Bacteria	Cyanobacteriota	Cyanophyceae	Nostocales	Nostocaceae	Nostoc	Nostoc edaphicum
Bacteria	Cyanobacteriota	Cyanophyceae	Nostocales	Nostocaceae	Nostoc	Nostoc flagelliforme
Bacteria	Cyanobacteriota	Cyanophyceae	Nostocales	Nostocaceae	Nostoc	Nostoc linckia
Bacteria	Cyanobacteriota	Cyanophyceae	Nostocales	Nostocaceae	Nostoc	Nostoc punctiforme
Bacteria	Cyanobacteriota	Cyanophyceae	Nostocales	Nostocaceae	Nostoc	Nostoc piscinale
Bacteria	Cyanobacteriota	Cyanophyceae	Nostocales	Nostocaceae	Nostoc	Nostoc sphaeroides
Bacteria	Cyanobacteriota	Cyanophyceae	Nostocales	Nodulariaceae	Nodularia	Nodularia spumigena
Bacteria	Cyanobacteriota	Cyanophyceae	Nostocales	Nodulariaceae	Nodularia	Nodularia sphaerocarpa
Bacteria	Cyanobacteriota	Cyanophyceae	Oscillatoriales	Oscillatoriaceae	Oscillatoria	Oscillatoria acuminata

Bacteria	Cyanobacteriota	Cyanophyceae	Nostocales	Aphanizomenonaceae	Okeanomitos	Okeanomitos corallinicola
Bacteria	Cyanobacteriota	Cyanophyceae	Oscillatoriales	Oscillatoriaceae	Oscillatoria	Oscillatoria nigro-viridis
Bacteria	Cyanobacteriota	Cyanophyceae	Oscillatoriales	Oscillatoriaceae	Oxynema	Oxynema aestuarii
Bacteria	Cyanobacteriota	Cyanophyceae	Oscillatoriales	Microcoleaceae	Planktothrix	Planktothrix agardhii
Bacteria	Cyanobacteriota	Cyanophyceae	Pseudanabaenales	Pseudanabaenaceae	Pseudanabaena	Pseudanabaena galeata
Bacteria	Cyanobacteriota	Cyanophyceae	Oscillatoriales	Oscillatoriaceae	Phormidium	Phormidium sp.
Bacteria	Cyanobacteriota	Cyanophyceae	Chroococcales	Geminocystaceae	Picosynechococcus	unclassified Picosynechococcus species
Bacteria	Cyanobacteriota	Cyanophyceae	Pleurocapsales	Hyellaceae	Pleurocapsa	Pleurocapsa sp.
Bacteria	Cyanobacteriota	Cyanophyceae	Synechococcales	Prochlorococcaceae	Prochlorococcus	Prochlorococcus marinus
Bacteria	Cyanobacteriota	Cyanophyceae	Oscillatoriales	Microcoleaceae	Planktothrix	Planktothrix pseudagardhii
Bacteria	Cyanobacteriota	Cyanophyceae	Synechococcales	Prochlorococcaceae	Prochlorococcus	Prochlorococcus sp.
Bacteria	Cyanobacteriota	Cyanophyceae	Oscillatoriales	Microcoleaceae	Planktothrix	Planktothrix rubescens
Bacteria	Cyanobacteriota	Cyanophyceae	Pseudanabaenales	Pseudanabaenaceae	Pseudanabaena	Pseudanabaena sp.
Bacteria	Cyanobacteriota	Cyanophyceae	Oscillatoriales	Oscillatoriaceae	Phormidium	Phormidium yuhuli
Bacteria	Chloroflexota	Chloroflexia	Chloroflexales	Roseiflexaceae	Roseiflexus	Roseiflexus castenholzii
Bacteria	Cyanobacteriota	Cyanophyceae	Nostocales	Aphanizomenonaceae	Raphidiopsis	Raphidiopsis curvata
Bacteria	Cyanobacteriota	Cyanophyceae	Nostocales	Nostocaceae	Richelia	Richelia sinica
Bacteria	Cyanobacteriota	Cyanophyceae	Pleurocapsales	Dermocarpellaceae	Stanieria	Stanieria cyanosphaera
Bacteria	Cyanobacteriota	Cyanophyceae	Nostocales	Scytonemataceae	Scytonema	Scytonema sp.
Bacteria	Cyanobacteriota	Cyanophyceae	Nostocales	Aphanizomenonaceae	Sphaerospermopsis	Sphaerospermopsis kisseleviana
Bacteria	Cyanobacteriota	Cyanophyceae	Acaryochloridales	Thermosynechococcaceae	Parathermosynechococcu	Parathermosynechococcus lividus
Bacteria	Cyanobacteriota	Cyanophyceae	Pleurocapsales	Dermocarpellaceae	Stanieria	Stanieria sp.
Bacteria	Cyanobacteriota	Cyanophyceae	Nostocales	Aphanizomenonaceae	Sphaerospermopsis	Sphaerospermopsis torques-reginae
Bacteria	Cyanobacteriota	Cyanophyceae	Synechococcales	Synechococcaceae	Synechococcus	Synechococcus elongatus
Bacteria	Cyanobacteriota	Cyanophyceae	Synechococcales	Merismopediaceae	Synechocystis	Synechocystis sp.
Bacteria	Cyanobacteriota	Cyanophyceae	Synechococcales	Prochlorococcaceae	Parasynechococcus	Parasynechococcus marenigrum
Bacteria	Cyanobacteriota	Cyanophyceae	Acaryochloridales	Thermosynechococcaceae	Thermosynechococcus	Thermosynechococcus vestitus
Bacteria	Cyanobacteriota	Cyanophyceae	Oscillatoriales	Microcoleaceae	Trichodesmium	Trichodesmium erythraeum
Bacteria	Cyanobacteriota	Cyanophyceae	Acaryochloridales	Thermosynechococcaceae	Thermosynechococcus	Thermosynechococcus sp.
Bacteria	Cyanobacteriota	Cyanophyceae	Oculatellales	Oculatellaceae	Thermoleptolyngbya	Thermoleptolyngbya sichuanensis
Bacteria	Cyanobacteriota	Cyanophyceae	Oculatellales	Oculatellaceae	Thermoleptolyngbya	Thermoleptolyngbya oregonensis
Bacteria	Cyanobacteriota	Cyanophyceae	Nostocales	Tolypothrichaceae	Tolypothrix	Tolypothrix sp.
Bacteria	Cyanobacteriota	Cyanophyceae	Oculatellales	Oculatellaceae	Thermocoleostomius	Thermocoleostomius sinensis
Bacteria	Cyanobacteriota	Cyanophyceae	Acaryochloridales	Thermosynechococcaceae	Thermosynechococcus	Thermosynechococcus sichuanensis
Bacteria	Cyanobacteriota	Cyanophyceae	Leptolyngbyales	Trichocoleusaceae	Trichothermofontia	Trichothermofontia sichuanensis
Bacteria	Cyanobacteriota	Cyanophyceae	Nostocales	Tolypothrichaceae	Tolypothrix	Tolypothrix tenuis
Bacteria	Cyanobacteriota	Cyanophyceae	Thermostichales	Thermostichaceae	Thermostichus	Thermostichus vulcanus
Bacteria	Cyanobacteriota	Cyanophyceae	Synechococcales	Coelosphaeriaceae	Woronichinia	Woronichinia naegeliana
Eukaryota	unclassified Eukaryota	Pelagophyceae	Pelagomonadales	Pelagomonadaceae	Aureococcus	Aureococcus anophagefferens
Eukaryota	Streptophyta	Magnoliopsida	Fabales	Fabaceae	Arachis	Arachis duranensis
Eukaryota	Streptophyta	Magnoliopsida	Ericales	Actinidiaceae	Actinidia	Actinidia eriantha

Eukaryota	Streptophyta	Magnoliopsida	Fabales	Fabaceae	Arachis	Arachis hypogaea
Eukaryota	Streptophyta	Magnoliopsida	Brassicales	Brassicaceae	Arabidopsis	Arabidopsis lyrata
Eukaryota	Streptophyta	Magnoliopsida	Asparagales	Asparagaceae	Asparagus	Asparagus officinalis
Eukaryota	Streptophyta	Magnoliopsida	Lamiales	Acanthaceae	Andrographis	Andrographis paniculata
Eukaryota	Streptophyta	Magnoliopsida	Fabales	Fabaceae	Abrus	Abrus precatorius
Eukaryota	Chlorophyta	Trebouxiophyceae	Chlorellales	Chlorellaceae	Auxenochlorella	Auxenochlorella protothecoides
Eukaryota	Streptophyta	Magnoliopsida	Brassicales	Brassicaceae	Arabidopsis	Arabidopsis thaliana
Eukaryota	Streptophyta	Magnoliopsida	Amborellales	Amborellaceae	Amborella	Amborella trichopoda
Eukaryota	Streptophyta	Magnoliopsida	Caryophyllales	Amaranthaceae	Amaranthus	Amaranthus tricolor
Eukaryota	Streptophyta	Magnoliopsida	Poales	Poaceae	Brachypodium	Brachypodium distachyon
Eukaryota	Streptophyta	Magnoliopsida	Cucurbitales	Cucurbitaceae	Benincasa	Benincasa hispida
Eukaryota	Streptophyta	Magnoliopsida	Brassicales	Brassicaceae	Brassica	Brassica napus
Eukaryota	Chlorophyta	Mamiellophyceae	Mamiellales	Bathycoccaceae	Bathycoccus	Bathycoccus prasinos
Eukaryota	Streptophyta	Magnoliopsida	Brassicales	Brassicaceae	Brassica	Brassica rapa
Eukaryota	Streptophyta	Magnoliopsida	Fabales	Fabaceae	Cicer	Cicer arietinum
Eukaryota	Streptophyta	Magnoliopsida	Solanales	Solanaceae	Capsicum	Capsicum annuum
Eukaryota	Streptophyta	Magnoliopsida	Fagales	Betulaceae	Corylus	Corylus avellana
Eukaryota	Streptophyta	Magnoliopsida	Fabales	Fabaceae	Cajanus	Cajanus cajan
Eukaryota	Rhodophyta	Florideophyceae	Gigartinales	Gigartinaceae	Chondrus	Chondrus crispus
Eukaryota	Streptophyta	Magnoliopsida	Sapindales	Rutaceae	Citrus	Citrus sinensis
Eukaryota	Streptophyta	Magnoliopsida	Cucurbitales	Cucurbitaceae	Cucurbita	Cucurbita maxima
Eukaryota	Rhodophyta	Bangiophyceae	Cyanidiales	Cyanidiaceae	Cyanidioschyzon	Cyanidioschyzon merolae
Eukaryota	Streptophyta	Magnoliopsida	Cucurbitales	Cucurbitaceae	Cucurbita	Cucurbita moschata
Eukaryota	Streptophyta	Magnoliopsida	Brassicales	Caricaceae	Carica	Carica papaya
Eukaryota	Streptophyta	Magnoliopsida	Caryophyllales	Chenopodiaceae	Chenopodium	Chenopodium quinoa
Eukaryota	Streptophyta	Magnoliopsida	Brassicales	Brassicaceae	Capsella	Capsella rubella
Eukaryota	Chlorophyta	Chlorophyceae	Chlamydomonadales	Chlamydomonadaceae	Chlamydomonas	Chlamydomonas reinhardtii
Eukaryota	Streptophyta	Magnoliopsida	Brassicales	Brassicaceae	Camelina	Camelina sativa
Eukaryota	Streptophyta	Magnoliopsida	Ericales	Theaceae	Camellia	Camellia sinensis
Eukaryota	Chlorophyta	Trebouxiophyceae	unclassified Trebouxiophyceae	unclassified Trebouxiophyceae family	Coccomyxa	Coccomyxa subellipsoidea
Eukaryota	Streptophyta	Magnoliopsida	Cucurbitales	Cucurbitaceae	Cucumis	Cucumis sativus
Eukaryota	Chlorophyta	Trebouxiophyceae	Chlorellales	Chlorellaceae	Chlorella	Chlorella variabilis
Eukaryota	Streptophyta	Magnoliopsida	Apiales	Apiaceae	Daucus	Daucus carota
Eukaryota	Streptophyta	Magnoliopsida	Asparagales	Orchidaceae	Dendrobium	Dendrobium catenatum
Eukaryota	Streptophyta	Magnoliopsida	Malvales	Malvaceae	Durio	Durio zibethinus
Eukaryota	Streptophyta	Magnoliopsida	Asterales	Asteraceae	Erigeron	Erigeron canadensis
Eukaryota	Streptophyta	Magnoliopsida	Myrtales	Myrtaceae	Eucalyptus	Eucalyptus grandis
Eukaryota	Streptophyta	Magnoliopsida	Arecales	Arecaceae	Elaeis	Elaeis guineensis
Eukaryota	Streptophyta	Magnoliopsida	Brassicales	Brassicaceae	Eutrema	Eutrema salsugineum
Eukaryota	Streptophyta	Magnoliopsida	Rosales	Rosaceae	Fragaria	Fragaria vesca
Eukaryota	Streptophyta	Magnoliopsida	Malvales	Malvaceae	Gossypium	Gossypium arboreum

Eukaryota	Streptophyta	Magnoliopsida	Malvales	Malvaceae	Gossypium	Gossypium hirsutum
Eukaryota	Streptophyta	Magnoliopsida	Fabales	Fabaceae	Glycine	Glycine max
Eukaryota	Streptophyta	Magnoliopsida	Malvales	Malvaceae	Gossypium	Gossypium raimondii
Eukaryota	Streptophyta	Magnoliopsida	Fabales	Fabaceae	Glycine	Glycine soja
Eukaryota	Rhodophyta	Bangiophyceae	Galdieriales	Galdieriaceae	Galdieria	Galdieria sulphuraria
Eukaryota	Streptophyta	Magnoliopsida	Asterales	Asteraceae	Helianthus	Helianthus annuus
Eukaryota	Streptophyta	Magnoliopsida	Malpighiales	Euphorbiaceae	Hevea	Hevea brasiliensis
Eukaryota	Streptophyta	Magnoliopsida	Malvales	Malvaceae	Hibiscus	Hibiscus syriacus
Eukaryota	Streptophyta	Magnoliopsida	Solanales	Convolvulaceae	Ipomoea	Ipomoea nil
Eukaryota	Streptophyta	Magnoliopsida	Solanales	Convolvulaceae	Ipomoea	Ipomoea triloba
Eukaryota	Streptophyta	Magnoliopsida	Malpighiales	Euphorbiaceae	Jatropha	Jatropha curcas
Eukaryota	Streptophyta	Magnoliopsida	Fagales	Juglandaceae	Juglans	Juglans regia
Eukaryota	Streptophyta	Magnoliopsida	Solanales	Solanaceae	Lycium	Lycium barbarum
Eukaryota	Streptophyta	Magnoliopsida	Poales	Poaceae	Lolium	Lolium perenne
Eukaryota	Streptophyta	Magnoliopsida	Asterales	Asteraceae	Lactuca	Lactuca sativa
Eukaryota	Streptophyta	Magnoliopsida	Cucurbitales	Cucurbitaceae	Momordica	Momordica charantia
Eukaryota	Streptophyta	Magnoliopsida	Malpighiales	Euphorbiaceae	Manihot	Manihot esculenta
Eukaryota	Streptophyta	Magnoliopsida	Poales	Poaceae	Miscanthus	Miscanthus floridulus
Eukaryota	Streptophyta	Magnoliopsida	Sapindales	Anacardiaceae	Mangifera	Mangifera indica
Eukaryota	Streptophyta	Magnoliopsida	Proteales	Proteaceae	Macadamia	Macadamia integrifolia
Eukaryota	Streptophyta	Magnoliopsida	Rosales	Moraceae	Morus	Morus notabilis
Eukaryota	Streptophyta	Magnoliopsida	Magnoliales	Magnoliaceae	Magnolia	Magnolia sinica
Eukaryota	Streptophyta	Magnoliopsida	Rosales	Rosaceae	Malus	Malus sylvestris
Eukaryota	Streptophyta	Magnoliopsida	Fabales	Fabaceae	Medicago	Medicago truncatula
Eukaryota	Streptophyta	Magnoliopsida	Solanales	Solanaceae	Nicotiana	Nicotiana attenuata
Eukaryota	Streptophyta	Magnoliopsida	Nymphaeales	Nymphaeaceae	Nymphaea	Nymphaea colorata
Eukaryota	Streptophyta	Magnoliopsida	Proteales	Nelumbonaceae	Nelumbo	Nelumbo nucifera
Eukaryota	Streptophyta	Magnoliopsida	Solanales	Solanaceae	Nicotiana	Nicotiana sylvestris
Eukaryota	Streptophyta	Magnoliopsida	Solanales	Solanaceae	Nicotiana	Nicotiana tabacum
Eukaryota	Streptophyta	Magnoliopsida	Solanales	Solanaceae	Nicotiana	Nicotiana tomentosiformis
Eukaryota	Streptophyta	Magnoliopsida	Poales	Poaceae	Oryza	Oryza brachyantha
Eukaryota	Streptophyta	Magnoliopsida	Poales	Poaceae	Oryza	Oryza glaberrima
Eukaryota	Streptophyta	Magnoliopsida	Poales	Poaceae	Oryza	Oryza sativa
Eukaryota	Chlorophyta	Mamiellophyceae	Mamiellales	Bathycoccaceae	Ostreococcus	Ostreococcus tauri
Eukaryota	Streptophyta	Magnoliopsida	Malpighiales	Salicaceae	Populus	Populus alba
Eukaryota	Streptophyta	Magnoliopsida	Fabales	Fabaceae	Prosopis	Prosopis cineraria
Eukaryota	Streptophyta	Magnoliopsida	Arecales	Arecaceae	Phoenix	Phoenix dactylifera
Eukaryota	Streptophyta	Magnoliopsida	Rosales	Rosaceae	Prunus	Prunus dulcis
Eukaryota	Streptophyta	Magnoliopsida	Asparagales	Orchidaceae	Phalaenopsis	Phalaenopsis equestris
Eukaryota	Streptophyta	Magnoliopsida	Malpighiales	Salicaceae	Populus	Populus euphratica
Eukaryota	Streptophyta	Magnoliopsida	Rosales	Rosaceae	Prunus	Prunus mume
Eukaryota	Streptophyta	Magnoliopsida	Malpighiales	Salicaceae	Populus	Populus trichocarpa

Eukaryota	Streptophyta	Magnoliopsida	Rosales	Rosaceae	Prunus	Prunus persica
Eukaryota	Streptophyta	Bryopsida	Funariales	Funariaceae	Physcomitrium	Physcomitrium patens
Eukaryota	Streptophyta	Magnoliopsida	Ranunculales	Papaveraceae	Papaver	Papaver somniferum
Eukaryota	Streptophyta	Magnoliopsida	Poales	Poaceae	Panicum	Panicum virgatum
Eukaryota	Streptophyta	Magnoliopsida	Fabales	Fabaceae	Phaseolus	Phaseolus vulgaris
Eukaryota	Streptophyta	Magnoliopsida	Sapindales	Anacardiaceae	Pistacia	Pistacia vera
Eukaryota	Streptophyta	Magnoliopsida	Malpighiales	Euphorbiaceae	Ricinus	Ricinus communis
Eukaryota	Streptophyta	Magnoliopsida	Brassicales	Brassicaceae	Raphanus	Raphanus sativus
Eukaryota	Streptophyta	Magnoliopsida	Poales	Poaceae	Sorghum	Sorghum bicolor
Eukaryota	Streptophyta	Magnoliopsida	Solanales	Solanaceae	Solanum	Solanum dulcamara
Eukaryota	Streptophyta	Magnoliopsida	Lamiales	Lamiaceae	Salvia	Salvia hispanica
Eukaryota	Streptophyta	Magnoliopsida	Lamiales	Pedaliaceae	Sesamum	Sesamum indicum
Eukaryota	Streptophyta	Magnoliopsida	Poales	Poaceae	Setaria	Setaria italica
Eukaryota	Streptophyta	Magnoliopsida	Solanales	Solanaceae	Solanum	Solanum lycopersicum
Eukaryota	Streptophyta	Magnoliopsida	Lamiales	Lamiaceae	Salvia	Salvia miltiorrhiza
Eukaryota	Streptophyta	Lycopodiopsida	Selaginellales	Selaginellaceae	Selaginella	Selaginella moellendorffii
Eukaryota	Streptophyta	Magnoliopsida	Caryophyllales	Chenopodiaceae	Spinacia	Spinacia oleracea
Eukaryota	Streptophyta	Magnoliopsida	Solanales	Solanaceae	Solanum	Solanum tuberosum
Eukaryota	Streptophyta	Magnoliopsida	Solanales	Solanaceae	Solanum	Solanum pennellii
Eukaryota	Streptophyta	Magnoliopsida	Lamiales	Lamiaceae	Salvia	Salvia splendens
Eukaryota	Streptophyta	Magnoliopsida	Solanales	Solanaceae	Solanum	Solanum stenotomum
Eukaryota	Streptophyta	Magnoliopsida	Poales	Poaceae	Setaria	Setaria viridis
Eukaryota	Streptophyta	Magnoliopsida	Poales	Poaceae	Triticum	Triticum aestivum
Eukaryota	Streptophyta	Magnoliopsida	Malvales	Malvaceae	Theobroma	Theobroma cacao
Eukaryota	Streptophyta	Magnoliopsida	Brassicales	Cleomaceae	Tarenaya	Tarenaya hassleriana
Eukaryota	Streptophyta	Magnoliopsida	Fabales	Fabaceae	Trifolium	Trifolium pratense
Eukaryota	Bacillariophyta	Coscinodiscophyceae	Thalassiosirales	Thalassiosiraceae	Thalassiosira	Thalassiosira pseudonana
Eukaryota	Streptophyta	Magnoliopsida	Poales	Poaceae	Triticum	Triticum urartu
Eukaryota	Streptophyta	Magnoliopsida	Fabales	Fabaceae	Vigna	Vigna angularis
Eukaryota	Streptophyta	Magnoliopsida	Vitales	Vitaceae	Vitis	Vitis riparia
Eukaryota	Streptophyta	Magnoliopsida	Fabales	Fabaceae	Vigna	Vigna unguiculata
Eukaryota	Streptophyta	Magnoliopsida	Vitales	Vitaceae	Vitis	Vitis vinifera
Eukaryota	Streptophyta	Magnoliopsida	Rosales	Rhamnaceae	Ziziphus	Ziziphus jujuba
Eukaryota	Streptophyta	Magnoliopsida	Poales	Poaceae	Zea	Zea mays
Eukaryota	Streptophyta	Magnoliopsida	Zingiberales	Zingiberaceae	Zingiber	Zingiber officinale

Gene_Organisms: psbA

Kingdom	Phylum	Class	Order	Family	Gunus	Species
Bacteria	Cyanobacteriota	Cyanophyceae	Acaryochloridales	Acaryochloridaceae	Acaryochloris	unclassified Acaryochloris species
Bacteria	Cyanobacteriota	Cyanophyceae	Nostocales	Nostocaceae	Anabaena	Anabaena cylindrica
Bacteria	Cyanobacteriota	Cyanophyceae	Nostocales	Nodulariaceae	Anabaenopsis	Anabaenopsis elenkinii
Bacteria	Cyanobacteriota	Cyanophyceae	Nostocales	Aphanizomenonaceae	Aphanizomenon	Aphanizomenon flos-aquae
Bacteria	Cyanobacteriota	Cyanophyceae	Nostocales	Fortieaceae	Aulosira	Aulosira laxa
Bacteria	Cyanobacteriota	Cyanophyceae	Acaryochloridales	Acaryochloridaceae	Acaryochloris	Acaryochloris marina
Bacteria	Cyanobacteriota	Cyanophyceae	Nostocales	Nostocaceae	Nostoc	Nostoc sp.
Bacteria	Cyanobacteriota	Cyanophyceae	Nostocales	Nostocaceae	Anabaena	Anabaena sp.
Bacteria	Cyanobacteriota	Cyanophyceae	Oscillatoriales	Microcoleaceae	Arthrospira	Arthrospira platensis
Bacteria	Cyanobacteriota	Cyanophyceae	Nostocales	Nostocaceae	Trichormus	Trichormus variabilis
Bacteria	Cyanobacteriota	Cyanophyceae	Nostocales	Scytonemataceae	Brasilonema	Brasilonema octagenarum
Bacteria	Cyanobacteriota	Cyanophyceae	Nostocales	Scytonemataceae	Brasilonema	Brasilonema sennae
Bacteria	Cyanobacteriota	Cyanophyceae	Nostocales	Calotrichaceae	Calothrix	Calothrix sp.
Bacteria	Cyanobacteriota	Cyanophyceae	Chroococcales	Geminocystaceae	Cyanobacterium	Cyanobacterium aponinum
Bacteria	Cyanobacteriota	Cyanophyceae	Nostocales	Aphanizomenonaceae	Cylindrospermopsis	Cylindrospermopsis curvispora
Bacteria	Cyanobacteriota	Cyanophyceae	Gomontiellales	Gomontiellaceae	Crinalium	Crinalium epipsammum
Bacteria	Cyanobacteriota	Cyanophyceae	Synechococcales	Prochlorococcaceae	Cyanobium	Cyanobium gracile
Bacteria	Cyanobacteriota	Cyanophyceae	Chroococcales	Chroococcaceae	Chondrocystis	unclassified Chondrocystis species
Bacteria	Cyanobacteriota	Cyanophyceae	Gomontiellales	Chamaesiphonaceae	Chamaesiphon	Chamaesiphon minutus
Bacteria	Cyanobacteriota	Cyanophyceae	Nostocales	Aphanizomenonaceae	Cylindrospermopsis	Cylindrospermopsis raciborskii
Bacteria	Cyanobacteriota	Cyanophyceae	Nostocales	Nostocaceae	Cylindrospermum	Cylindrospermum stagnale
Bacteria	Cyanobacteriota	Cyanophyceae	Chroococcales	Geminocystaceae	Cyanobacterium	Cyanobacterium stanieri
Bacteria	Cyanobacteriota	Cyanophyceae	Chroococcidiopsidales	Chroococcidiopsidaceae	Chroococcidiopsis	Chroococcidiopsis thermalis
Bacteria	Cyanobacteriota	Cyanophyceae	Chroococcales	Aphanothecaceae	Crocosphaera	Crocosphaera watsonii
Bacteria	Cyanobacteriota	Cyanophyceae	Synechococcales	Synechococcaceae	Synechococcus	Synechococcus sp.
Bacteria	Cyanobacteriota	Cyanophyceae	Chroococcales	Aphanothecaceae	Gloeothece	Gloeothece citriformis
Bacteria	Cyanobacteriota	Cyanophyceae	Chroococcales	Aphanothecaceae	Rippkaea	Rippkaea orientalis
Bacteria	Cyanobacteriota	Cyanophyceae	Synechococcales	Prochlorococcaceae	Cyanobium	Cyanobium sp.
Bacteria	Cyanobacteriota	Cyanophyceae	Chroococcales	Aphanothecaceae	Gloeothece	Gloeothece verrucosa
Bacteria	Cyanobacteriota	Cyanophyceae	Chroococcales	Geminocystaceae	Cyanobacterium	Cyanobacterium sp.
Bacteria	Cyanobacteriota	Cyanophyceae	Gomontiellales	Cyanothecaceae	Cyanothece	Cyanothece sp.
Bacteria	Cyanobacteriota	Cyanophyceae	Chroococcales	Aphanothecaceae	Crocosphaera	Crocosphaera subtropica
Bacteria	Cyanobacteriota	Cyanophyceae	Nostocales	Aphanizomenonaceae	Dolichospermum	Dolichospermum compactum
Bacteria	Cyanobacteriota	Cyanophyceae	Nostocales	Aphanizomenonaceae	Dolichospermum	Dolichospermum flos-aquae
Bacteria	Cyanobacteriota	Cyanophyceae	Nostocales	Aphanizomenonaceae	Dolichospermum	Dolichospermum heterosporum
Bacteria	Cyanobacteriota	Cyanophyceae	Nostocales	Aphanizomenonaceae	Dolichospermum	Dolichospermum sp.
Bacteria	Cyanobacteriota	Cyanophyceae	Synechococcales	Synechococcaceae	Dactylococcopsis	Dactylococcopsis salina
Bacteria	Cyanobacteriota	Cyanophyceae	Chroococcales	Halothecacae	Euhalothece	Euhalothece natronophila
Bacteria	Cyanobacteriota	Cyanophyceae	Nostocales	Hapalosiphonaceae	Fischerella	Fischerella sp.
Bacteria	Cyanobacteriota	Cyanophyceae	Chroococcales	Chroococcaceae	Gloeocapsopsis	Gloeocapsopsis dulcis

Bacteria	Cyanobacteriota	Cyanophyceae	Chroococcales	Geminocystaceae	Geminocystis	Geminocystis sp.
Bacteria	Cyanobacteriota	Cyanophyceae	Geitlerinematales	Geitlerinemataceae	Geitlerinema	Geitlerinema sp.
Bacteria	Cyanobacteriota	Cyanophyceae	Gloeobacterales	Gloeobacteraceae	Gloeobacter	Gloeobacter kilaueensis
Bacteria	Cyanobacteriota	Cyanophyceae	Chroococcales	Chroococcaceae	Gloeocapsa	unclassified Gloeocapsa species
Bacteria	Cyanobacteriota	Cyanophyceae	Gloeomargaritales	Gloeomargaritaceae	Gloeomargarita	Gloeomargarita lithophora
Bacteria	Cyanobacteriota	Cyanophyceae	Gloeobacterales	Gloeobacteraceae	Gloeobacter	Gloeobacter morelensis
Bacteria	Cyanobacteriota	Cyanophyceae	Gloeobacterales	Gloeobacteraceae	Gloeobacter	Gloeobacter violaceus
Bacteria	Cyanobacteriota	Cyanophyceae	Chroococcales	Halothecacae	Halothece	Halothece sp.
Bacteria	Cyanobacteriota	Cyanophyceae	Nostocales	Nodulariaceae	Halotia	Halotia branconii
Bacteria	Cyanobacteriota	Cyanophyceae	Nodosilineales	Nodosilineaceae	Halomicronema	Halomicronema hongdechloris
Bacteria	Cyanobacteriota	Cyanophyceae	Leptolyngbyales	Leptolyngbyaceae	Kovacikia	Kovacikia minuta
Bacteria	Cyanobacteriota	Cyanophyceae	Leptolyngbyales	Leptolyngbyaceae	Leptolyngbya	Leptolyngbya boryana
Bacteria	Cyanobacteriota	Cyanophyceae	Leptolyngbyales	Leptolyngbyaceae	Leptolyngbya	Leptolyngbya sp.
Bacteria	Cyanobacteriota	Cyanophyceae	Oscillatoriales	Sirenicapillariaceae	Limnospira	Limnospira fusiformis
Bacteria	Cyanobacteriota	Cyanophyceae	Oscillatoriales	Sirenicapillariaceae	Limnospira	Limnospira indica
Bacteria	Cyanobacteriota	Cyanophyceae	Leptolyngbyales	Leptolyngbyaceae	Leptodesmis	Leptodesmis sichuanensis
Bacteria	Cyanobacteriota	Cyanophyceae	Leptolyngbyales	Leptolyngbyaceae	Leptothermofonsia	Leptothermofonsia sichuanensis
Bacteria	Cyanobacteriota	Cyanophyceae	Chroococcales	Microcystaceae	Microcystis	Microcystis aeruginosa
Bacteria	Cyanobacteriota	Cyanophyceae	Nostocales	Rivulariaceae	Microchaete	Microchaete diplosiphon
Bacteria	Cyanobacteriota	Cyanophyceae	Coleofasciculales	Coleofasciculaceae	Allocoleopsis	Allocoleopsis franciscana
Bacteria	Cyanobacteriota	Cyanophyceae	Chroococcales	Microcystaceae	Microcystis	Microcystis sp.
Bacteria	Cyanobacteriota	Cyanophyceae	Chroococcales	Microcystaceae	Microcystis	Microcystis panniformis
Bacteria	Cyanobacteriota	Cyanophyceae	Oscillatoriales	Oscillatoriaceae	Moorena	Moorena producens
Bacteria	Cyanobacteriota	Cyanophyceae	Oscillatoriales	Microcoleaceae	Microcoleus	Microcoleus vaginatus
Bacteria	Cyanobacteriota	Cyanophyceae	Chroococcales	Microcystaceae	Microcystis	Microcystis viridis
Bacteria	Cyanobacteriota	Cyanophyceae	Nostocales	unclassified Nostocales family	unclassified Nostocales genus	Nostocales cyanobacterium
Bacteria	Cyanobacteriota	Cyanophyceae	Nostocales	Nostocaceae	Nostoc	Nostoc carneum
Bacteria	Cyanobacteriota	Cyanophyceae	Nostocales	Nostocaceae	Nostoc	Nostoc edaphicum
Bacteria	Cyanobacteriota	Cyanophyceae	Nostocales	Nostocaceae	Nostoc	Nostoc flagelliforme
Bacteria	Cyanobacteriota	Cyanophyceae	Nostocales	Nostocaceae	Nostoc	Nostoc linckia
Bacteria	Cyanobacteriota	Cyanophyceae	Nostocales	Nostocaceae	Nostoc	Nostoc punctiforme
Bacteria	Cyanobacteriota	Cyanophyceae	Nostocales	Nostocaceae	Nostoc	Nostoc piscinale
Bacteria	Cyanobacteriota	Cyanophyceae	Nostocales	Nostocaceae	Nostoc	Nostoc sphaeroides
Bacteria	Cyanobacteriota	Cyanophyceae	Nostocales	Nodulariaceae	Nodularia	Nodularia spumigena
Bacteria	Cyanobacteriota	Cyanophyceae	Nostocales	Nodulariaceae	Nodularia	Nodularia sphaerocarpa
Bacteria	Cyanobacteriota	Cyanophyceae	Oscillatoriales	Oscillatoriaceae	Oscillatoria	Oscillatoria acuminata
Bacteria	Cyanobacteriota	Cyanophyceae	Nostocales	Aphanizomenonaceae	Okeanomitos	Okeanomitos corallinicola
Bacteria	Cyanobacteriota	Cyanophyceae	Oscillatoriales	Oscillatoriaceae	Oscillatoria	Oscillatoria nigro-viridis
Bacteria	Cyanobacteriota	Cyanophyceae	Oscillatoriales	Oscillatoriaceae	Oxynema	Oxynema aestuarii
Bacteria	Cyanobacteriota	Cyanophyceae	Oscillatoriales	Microcoleaceae	Planktothrix	Planktothrix agardhii
Bacteria	Cyanobacteriota	Cyanophyceae	Pseudanabaenales	Pseudanabaenaceae	Pseudanabaena	Pseudanabaena galeata

Bacteria	Cyanobacteriota	Cyanophyceae	Oscillatoriales	Oscillatoriaceae	Phormidium	Phormidium sp.
Bacteria	Cyanobacteriota	Cyanophyceae	Chroococcales	Geminocystaceae	Picosynechococcus	unclassified Picosynechococcus species
Bacteria	Cyanobacteriota	Cyanophyceae	Pleurocapsales	Hyellaceae	Pleurocapsa	Pleurocapsa sp.
Bacteria	Cyanobacteriota	Cyanophyceae	Synechococcales	Prochlorococcaceae	Prochlorococcus	Prochlorococcus marinus
Bacteria	Cyanobacteriota	Cyanophyceae	Oscillatoriales	Microcoleaceae	Planktothrix	Planktothrix pseudagardhii
Bacteria	Cyanobacteriota	Cyanophyceae	Synechococcales	Prochlorococcaceae	Prochlorococcus	Prochlorococcus sp.
Bacteria	Cyanobacteriota	Cyanophyceae	Oscillatoriales	Microcoleaceae	Planktothrix	Planktothrix rubescens
Bacteria	Cyanobacteriota	Cyanophyceae	Pseudanabaenales	Pseudanabaenaceae	Pseudanabaena	Pseudanabaena sp.
Bacteria	Cyanobacteriota	Cyanophyceae	Oscillatoriales	Oscillatoriaceae	Phormidium	Phormidium yuhuli
Bacteria	Cyanobacteriota	Cyanophyceae	Nostocales	Aphanizomenonaceae	Raphidiopsis	Raphidiopsis curvata
Bacteria	Cyanobacteriota	Cyanophyceae	Nostocales	Nostocaceae	Richelia	Richelia sinica
Bacteria	Cyanobacteriota	Cyanophyceae	Pleurocapsales	Dermocarpellaceae	Stanieria	Stanieria cyanosphaera
Bacteria	Cyanobacteriota	Cyanophyceae	Nostocales	Scytonemataceae	Scytonema	Scytonema sp.
Bacteria	Cyanobacteriota	Cyanophyceae	Nostocales	Aphanizomenonaceae	Sphaerospermopsis	Sphaerospermopsis kisseleviana
Bacteria	Cyanobacteriota	Cyanophyceae	Acaryochloridales	Thermosynechococcaceae	Parathermosynechococcu	Parathermosynechococcus lividus
Bacteria	Cyanobacteriota	Cyanophyceae	Pleurocapsales	Dermocarpellaceae	Stanieria	Stanieria sp.
Bacteria	Cyanobacteriota	Cyanophyceae	Nostocales	Aphanizomenonaceae	Sphaerospermopsis	Sphaerospermopsis torques-reginae
Bacteria	Cyanobacteriota	Cyanophyceae	Synechococcales	Synechococcaceae	Synechococcus	Synechococcus elongatus
Bacteria	Cyanobacteriota	Cyanophyceae	Synechococcales	Merismopediaceae	Synechocystis	Synechocystis sp.
Bacteria	Cyanobacteriota	Cyanophyceae	Synechococcales	Prochlorococcaceae	Parasynechococcus	Parasynechococcus marenigrum
Bacteria	Cyanobacteriota	Cyanophyceae	Acaryochloridales	Thermosynechococcaceae	Thermosynechococcus	Thermosynechococcus vestitus
Bacteria	Cyanobacteriota	Cyanophyceae	Oscillatoriales	Microcoleaceae	Trichodesmium	Trichodesmium erythraeum
Bacteria	Cyanobacteriota	Cyanophyceae	Acaryochloridales	Thermosynechococcaceae	Thermosynechococcus	Thermosynechococcus sp.
Bacteria	Cyanobacteriota	Cyanophyceae	Oculatellales	Oculatellaceae	Thermoleptolyngbya	Thermoleptolyngbya sichuanensis
Bacteria	Cyanobacteriota	Cyanophyceae	Oculatellales	Oculatellaceae	Thermoleptolyngbya	Thermoleptolyngbya oregonensis
Bacteria	Cyanobacteriota	Cyanophyceae	Nostocales	Tolypothrichaceae	Tolypothrix	Tolypothrix sp.
Bacteria	Cyanobacteriota	Cyanophyceae	Oculatellales	Oculatellaceae	Thermocoleostomius	Thermocoleostomius sinensis
Bacteria	Cyanobacteriota	Cyanophyceae	Acaryochloridales	Thermosynechococcaceae	Thermosynechococcus	Thermosynechococcus sichuanensis
Bacteria	Cyanobacteriota	Cyanophyceae	Leptolyngbyales	Trichocoleusaceae	Trichothermofontia	Trichothermofontia sichuanensis
Bacteria	Cyanobacteriota	Cyanophyceae	Nostocales	Tolypothrichaceae	Tolypothrix	Tolypothrix tenuis
Bacteria	Cyanobacteriota	Cyanophyceae	Thermostichales	Thermostichaceae	Thermostichus	Thermostichus vulcanus
Bacteria	Cyanobacteriota	Cyanophyceae	Synechococcales	Coelosphaeriaceae	Woronichinia	Woronichinia naegeliana
Eukaryota	unclassified Eukaryota	Pelagophyceae	Pelagomonadales	Pelagomonadaceae	Aureococcus	Aureococcus anophagefferens
Eukaryota	Streptophyta	Magnoliopsida	Fabales	Fabaceae	Arachis	Arachis duranensis
Eukaryota	Streptophyta	Magnoliopsida	Ericales	Actinidiaceae	Actinidia	Actinidia eriantha
Eukaryota	Streptophyta	Magnoliopsida	Fabales	Fabaceae	Arachis	Arachis hypogaea
Eukaryota	Streptophyta	Magnoliopsida	Brassicales	Brassicaceae	Arabidopsis	Arabidopsis lyrata
Eukaryota	Streptophyta	Magnoliopsida	Asparagales	Asparagaceae	Asparagus	Asparagus officinalis
Eukaryota	Streptophyta	Magnoliopsida	Lamiales	Acanthaceae	Andrographis	Andrographis paniculata
Eukaryota	Streptophyta	Magnoliopsida	Fabales	Fabaceae	Abrus	Abrus precatorius
Eukaryota	Chlorophyta	Trebouxiophyceae	Chlorellales	Chlorellaceae	Auxenochlorella	Auxenochlorella protothecoides

Eukaryota	Streptophyta	Magnoliopsida	Brassicales	Brassicaceae	Arabidopsis	Arabidopsis thaliana
Eukaryota	Streptophyta	Magnoliopsida	Amborellales	Amborellaceae	Amborella	Amborella trichopoda
Eukaryota	Streptophyta	Magnoliopsida	Caryophyllales	Amaranthaceae	Amaranthus	Amaranthus tricolor
Eukaryota	Streptophyta	Magnoliopsida	Poales	Poaceae	Brachypodium	Brachypodium distachyon
Eukaryota	Streptophyta	Magnoliopsida	Cucurbitales	Cucurbitaceae	Benincasa	Benincasa hispida
Eukaryota	Streptophyta	Magnoliopsida	Brassicales	Brassicaceae	Brassica	Brassica napus
Eukaryota	Chlorophyta	Mamiellophyceae	Mamiellales	Bathycoccaceae	Bathycoccus	Bathycoccus prasinos
Eukaryota	Streptophyta	Magnoliopsida	Brassicales	Brassicaceae	Brassica	Brassica rapa
Eukaryota	Streptophyta	Magnoliopsida	Fabales	Fabaceae	Cicer	Cicer arietinum
Eukaryota	Streptophyta	Magnoliopsida	Solanales	Solanaceae	Capsicum	Capsicum annuum
Eukaryota	Streptophyta	Magnoliopsida	Fagales	Betulaceae	Corylus	Corylus avellana
Eukaryota	Streptophyta	Magnoliopsida	Fabales	Fabaceae	Cajanus	Cajanus cajan
Eukaryota	Rhodophyta	Florideophyceae	Gigartinales	Gigartinaceae	Chondrus	Chondrus crispus
Eukaryota	Streptophyta	Magnoliopsida	Sapindales	Rutaceae	Citrus	Citrus sinensis
Eukaryota	Streptophyta	Magnoliopsida	Cucurbitales	Cucurbitaceae	Cucurbita	Cucurbita maxima
Eukaryota	Rhodophyta	Bangiophyceae	Cyanidiales	Cyanidiaceae	Cyanidioschyzon	Cyanidioschyzon merolae
Eukaryota	Streptophyta	Magnoliopsida	Cucurbitales	Cucurbitaceae	Cucurbita	Cucurbita moschata
Eukaryota	Streptophyta	Magnoliopsida	Brassicales	Caricaceae	Carica	Carica papaya
Eukaryota	Streptophyta	Magnoliopsida	Caryophyllales	Chenopodiaceae	Chenopodium	Chenopodium quinoa
Eukaryota	Streptophyta	Magnoliopsida	Brassicales	Brassicaceae	Capsella	Capsella rubella
Eukaryota	Chlorophyta	Chlorophyceae	Chlamydomonadales	Chlamydomonadaceae	Chlamydomonas	Chlamydomonas reinhardtii
Eukaryota	Streptophyta	Magnoliopsida	Brassicales	Brassicaceae	Camelina	Camelina sativa
Eukaryota	Streptophyta	Magnoliopsida	Ericales	Theaceae	Camellia	Camellia sinensis
Eukaryota	Chlorophyta	Trebouxiophyceae	unclassified Trebouxiophyceae	unclassified Trebouxiophyceae family	Coccomyxa	Coccomyxa subellipsoidea
Eukaryota	Streptophyta	Magnoliopsida	Cucurbitales	Cucurbitaceae	Cucumis	Cucumis sativus
Eukaryota	Chlorophyta	Trebouxiophyceae	Chlorellales	Chlorellaceae	Chlorella	Chlorella variabilis
Eukaryota	Streptophyta	Magnoliopsida	Apiales	Apiaceae	Daucus	Daucus carota
Eukaryota	Streptophyta	Magnoliopsida	Asparagales	Orchidaceae	Dendrobium	Dendrobium catenatum
Eukaryota	Chordata	Mammalia	Rodentia	Heteromyidae	Dipodomys	Dipodomys spectabilis
Eukaryota	Streptophyta	Magnoliopsida	Malvales	Malvaceae	Durio	Durio zibethinus
Eukaryota	Streptophyta	Magnoliopsida	Asterales	Asteraceae	Erigeron	Erigeron canadensis
Eukaryota	Streptophyta	Magnoliopsida	Myrtales	Myrtaceae	Eucalyptus	Eucalyptus grandis
Eukaryota	Streptophyta	Magnoliopsida	Arecales	Arecaceae	Elaeis	Elaeis guineensis
Eukaryota	Streptophyta	Magnoliopsida	Brassicales	Brassicaceae	Eutrema	Eutrema salsugineum
Eukaryota	Streptophyta	Magnoliopsida	Rosales	Rosaceae	Fragaria	Fragaria vesca
Eukaryota	Streptophyta	Magnoliopsida	Malvales	Malvaceae	Gossypium	Gossypium arboreum
Eukaryota	Streptophyta	Magnoliopsida	Malvales	Malvaceae	Gossypium	Gossypium hirsutum
Eukaryota	Streptophyta	Magnoliopsida	Fabales	Fabaceae	Glycine	Glycine max
Eukaryota	Streptophyta	Magnoliopsida	Malvales	Malvaceae	Gossypium	Gossypium raimondii
Eukaryota	Streptophyta	Magnoliopsida	Fabales	Fabaceae	Glycine	Glycine soja
Eukaryota	Rhodophyta	Bangiophyceae	Galdieriales	Galdieriaceae	Galdieria	Galdieria sulphuraria

Eukaryota	Streptophyta	Magnoliopsida	Asterales	Asteraceae	Helianthus	Helianthus annuus
Eukaryota	Streptophyta	Magnoliopsida	Malpighiales	Euphorbiaceae	Hevea	Hevea brasiliensis
Eukaryota	Streptophyta	Magnoliopsida	Malvales	Malvaceae	Hibiscus	Hibiscus syriacus
Eukaryota	Streptophyta	Magnoliopsida	Solanales	Convolvulaceae	Ipomoea	Ipomoea nil
Eukaryota	Streptophyta	Magnoliopsida	Solanales	Convolvulaceae	Ipomoea	Ipomoea triloba
Eukaryota	Streptophyta	Magnoliopsida	Malpighiales	Euphorbiaceae	Jatropha	Jatropha curcas
Eukaryota	Streptophyta	Magnoliopsida	Fagales	Juglandaceae	Juglans	Juglans regia
Eukaryota	Streptophyta	Magnoliopsida	Solanales	Solanaceae	Lycium	Lycium barbarum
Eukaryota	Streptophyta	Magnoliopsida	Fabales	Fabaceae	Lotus	Lotus japonicus
Eukaryota	Streptophyta	Magnoliopsida	Poales	Poaceae	Lolium	Lolium perenne
Eukaryota	Streptophyta	Magnoliopsida	Poales	Poaceae	Lolium	Lolium rigidum
Eukaryota	Streptophyta	Magnoliopsida	Asterales	Asteraceae	Lactuca	Lactuca sativa
Eukaryota	Streptophyta	Magnoliopsida	Cucurbitales	Cucurbitaceae	Momordica	Momordica charantia
Eukaryota	Streptophyta	Magnoliopsida	Malpighiales	Euphorbiaceae	Mercurialis	Mercurialis annua
Eukaryota	Streptophyta	Magnoliopsida	Malpighiales	Euphorbiaceae	Manihot	Manihot esculenta
Eukaryota	Streptophyta	Magnoliopsida	Poales	Poaceae	Miscanthus	Miscanthus floridulus
Eukaryota	Streptophyta	Magnoliopsida	Sapindales	Anacardiaceae	Mangifera	Mangifera indica
Eukaryota	Streptophyta	Magnoliopsida	Proteales	Proteaceae	Macadamia	Macadamia integrifolia
Eukaryota	Streptophyta	Magnoliopsida	Rosales	Moraceae	Morus	Morus notabilis
Eukaryota	Streptophyta	Magnoliopsida	Magnoliales	Magnoliaceae	Magnolia	Magnolia sinica
Eukaryota	Streptophyta	Magnoliopsida	Rosales	Rosaceae	Malus	Malus sylvestris
Eukaryota	Streptophyta	Magnoliopsida	Fabales	Fabaceae	Medicago	Medicago truncatula
Eukaryota	Streptophyta	Magnoliopsida	Solanales	Solanaceae	Nicotiana	Nicotiana attenuata
Eukaryota	Streptophyta	Magnoliopsida	Nymphaeales	Nymphaeaceae	Nymphaea	Nymphaea colorata
Eukaryota	Streptophyta	Magnoliopsida	Proteales	Nelumbonaceae	Nelumbo	Nelumbo nucifera
Eukaryota	Streptophyta	Magnoliopsida	Solanales	Solanaceae	Nicotiana	Nicotiana sylvestris
Eukaryota	Streptophyta	Magnoliopsida	Solanales	Solanaceae	Nicotiana	Nicotiana tabacum
Eukaryota	Streptophyta	Magnoliopsida	Solanales	Solanaceae	Nicotiana	Nicotiana tomentosiformis
Eukaryota	Streptophyta	Magnoliopsida	Poales	Poaceae	Oryza	Oryza brachyantha
Eukaryota	Streptophyta	Magnoliopsida	Lamiales	Oleaceae	Olea	Olea europaea
Eukaryota	Streptophyta	Magnoliopsida	Poales	Poaceae	Oryza	Oryza glaberrima
Eukaryota	Streptophyta	Magnoliopsida	Poales	Poaceae	Oryza	Oryza sativa
Eukaryota	Chlorophyta	Mamiellophyceae	Mamiellales	Bathycoccaceae	Ostreococcus	Ostreococcus tauri
Eukaryota	Streptophyta	Magnoliopsida	Malpighiales	Salicaceae	Populus	Populus alba
Eukaryota	Streptophyta	Magnoliopsida	Fabales	Fabaceae	Prosopis	Prosopis cineraria
Eukaryota	Streptophyta	Magnoliopsida	Arecales	Arecaceae	Phoenix	Phoenix dactylifera
Eukaryota	Streptophyta	Magnoliopsida	Rosales	Rosaceae	Prunus	Prunus dulcis
Eukaryota	Streptophyta	Magnoliopsida	Asparagales	Orchidaceae	Phalaenopsis	Phalaenopsis equestris
Eukaryota	Streptophyta	Magnoliopsida	Malpighiales	Salicaceae	Populus	Populus euphratica
Eukaryota	Streptophyta	Magnoliopsida	Rosales	Rosaceae	Prunus	Prunus mume
Eukaryota	Streptophyta	Magnoliopsida	Malpighiales	Salicaceae	Populus	Populus trichocarpa
Eukaryota	Streptophyta	Magnoliopsida	Rosales	Rosaceae	Prunus	Prunus persica

Eukaryota	Streptophyta	Bryopsida	Funariales	Funariaceae	Physcomitrium	Physcomitrium patens
Eukaryota	Streptophyta	Magnoliopsida	Fabales	Fabaceae	Pisum	Pisum sativum
Eukaryota	Streptophyta	Magnoliopsida	Ranunculales	Papaveraceae	Papaver	Papaver somniferum
Eukaryota	Streptophyta	Magnoliopsida	Poales	Poaceae	Panicum	Panicum virgatum
Eukaryota	Streptophyta	Magnoliopsida	Fabales	Fabaceae	Phaseolus	Phaseolus vulgaris
Eukaryota	Streptophyta	Magnoliopsida	Sapindales	Anacardiaceae	Pistacia	Pistacia vera
Eukaryota	Streptophyta	Magnoliopsida	Fabales	Quillajaceae	Quillaja	Quillaja saponaria
Eukaryota	Streptophyta	Magnoliopsida	Malpighiales	Euphorbiaceae	Ricinus	Ricinus communis
Eukaryota	Streptophyta	Magnoliopsida	Brassicales	Brassicaceae	Raphanus	Raphanus sativus
Eukaryota	Streptophyta	Magnoliopsida	Poales	Poaceae	Sorghum	Sorghum bicolor
Eukaryota	Streptophyta	Magnoliopsida	Solanales	Solanaceae	Solanum	Solanum dulcamara
Eukaryota	Streptophyta	Magnoliopsida	Lamiales	Lamiaceae	Salvia	Salvia hispanica
Eukaryota	Streptophyta	Magnoliopsida	Lamiales	Pedaliaceae	Sesamum	Sesamum indicum
Eukaryota	Streptophyta	Magnoliopsida	Poales	Poaceae	Setaria	Setaria italica
Eukaryota	Streptophyta	Magnoliopsida	Solanales	Solanaceae	Solanum	Solanum lycopersicum
Eukaryota	Streptophyta	Magnoliopsida	Lamiales	Lamiaceae	Salvia	Salvia miltiorrhiza
Eukaryota	Streptophyta	Lycopodiopsida	Selaginellales	Selaginellaceae	Selaginella	Selaginella moellendorffii
Eukaryota	Streptophyta	Magnoliopsida	Caryophyllales	Chenopodiaceae	Spinacia	Spinacia oleracea
Eukaryota	Streptophyta	Magnoliopsida	Solanales	Solanaceae	Solanum	Solanum tuberosum
Eukaryota	Streptophyta	Magnoliopsida	Solanales	Solanaceae	Solanum	Solanum pennellii
Eukaryota	Streptophyta	Magnoliopsida	Lamiales	Lamiaceae	Salvia	Salvia splendens
Eukaryota	Streptophyta	Magnoliopsida	Solanales	Solanaceae	Solanum	Solanum stenotomum
Eukaryota	Streptophyta	Magnoliopsida	Poales	Poaceae	Setaria	Setaria viridis
Eukaryota	Streptophyta	Magnoliopsida	Poales	Poaceae	Triticum	Triticum aestivum
Eukaryota	Streptophyta	Magnoliopsida	Malvales	Malvaceae	Theobroma	Theobroma cacao
Eukaryota	Streptophyta	Magnoliopsida	Poales	Poaceae	Triticum	Triticum dicoccoides
Eukaryota	Streptophyta	Magnoliopsida	Brassicales	Cleomaceae	Tarenaya	Tarenaya hassleriana
Eukaryota	Streptophyta	Magnoliopsida	Fabales	Fabaceae	Trifolium	Trifolium pratense
Eukaryota	Bacillariophyta	Coscinodiscophyceae	Thalassiosirales	Thalassiosiraceae	Thalassiosira	Thalassiosira pseudonana
Eukaryota	Streptophyta	Magnoliopsida	Proteales	Proteaceae	Telopea	Telopea speciosissima
Eukaryota	Streptophyta	Magnoliopsida	Poales	Poaceae	Triticum	Triticum urartu
Eukaryota	Streptophyta	Magnoliopsida	Celastrales	Celastraceae	Tripterygium	Tripterygium wilfordii
Eukaryota	Streptophyta	Magnoliopsida	Fabales	Fabaceae	Vigna	Vigna angularis
Eukaryota	Streptophyta	Magnoliopsida	Fabales	Fabaceae	Vigna	Vigna radiata
Eukaryota	Streptophyta	Magnoliopsida	Vitales	Vitaceae	Vitis	Vitis riparia
Eukaryota	Streptophyta	Magnoliopsida	Fabales	Fabaceae	Vigna	Vigna unguiculata
Eukaryota	Streptophyta	Magnoliopsida	Vitales	Vitaceae	Vitis	Vitis vinifera
Eukaryota	Streptophyta	Magnoliopsida	Rosales	Rhamnaceae	Ziziphus	Ziziphus jujuba
Eukaryota	Streptophyta	Magnoliopsida	Poales	Poaceae	Zea	Zea mays
Eukaryota	Streptophyta	Magnoliopsida	Zingiberales	Zingiberaceae	Zingiber	Zingiber officinale

Gene_Organisms: psbB

Kingdom	Phylum	Class	Order	Family	Gunus	Species
Bacteria	Cyanobacteriota	Cyanophyceae	Acaryochloridales	Acaryochloridaceae	Acaryochloris	unclassified Acaryochloris species
Bacteria	Cyanobacteriota	Cyanophyceae	Nostocales	Nostocaceae	Anabaena	Anabaena cylindrica
Bacteria	Cyanobacteriota	Cyanophyceae	Nostocales	Nodulariaceae	Anabaenopsis	Anabaenopsis elenkinii
Bacteria	Cyanobacteriota	Cyanophyceae	Nostocales	Aphanizomenonaceae	Aphanizomenon	Aphanizomenon flos-aquae
Bacteria	Cyanobacteriota	Cyanophyceae	Nostocales	Fortieaceae	Aulosira	Aulosira laxa
Bacteria	Cyanobacteriota	Cyanophyceae	Acaryochloridales	Acaryochloridaceae	Acaryochloris	Acaryochloris marina
Bacteria	Cyanobacteriota	Cyanophyceae	Nostocales	Nostocaceae	Nostoc	Nostoc sp.
Bacteria	Cyanobacteriota	Cyanophyceae	Nostocales	Nostocaceae	Anabaena	Anabaena sp.
Bacteria	Cyanobacteriota	Cyanophyceae	Oscillatoriales	Microcoleaceae	Arthrospira	Arthrospira platensis
Bacteria	Cyanobacteriota	Cyanophyceae	Nostocales	Nostocaceae	Trichormus	Trichormus variabilis
Bacteria	Cyanobacteriota	Cyanophyceae	Nostocales	Scytonemataceae	Brasilonema	Brasilonema octagenarum
Bacteria	Cyanobacteriota	Cyanophyceae	Nostocales	Scytonemataceae	Brasilonema	Brasilonema sennae
Bacteria	Cyanobacteriota	Cyanophyceae	Nostocales	Calotrichaceae	Calothrix	Calothrix sp.
Bacteria	Cyanobacteriota	Cyanophyceae	Chroococcales	Geminocystaceae	Cyanobacterium	Cyanobacterium aponinum
Bacteria	Cyanobacteriota	Cyanophyceae	Nostocales	Aphanizomenonaceae	Cylindrospermopsis	Cylindrospermopsis curvispora
Bacteria	Cyanobacteriota	Cyanophyceae	Gomontiellales	Gomontiellaceae	Crinalium	Crinalium epipsammum
Bacteria	Cyanobacteriota	Cyanophyceae	Synechococcales	Prochlorococcaceae	Cyanobium	Cyanobium gracile
Bacteria	Cyanobacteriota	Cyanophyceae	Chroococcales	Chroococcaceae	Chondrocystis	unclassified Chondrocystis species
Bacteria	Cyanobacteriota	Cyanophyceae	Gomontiellales	Chamaesiphonaceae	Chamaesiphon	Chamaesiphon minutus
Bacteria	Cyanobacteriota	Cyanophyceae	Nostocales	Aphanizomenonaceae	Cylindrospermopsis	Cylindrospermopsis raciborskii
Bacteria	Cyanobacteriota	Cyanophyceae	Nostocales	Nostocaceae	Cylindrospermum	Cylindrospermum stagnale
Bacteria	Cyanobacteriota	Cyanophyceae	Chroococcales	Geminocystaceae	Cyanobacterium	Cyanobacterium stanieri
Bacteria	Cyanobacteriota	Cyanophyceae	Chroococcidiopsidales	Chroococcidiopsidaceae	Chroococcidiopsis	Chroococcidiopsis thermalis
Bacteria	Cyanobacteriota	Cyanophyceae	Chroococcales	Aphanothecaceae	Crocosphaera	Crocosphaera watsonii
Bacteria	Cyanobacteriota	Cyanophyceae	Synechococcales	Synechococcaceae	Synechococcus	Synechococcus sp.
Bacteria	Cyanobacteriota	Cyanophyceae	Chroococcales	Aphanothecaceae	Gloeothece	Gloeothece citriformis
Bacteria	Cyanobacteriota	Cyanophyceae	Chroococcales	Aphanothecaceae	Rippkaea	Rippkaea orientalis
Bacteria	Cyanobacteriota	Cyanophyceae	Synechococcales	Prochlorococcaceae	Cyanobium	Cyanobium sp.
Bacteria	Cyanobacteriota	Cyanophyceae	Chroococcales	Aphanothecaceae	Gloeothece	Gloeothece verrucosa
Bacteria	Cyanobacteriota	Cyanophyceae	Chroococcales	Geminocystaceae	Cyanobacterium	Cyanobacterium sp.
Bacteria	Cyanobacteriota	Cyanophyceae	Gomontiellales	Cyanothecaceae	Cyanothece	Cyanothece sp.
Bacteria	Cyanobacteriota	Cyanophyceae	Chroococcales	Aphanothecaceae	Crocosphaera	Crocosphaera subtropica
Bacteria	Cyanobacteriota	Cyanophyceae	Nostocales	Aphanizomenonaceae	Dolichospermum	Dolichospermum compactum
Bacteria	Cyanobacteriota	Cyanophyceae	Nostocales	Aphanizomenonaceae	Dolichospermum	Dolichospermum flos-aquae
Bacteria	Cyanobacteriota	Cyanophyceae	Nostocales	Aphanizomenonaceae	Dolichospermum	Dolichospermum heterosporum
Bacteria	Cyanobacteriota	Cyanophyceae	Nostocales	Aphanizomenonaceae	Dolichospermum	Dolichospermum sp.
Bacteria	Cyanobacteriota	Cyanophyceae	Synechococcales	Synechococcaceae	Dactylococcopsis	Dactylococcopsis salina
Bacteria	Cyanobacteriota	Cyanophyceae	Chroococcales	Halothecacae	Euhalothece	Euhalothece natronophila
Bacteria	Cyanobacteriota	Cyanophyceae	Nostocales	Hapalosiphonaceae	Fischerella	Fischerella sp.
Bacteria	Cyanobacteriota	Cyanophyceae	Chroococcales	Chroococcaceae	Gloeocapsopsis	Gloeocapsopsis dulcis

Bacteria	Cyanobacteriota	Cyanophyceae	Chroococcales	Geminocystaceae	Geminocystis	Geminocystis sp.
Bacteria	Cyanobacteriota	Cyanophyceae	Geitlerinematales	Geitlerinemataceae	Geitlerinema	Geitlerinema sp.
Bacteria	Cyanobacteriota	Cyanophyceae	Gloeobacterales	Gloeobacteraceae	Gloeobacter	Gloeobacter kilaueensis
Bacteria	Cyanobacteriota	Cyanophyceae	Chroococcales	Chroococcaceae	Gloeocapsa	unclassified Gloeocapsa species
Bacteria	Cyanobacteriota	Cyanophyceae	Gloeomargaritales	Gloeomargaritaceae	Gloeomargarita	Gloeomargarita lithophora
Bacteria	Cyanobacteriota	Cyanophyceae	Gloeobacterales	Gloeobacteraceae	Gloeobacter	Gloeobacter morelensis
Bacteria	Cyanobacteriota	Cyanophyceae	Gloeobacterales	Gloeobacteraceae	Gloeobacter	Gloeobacter violaceus
Bacteria	Cyanobacteriota	Cyanophyceae	Chroococcales	Halothecacae	Halothece	Halothece sp.
Bacteria	Cyanobacteriota	Cyanophyceae	Nostocales	Nodulariaceae	Halotia	Halotia branconii
Bacteria	Cyanobacteriota	Cyanophyceae	Nodosilineales	Nodosilineaceae	Halomicronema	Halomicronema hongdechloris
Bacteria	Cyanobacteriota	Cyanophyceae	Leptolyngbyales	Leptolyngbyaceae	Kovacikia	Kovacikia minuta
Bacteria	Cyanobacteriota	Cyanophyceae	Leptolyngbyales	Leptolyngbyaceae	Leptolyngbya	Leptolyngbya boryana
Bacteria	Cyanobacteriota	Cyanophyceae	Leptolyngbyales	Leptolyngbyaceae	Leptolyngbya	Leptolyngbya sp.
Bacteria	Cyanobacteriota	Cyanophyceae	Oscillatoriales	Sirenicapillariaceae	Limnospira	Limnospira fusiformis
Bacteria	Cyanobacteriota	Cyanophyceae	Oscillatoriales	Sirenicapillariaceae	Limnospira	Limnospira indica
Bacteria	Cyanobacteriota	Cyanophyceae	Leptolyngbyales	Leptolyngbyaceae	Leptodesmis	Leptodesmis sichuanensis
Bacteria	Cyanobacteriota	Cyanophyceae	Leptolyngbyales	Leptolyngbyaceae	Leptothermofonsia	Leptothermofonsia sichuanensis
Bacteria	Cyanobacteriota	Cyanophyceae	Chroococcales	Microcystaceae	Microcystis	Microcystis aeruginosa
Bacteria	Cyanobacteriota	Cyanophyceae	Nostocales	Rivulariaceae	Microchaete	Microchaete diplosiphon
Bacteria	Cyanobacteriota	Cyanophyceae	Coleofasciculales	Coleofasciculaceae	Allocoleopsis	Allocoleopsis franciscana
Bacteria	Cyanobacteriota	Cyanophyceae	Chroococcales	Microcystaceae	Microcystis	Microcystis sp.
Bacteria	Cyanobacteriota	Cyanophyceae	Chroococcales	Microcystaceae	Microcystis	Microcystis panniformis
Bacteria	Cyanobacteriota	Cyanophyceae	Oscillatoriales	Oscillatoriaceae	Moorena	Moorena producens
Bacteria	Cyanobacteriota	Cyanophyceae	Oscillatoriales	Microcoleaceae	Microcoleus	Microcoleus vaginatus
Bacteria	Cyanobacteriota	Cyanophyceae	Chroococcales	Microcystaceae	Microcystis	Microcystis viridis
Bacteria	Cyanobacteriota	Cyanophyceae	Nostocales	unclassified Nostocales family	unclassified Nostocales genus	Nostocales cyanobacterium
Bacteria	Cyanobacteriota	Cyanophyceae	Nostocales	Nostocaceae	Nostoc	Nostoc carneum
Bacteria	Cyanobacteriota	Cyanophyceae	Nostocales	Nostocaceae	Nostoc	Nostoc edaphicum
Bacteria	Cyanobacteriota	Cyanophyceae	Nostocales	Nostocaceae	Nostoc	Nostoc flagelliforme
Bacteria	Cyanobacteriota	Cyanophyceae	Nostocales	Nostocaceae	Nostoc	Nostoc linckia
Bacteria	Cyanobacteriota	Cyanophyceae	Nostocales	Nostocaceae	Nostoc	Nostoc punctiforme
Bacteria	Cyanobacteriota	Cyanophyceae	Nostocales	Nostocaceae	Nostoc	Nostoc piscinale
Bacteria	Cyanobacteriota	Cyanophyceae	Nostocales	Nostocaceae	Nostoc	Nostoc sphaeroides
Bacteria	Cyanobacteriota	Cyanophyceae	Nostocales	Nodulariaceae	Nodularia	Nodularia spumigena
Bacteria	Cyanobacteriota	Cyanophyceae	Nostocales	Nodulariaceae	Nodularia	Nodularia sphaerocarpa
Bacteria	Cyanobacteriota	Cyanophyceae	Oscillatoriales	Oscillatoriaceae	Oscillatoria	Oscillatoria acuminata
Bacteria	Cyanobacteriota	Cyanophyceae	Nostocales	Aphanizomenonaceae	Okeanomitos	Okeanomitos corallinicola
Bacteria	Cyanobacteriota	Cyanophyceae	Oscillatoriales	Oscillatoriaceae	Oscillatoria	Oscillatoria nigro-viridis
Bacteria	Cyanobacteriota	Cyanophyceae	Oscillatoriales	Oscillatoriaceae	Oxynema	Oxynema aestuarii
Bacteria	Cyanobacteriota	Cyanophyceae	Oscillatoriales	Microcoleaceae	Planktothrix	Planktothrix agardhii
Bacteria	Cyanobacteriota	Cyanophyceae	Pseudanabaenales	Pseudanabaenaceae	Pseudanabaena	Pseudanabaena galeata

Bacteria	Cyanobacteriota	Cyanophyceae	Oscillatoriales	Oscillatoriaceae	Phormidium	Phormidium sp.
Bacteria	Cyanobacteriota	Cyanophyceae	Chroococcales	Geminocystaceae	Picosynechococcus	unclassified Picosynechococcus species
Bacteria	Cyanobacteriota	Cyanophyceae	Pleurocapsales	Hyellaceae	Pleurocapsa	Pleurocapsa sp.
Bacteria	Cyanobacteriota	Cyanophyceae	Synechococcales	Prochlorococcaceae	Prochlorococcus	Prochlorococcus marinus
Bacteria	Cyanobacteriota	Cyanophyceae	Oscillatoriales	Microcoleaceae	Planktothrix	Planktothrix pseudagardhii
Bacteria	Cyanobacteriota	Cyanophyceae	Synechococcales	Prochlorococcaceae	Prochlorococcus	Prochlorococcus sp.
Bacteria	Cyanobacteriota	Cyanophyceae	Oscillatoriales	Microcoleaceae	Planktothrix	Planktothrix rubescens
Bacteria	Cyanobacteriota	Cyanophyceae	Pseudanabaenales	Pseudanabaenaceae	Pseudanabaena	Pseudanabaena sp.
Bacteria	Cyanobacteriota	Cyanophyceae	Oscillatoriales	Oscillatoriaceae	Phormidium	Phormidium yuhuli
Bacteria	Cyanobacteriota	Cyanophyceae	Nostocales	Aphanizomenonaceae	Raphidiopsis	Raphidiopsis curvata
Bacteria	Cyanobacteriota	Cyanophyceae	Nostocales	Nostocaceae	Richelia	Richelia sinica
Bacteria	Cyanobacteriota	Cyanophyceae	Pleurocapsales	Dermocarpellaceae	Stanieria	Stanieria cyanosphaera
Bacteria	Cyanobacteriota	Cyanophyceae	Nostocales	Scytonemataceae	Scytonema	Scytonema sp.
Bacteria	Cyanobacteriota	Cyanophyceae	Nostocales	Aphanizomenonaceae	Sphaerospermopsis	Sphaerospermopsis kisseleviana
Bacteria	Cyanobacteriota	Cyanophyceae	Acaryochloridales	Thermosynechococcaceae	Parathermosynechococcu	Parathermosynechococcus lividus
Bacteria	Cyanobacteriota	Cyanophyceae	Pleurocapsales	Dermocarpellaceae	Stanieria	Stanieria sp.
Bacteria	Cyanobacteriota	Cyanophyceae	Nostocales	Aphanizomenonaceae	Sphaerospermopsis	Sphaerospermopsis torques-reginae
Bacteria	Cyanobacteriota	Cyanophyceae	Synechococcales	Synechococcaceae	Synechococcus	Synechococcus elongatus
Bacteria	Cyanobacteriota	Cyanophyceae	Synechococcales	Merismopediaceae	Synechocystis	Synechocystis sp.
Bacteria	Cyanobacteriota	Cyanophyceae	Synechococcales	Prochlorococcaceae	Parasynechococcus	Parasynechococcus marenigrum
Bacteria	Cyanobacteriota	Cyanophyceae	Acaryochloridales	Thermosynechococcaceae	Thermosynechococcus	Thermosynechococcus vestitus
Bacteria	Cyanobacteriota	Cyanophyceae	Oscillatoriales	Microcoleaceae	Trichodesmium	Trichodesmium erythraeum
Bacteria	Cyanobacteriota	Cyanophyceae	Acaryochloridales	Thermosynechococcaceae	Thermosynechococcus	Thermosynechococcus sp.
Bacteria	Cyanobacteriota	Cyanophyceae	Oculatellales	Oculatellaceae	Thermoleptolyngbya	Thermoleptolyngbya sichuanensis
Bacteria	Cyanobacteriota	Cyanophyceae	Oculatellales	Oculatellaceae	Thermoleptolyngbya	Thermoleptolyngbya oregonensis
Bacteria	Cyanobacteriota	Cyanophyceae	Nostocales	Tolypothrichaceae	Tolypothrix	Tolypothrix sp.
Bacteria	Cyanobacteriota	Cyanophyceae	Oculatellales	Oculatellaceae	Thermocoleostomius	Thermocoleostomius sinensis
Bacteria	Cyanobacteriota	Cyanophyceae	Acaryochloridales	Thermosynechococcaceae	Thermosynechococcus	Thermosynechococcus sichuanensis
Bacteria	Cyanobacteriota	Cyanophyceae	Leptolyngbyales	Trichocoleusaceae	Trichothermofontia	Trichothermofontia sichuanensis
Bacteria	Cyanobacteriota	Cyanophyceae	Nostocales	Tolypothrichaceae	Tolypothrix	Tolypothrix tenuis
Bacteria	Cyanobacteriota	Cyanophyceae	Thermostichales	Thermostichaceae	Thermostichus	Thermostichus vulcanus
Bacteria	Cyanobacteriota	Cyanophyceae	Synechococcales	Coelosphaeriaceae	Woronichinia	Woronichinia naegeliana
Eukaryota	unclassified Eukaryota	Pelagophyceae	Pelagomonadales	Pelagomonadaceae	Aureococcus	Aureococcus anophagefferens
Eukaryota	Streptophyta	Magnoliopsida	Fabales	Fabaceae	Arachis	Arachis duranensis
Eukaryota	Streptophyta	Magnoliopsida	Ericales	Actinidiaceae	Actinidia	Actinidia eriantha
Eukaryota	Streptophyta	Magnoliopsida	Fabales	Fabaceae	Arachis	Arachis hypogaea
Eukaryota	Streptophyta	Magnoliopsida	Brassicales	Brassicaceae	Arabidopsis	Arabidopsis lyrata
Eukaryota	Streptophyta	Magnoliopsida	Asparagales	Asparagaceae	Asparagus	Asparagus officinalis
Eukaryota	Streptophyta	Magnoliopsida	Lamiales	Acanthaceae	Andrographis	Andrographis paniculata
Eukaryota	Streptophyta	Magnoliopsida	Fabales	Fabaceae	Abrus	Abrus precatorius
Eukaryota	Chlorophyta	Trebouxiophyceae	Chlorellales	Chlorellaceae	Auxenochlorella	Auxenochlorella protothecoides

Eukaryota	Streptophyta	Magnoliopsida	Brassicales	Brassicaceae	Arabidopsis	Arabidopsis thaliana
Eukaryota	Streptophyta	Magnoliopsida	Amborellales	Amborellaceae	Amborella	Amborella trichopoda
Eukaryota	Streptophyta	Magnoliopsida	Caryophyllales	Amaranthaceae	Amaranthus	Amaranthus tricolor
Eukaryota	Streptophyta	Magnoliopsida	Poales	Poaceae	Brachypodium	Brachypodium distachyon
Eukaryota	Streptophyta	Magnoliopsida	Cucurbitales	Cucurbitaceae	Benincasa	Benincasa hispida
Eukaryota	Streptophyta	Magnoliopsida	Brassicales	Brassicaceae	Brassica	Brassica napus
Eukaryota	Chlorophyta	Mamiellophyceae	Mamiellales	Bathycoccaceae	Bathycoccus	Bathycoccus prasinos
Eukaryota	Streptophyta	Magnoliopsida	Brassicales	Brassicaceae	Brassica	Brassica rapa
Eukaryota	Streptophyta	Magnoliopsida	Fabales	Fabaceae	Cicer	Cicer arietinum
Eukaryota	Streptophyta	Magnoliopsida	Solanales	Solanaceae	Capsicum	Capsicum annuum
Eukaryota	Streptophyta	Magnoliopsida	Fagales	Betulaceae	Corylus	Corylus avellana
Eukaryota	Streptophyta	Magnoliopsida	Fabales	Fabaceae	Cajanus	Cajanus cajan
Eukaryota	Rhodophyta	Florideophyceae	Gigartinales	Gigartinaceae	Chondrus	Chondrus crispus
Eukaryota	Streptophyta	Magnoliopsida	Sapindales	Rutaceae	Citrus	Citrus sinensis
Eukaryota	Streptophyta	Magnoliopsida	Cucurbitales	Cucurbitaceae	Cucurbita	Cucurbita maxima
Eukaryota	Rhodophyta	Bangiophyceae	Cyanidiales	Cyanidiaceae	Cyanidioschyzon	Cyanidioschyzon merolae
Eukaryota	Streptophyta	Magnoliopsida	Cucurbitales	Cucurbitaceae	Cucurbita	Cucurbita moschata
Eukaryota	Streptophyta	Magnoliopsida	Brassicales	Caricaceae	Carica	Carica papaya
Eukaryota	Streptophyta	Magnoliopsida	Cucurbitales	Cucurbitaceae	Cucurbita	Cucurbita pepo
Eukaryota	Streptophyta	Magnoliopsida	Caryophyllales	Chenopodiaceae	Chenopodium	Chenopodium quinoa
Eukaryota	Streptophyta	Magnoliopsida	Brassicales	Brassicaceae	Capsella	Capsella rubella
Eukaryota	Chlorophyta	Chlorophyceae	Chlamydomonadales	Chlamydomonadaceae	Chlamydomonas	Chlamydomonas reinhardtii
Eukaryota	Streptophyta	Magnoliopsida	Brassicales	Brassicaceae	Camelina	Camelina sativa
Eukaryota	Streptophyta	Magnoliopsida	Ericales	Theaceae	Camellia	Camellia sinensis
Eukaryota	Chlorophyta	Trebouxiophyceae	unclassified Trebouxiophyceae	unclassified Trebouxiophyceae family	Coccomyxa	Coccomyxa subellipsoidea
Eukaryota	Streptophyta	Magnoliopsida	Cucurbitales	Cucurbitaceae	Cucumis	Cucumis sativus
Eukaryota	Chlorophyta	Trebouxiophyceae	Chlorellales	Chlorellaceae	Chlorella	Chlorella variabilis
Eukaryota	Streptophyta	Magnoliopsida	Apiales	Apiaceae	Daucus	Daucus carota
Eukaryota	Streptophyta	Magnoliopsida	Asparagales	Orchidaceae	Dendrobium	Dendrobium catenatum
Eukaryota	Chordata	Mammalia	Rodentia	Heteromyidae	Dipodomys	Dipodomys spectabilis
Eukaryota	Streptophyta	Magnoliopsida	Malvales	Malvaceae	Durio	Durio zibethinus
Eukaryota	Streptophyta	Magnoliopsida	Asterales	Asteraceae	Erigeron	Erigeron canadensis
Eukaryota	Streptophyta	Magnoliopsida	Myrtales	Myrtaceae	Eucalyptus	Eucalyptus grandis
Eukaryota	Streptophyta	Magnoliopsida	Arecales	Arecaceae	Elaeis	Elaeis guineensis
Eukaryota	Streptophyta	Magnoliopsida	Brassicales	Brassicaceae	Eutrema	Eutrema salsugineum
Eukaryota	Streptophyta	Magnoliopsida	Rosales	Rosaceae	Fragaria	Fragaria vesca
Eukaryota	Streptophyta	Magnoliopsida	Malvales	Malvaceae	Gossypium	Gossypium arboreum
Eukaryota	Streptophyta	Magnoliopsida	Malvales	Malvaceae	Gossypium	Gossypium hirsutum
Eukaryota	Streptophyta	Magnoliopsida	Fabales	Fabaceae	Glycine	Glycine max
Eukaryota	Streptophyta	Magnoliopsida	Malvales	Malvaceae	Gossypium	Gossypium raimondii
Eukaryota	Streptophyta	Magnoliopsida	Fabales	Fabaceae	Glycine	Glycine soja

Eukaryota	Rhodophyta	Bangiophyceae	Galdieriales	Galdieriaceae	Galdieria	Galdieria sulphuraria
Eukaryota	Streptophyta	Magnoliopsida	Asterales	Asteraceae	Helianthus	Helianthus annuus
Eukaryota	Streptophyta	Magnoliopsida	Malpighiales	Euphorbiaceae	Hevea	Hevea brasiliensis
Eukaryota	Streptophyta	Magnoliopsida	Malvales	Malvaceae	Hibiscus	Hibiscus syriacus
Eukaryota	Streptophyta	Magnoliopsida	Solanales	Convolvulaceae	Ipomoea	Ipomoea nil
Eukaryota	Streptophyta	Magnoliopsida	Solanales	Convolvulaceae	Ipomoea	Ipomoea triloba
Eukaryota	Streptophyta	Magnoliopsida	Malpighiales	Euphorbiaceae	Jatropha	Jatropha curcas
Eukaryota	Streptophyta	Magnoliopsida	Fagales	Juglandaceae	Juglans	Juglans regia
Eukaryota	Streptophyta	Magnoliopsida	Solanales	Solanaceae	Lycium	Lycium barbarum
Eukaryota	Streptophyta	Magnoliopsida	Poales	Poaceae	Lolium	Lolium perenne
Eukaryota	Streptophyta	Magnoliopsida	Asterales	Asteraceae	Lactuca	Lactuca sativa
Eukaryota	Streptophyta	Magnoliopsida	Cucurbitales	Cucurbitaceae	Momordica	Momordica charantia
Eukaryota	Streptophyta	Magnoliopsida	Malpighiales	Euphorbiaceae	Manihot	Manihot esculenta
Eukaryota	Streptophyta	Magnoliopsida	Poales	Poaceae	Miscanthus	Miscanthus floridulus
Eukaryota	Streptophyta	Magnoliopsida	Sapindales	Anacardiaceae	Mangifera	Mangifera indica
Eukaryota	Streptophyta	Magnoliopsida	Proteales	Proteaceae	Macadamia	Macadamia integrifolia
Eukaryota	Streptophyta	Magnoliopsida	Rosales	Moraceae	Morus	Morus notabilis
Eukaryota	Streptophyta	Magnoliopsida	Magnoliales	Magnoliaceae	Magnolia	Magnolia sinica
Eukaryota	Streptophyta	Magnoliopsida	Rosales	Rosaceae	Malus	Malus sylvestris
Eukaryota	Streptophyta	Magnoliopsida	Fabales	Fabaceae	Medicago	Medicago truncatula
Eukaryota	Streptophyta	Magnoliopsida	Solanales	Solanaceae	Nicotiana	Nicotiana attenuata
Eukaryota	Streptophyta	Magnoliopsida	Nymphaeales	Nymphaeaceae	Nymphaea	Nymphaea colorata
Eukaryota	Streptophyta	Magnoliopsida	Proteales	Nelumbonaceae	Nelumbo	Nelumbo nucifera
Eukaryota	Streptophyta	Magnoliopsida	Solanales	Solanaceae	Nicotiana	Nicotiana sylvestris
Eukaryota	Streptophyta	Magnoliopsida	Solanales	Solanaceae	Nicotiana	Nicotiana tabacum
Eukaryota	Streptophyta	Magnoliopsida	Solanales	Solanaceae	Nicotiana	Nicotiana tomentosiformis
Eukaryota	Streptophyta	Magnoliopsida	Poales	Poaceae	Oryza	Oryza brachyantha
Eukaryota	Streptophyta	Magnoliopsida	Lamiales	Oleaceae	Olea	Olea europaea
Eukaryota	Streptophyta	Magnoliopsida	Poales	Poaceae	Oryza	Oryza glaberrima
Eukaryota	Streptophyta	Magnoliopsida	Poales	Poaceae	Oryza	Oryza sativa
Eukaryota	Chlorophyta	Mamiellophyceae	Mamiellales	Bathycoccaceae	Ostreococcus	Ostreococcus tauri
Eukaryota	Streptophyta	Magnoliopsida	Malpighiales	Salicaceae	Populus	Populus alba
Eukaryota	Streptophyta	Magnoliopsida	Fabales	Fabaceae	Prosopis	Prosopis cineraria
Eukaryota	Streptophyta	Magnoliopsida	Arecales	Arecaceae	Phoenix	Phoenix dactylifera
Eukaryota	Streptophyta	Magnoliopsida	Rosales	Rosaceae	Prunus	Prunus dulcis
Eukaryota	Streptophyta	Magnoliopsida	Asparagales	Orchidaceae	Phalaenopsis	Phalaenopsis equestris
Eukaryota	Streptophyta	Magnoliopsida	Malpighiales	Salicaceae	Populus	Populus euphratica
Eukaryota	Streptophyta	Magnoliopsida	Rosales	Rosaceae	Prunus	Prunus mume
Eukaryota	Streptophyta	Magnoliopsida	Malpighiales	Salicaceae	Populus	Populus trichocarpa
Eukaryota	Streptophyta	Magnoliopsida	Rosales	Rosaceae	Prunus	Prunus persica
Eukaryota	Streptophyta	Bryopsida	Funariales	Funariaceae	Physcomitrium	Physcomitrium patens
Eukaryota	Streptophyta	Magnoliopsida	Fabales	Fabaceae	Pisum	Pisum sativum

Eukaryota	Streptophyta	Magnoliopsida	Ranunculales	Papaveraceae	Papaver	Papaver somniferum
Eukaryota	Streptophyta	Magnoliopsida	Poales	Poaceae	Panicum	Panicum virgatum
Eukaryota	Streptophyta	Magnoliopsida	Fabales	Fabaceae	Phaseolus	Phaseolus vulgaris
Eukaryota	Streptophyta	Magnoliopsida	Sapindales	Anacardiaceae	Pistacia	Pistacia vera
Eukaryota	Streptophyta	Magnoliopsida	Fabales	Quillajaceae	Quillaja	Quillaja saponaria
Eukaryota	Streptophyta	Magnoliopsida	Malpighiales	Euphorbiaceae	Ricinus	Ricinus communis
Eukaryota	Streptophyta	Magnoliopsida	Brassicales	Brassicaceae	Raphanus	Raphanus sativus
Eukaryota	Streptophyta	Magnoliopsida	Poales	Poaceae	Sorghum	Sorghum bicolor
Eukaryota	Streptophyta	Magnoliopsida	Solanales	Solanaceae	Solanum	Solanum dulcamara
Eukaryota	Streptophyta	Magnoliopsida	Lamiales	Lamiaceae	Salvia	Salvia hispanica
Eukaryota	Streptophyta	Magnoliopsida	Lamiales	Pedaliaceae	Sesamum	Sesamum indicum
Eukaryota	Streptophyta	Magnoliopsida	Poales	Poaceae	Setaria	Setaria italica
Eukaryota	Streptophyta	Magnoliopsida	Solanales	Solanaceae	Solanum	Solanum lycopersicum
Eukaryota	Streptophyta	Magnoliopsida	Lamiales	Lamiaceae	Salvia	Salvia miltiorrhiza
Eukaryota	Streptophyta	Lycopodiopsida	Selaginellales	Selaginellaceae	Selaginella	Selaginella moellendorffii
Eukaryota	Streptophyta	Magnoliopsida	Caryophyllales	Chenopodiaceae	Spinacia	Spinacia oleracea
Eukaryota	Streptophyta	Magnoliopsida	Solanales	Solanaceae	Solanum	Solanum tuberosum
Eukaryota	Streptophyta	Magnoliopsida	Solanales	Solanaceae	Solanum	Solanum pennellii
Eukaryota	Streptophyta	Magnoliopsida	Lamiales	Lamiaceae	Salvia	Salvia splendens
Eukaryota	Streptophyta	Magnoliopsida	Solanales	Solanaceae	Solanum	Solanum stenotomum
Eukaryota	Streptophyta	Magnoliopsida	Poales	Poaceae	Setaria	Setaria viridis
Eukaryota	Streptophyta	Magnoliopsida	Poales	Poaceae	Triticum	Triticum aestivum
Eukaryota	Streptophyta	Magnoliopsida	Malvales	Malvaceae	Theobroma	Theobroma cacao
Eukaryota	Streptophyta	Magnoliopsida	Brassicales	Cleomaceae	Tarenaya	Tarenaya hassleriana
Eukaryota	Streptophyta	Magnoliopsida	Fabales	Fabaceae	Trifolium	Trifolium pratense
Eukaryota	Bacillariophyta	Coscinodiscophyceae	Thalassiosirales	Thalassiosiraceae	Thalassiosira	Thalassiosira pseudonana
Eukaryota	Streptophyta	Magnoliopsida	Proteales	Proteaceae	Telopea	Telopea speciosissima
Eukaryota	Streptophyta	Magnoliopsida	Poales	Poaceae	Triticum	Triticum urartu
Eukaryota	Streptophyta	Magnoliopsida	Fabales	Fabaceae	Vigna	Vigna angularis
Eukaryota	Streptophyta	Magnoliopsida	Vitales	Vitaceae	Vitis	Vitis riparia
Eukaryota	Streptophyta	Magnoliopsida	Fabales	Fabaceae	Vigna	Vigna umbellata
Eukaryota	Streptophyta	Magnoliopsida	Fabales	Fabaceae	Vigna	Vigna unguiculata
Eukaryota	Streptophyta	Magnoliopsida	Vitales	Vitaceae	Vitis	Vitis vinifera
Eukaryota	Streptophyta	Magnoliopsida	Rosales	Rhamnaceae	Ziziphus	Ziziphus jujuba
Eukaryota	Streptophyta	Magnoliopsida	Poales	Poaceae	Zea	Zea mays
Eukaryota	Streptophyta	Magnoliopsida	Zingiberales	Zingiberaceae	Zingiber	Zingiber officinale

Gene_Organisms: psbC

**Kingdom**	**Phylum**	**Class**	**Order**	**Family**	**Gunus**	**Species**
Bacteria	Cyanobacteriota	Cyanophyceae	Acaryochloridales	Acaryochloridaceae	Acaryochloris	unclassified Acaryochloris species
Bacteria	Cyanobacteriota	Cyanophyceae	Nostocales	Nostocaceae	Anabaena	Anabaena cylindrica
Bacteria	Cyanobacteriota	Cyanophyceae	Nostocales	Nodulariaceae	Anabaenopsis	Anabaenopsis elenkinii
Bacteria	Cyanobacteriota	Cyanophyceae	Nostocales	Aphanizomenonaceae	Aphanizomenon	Aphanizomenon flos-aquae
Bacteria	Cyanobacteriota	Cyanophyceae	Nostocales	Fortieaceae	Aulosira	Aulosira laxa
Bacteria	Cyanobacteriota	Cyanophyceae	Acaryochloridales	Acaryochloridaceae	Acaryochloris	Acaryochloris marina
Bacteria	Cyanobacteriota	Cyanophyceae	Nostocales	Nostocaceae	Nostoc	Nostoc sp.
Bacteria	Cyanobacteriota	Cyanophyceae	Nostocales	Nostocaceae	Anabaena	Anabaena sp.
Bacteria	Cyanobacteriota	Cyanophyceae	Oscillatoriales	Microcoleaceae	Arthrospira	Arthrospira platensis
Bacteria	Cyanobacteriota	Cyanophyceae	Nostocales	Nostocaceae	Trichormus	Trichormus variabilis
Bacteria	Cyanobacteriota	Cyanophyceae	Nostocales	Scytonemataceae	Brasilonema	Brasilonema octagenarum
Bacteria	Cyanobacteriota	Cyanophyceae	Nostocales	Scytonemataceae	Brasilonema	Brasilonema sennae
Bacteria	Cyanobacteriota	Cyanophyceae	Nostocales	Calotrichaceae	Calothrix	Calothrix sp.
Bacteria	Cyanobacteriota	Cyanophyceae	Chroococcales	Geminocystaceae	Cyanobacterium	Cyanobacterium aponinum
Bacteria	Cyanobacteriota	Cyanophyceae	Nostocales	Aphanizomenonaceae	Cylindrospermopsis	Cylindrospermopsis curvispora
Bacteria	Cyanobacteriota	Cyanophyceae	Gomontiellales	Gomontiellaceae	Crinalium	Crinalium epipsammum
Bacteria	Cyanobacteriota	Cyanophyceae	Synechococcales	Prochlorococcaceae	Cyanobium	Cyanobium gracile
Bacteria	Cyanobacteriota	Cyanophyceae	Chroococcales	Chroococcaceae	Chondrocystis	unclassified Chondrocystis species
Bacteria	Cyanobacteriota	Cyanophyceae	Gomontiellales	Chamaesiphonaceae	Chamaesiphon	Chamaesiphon minutus
Bacteria	Cyanobacteriota	Cyanophyceae	Nostocales	Aphanizomenonaceae	Cylindrospermopsis	Cylindrospermopsis raciborskii
Bacteria	Cyanobacteriota	Cyanophyceae	Nostocales	Nostocaceae	Cylindrospermum	Cylindrospermum stagnale
Bacteria	Cyanobacteriota	Cyanophyceae	Chroococcales	Geminocystaceae	Cyanobacterium	Cyanobacterium stanieri
Bacteria	Cyanobacteriota	Cyanophyceae	Chroococcidiopsidales	Chroococcidiopsidaceae	Chroococcidiopsis	Chroococcidiopsis thermalis
Bacteria	Cyanobacteriota	Cyanophyceae	Chroococcales	Aphanothecaceae	Crocosphaera	Crocosphaera watsonii
Bacteria	Cyanobacteriota	Cyanophyceae	Synechococcales	Synechococcaceae	Synechococcus	Synechococcus sp.
Bacteria	Cyanobacteriota	Cyanophyceae	Chroococcales	Aphanothecaceae	Gloeothece	Gloeothece citriformis
Bacteria	Cyanobacteriota	Cyanophyceae	Chroococcales	Aphanothecaceae	Rippkaea	Rippkaea orientalis
Bacteria	Cyanobacteriota	Cyanophyceae	Synechococcales	Prochlorococcaceae	Cyanobium	Cyanobium sp.
Bacteria	Cyanobacteriota	Cyanophyceae	Chroococcales	Aphanothecaceae	Gloeothece	Gloeothece verrucosa
Bacteria	Cyanobacteriota	Cyanophyceae	Chroococcales	Geminocystaceae	Cyanobacterium	Cyanobacterium sp.
Bacteria	Cyanobacteriota	Cyanophyceae	Gomontiellales	Cyanothecaceae	Cyanothece	Cyanothece sp.
Bacteria	Cyanobacteriota	Cyanophyceae	Chroococcales	Aphanothecaceae	Crocosphaera	Crocosphaera subtropica
Bacteria	Cyanobacteriota	Cyanophyceae	Nostocales	Aphanizomenonaceae	Dolichospermum	Dolichospermum compactum
Bacteria	Cyanobacteriota	Cyanophyceae	Nostocales	Aphanizomenonaceae	Dolichospermum	Dolichospermum flos-aquae
Bacteria	Cyanobacteriota	Cyanophyceae	Nostocales	Aphanizomenonaceae	Dolichospermum	Dolichospermum heterosporum
Bacteria	Cyanobacteriota	Cyanophyceae	Nostocales	Aphanizomenonaceae	Dolichospermum	Dolichospermum sp.
Bacteria	Cyanobacteriota	Cyanophyceae	Synechococcales	Synechococcaceae	Dactylococcopsis	Dactylococcopsis salina
Bacteria	Cyanobacteriota	Cyanophyceae	Chroococcales	Halothecacae	Euhalothece	Euhalothece natronophila
Bacteria	Cyanobacteriota	Cyanophyceae	Nostocales	Hapalosiphonaceae	Fischerella	Fischerella sp.
Bacteria	Cyanobacteriota	Cyanophyceae	Chroococcales	Chroococcaceae	Gloeocapsopsis	Gloeocapsopsis dulcis

Bacteria	Cyanobacteriota	Cyanophyceae	Chroococcales	Geminocystaceae	Geminocystis	Geminocystis sp.
Bacteria	Cyanobacteriota	Cyanophyceae	Geitlerinematales	Geitlerinemataceae	Geitlerinema	Geitlerinema sp.
Bacteria	Cyanobacteriota	Cyanophyceae	Gloeobacterales	Gloeobacteraceae	Gloeobacter	Gloeobacter kilaueensis
Bacteria	Cyanobacteriota	Cyanophyceae	Chroococcales	Chroococcaceae	Gloeocapsa	unclassified Gloeocapsa species
Bacteria	Cyanobacteriota	Cyanophyceae	Gloeomargaritales	Gloeomargaritaceae	Gloeomargarita	Gloeomargarita lithophora
Bacteria	Cyanobacteriota	Cyanophyceae	Gloeobacterales	Gloeobacteraceae	Gloeobacter	Gloeobacter morelensis
Bacteria	Cyanobacteriota	Cyanophyceae	Gloeobacterales	Gloeobacteraceae	Gloeobacter	Gloeobacter violaceus
Bacteria	Cyanobacteriota	Cyanophyceae	Chroococcales	Halothecacae	Halothece	Halothece sp.
Bacteria	Cyanobacteriota	Cyanophyceae	Nostocales	Nodulariaceae	Halotia	Halotia branconii
Bacteria	Cyanobacteriota	Cyanophyceae	Nodosilineales	Nodosilineaceae	Halomicronema	Halomicronema hongdechloris
Bacteria	Cyanobacteriota	Cyanophyceae	Leptolyngbyales	Leptolyngbyaceae	Kovacikia	Kovacikia minuta
Bacteria	Cyanobacteriota	Cyanophyceae	Leptolyngbyales	Leptolyngbyaceae	Leptolyngbya	Leptolyngbya boryana
Bacteria	Cyanobacteriota	Cyanophyceae	Leptolyngbyales	Leptolyngbyaceae	Leptolyngbya	Leptolyngbya sp.
Bacteria	Cyanobacteriota	Cyanophyceae	Oscillatoriales	Sirenicapillariaceae	Limnospira	Limnospira fusiformis
Bacteria	Cyanobacteriota	Cyanophyceae	Oscillatoriales	Sirenicapillariaceae	Limnospira	Limnospira indica
Bacteria	Cyanobacteriota	Cyanophyceae	Leptolyngbyales	Leptolyngbyaceae	Leptodesmis	Leptodesmis sichuanensis
Bacteria	Cyanobacteriota	Cyanophyceae	Leptolyngbyales	Leptolyngbyaceae	Leptothermofonsia	Leptothermofonsia sichuanensis
Bacteria	Cyanobacteriota	Cyanophyceae	Chroococcales	Microcystaceae	Microcystis	Microcystis aeruginosa
Bacteria	Cyanobacteriota	Cyanophyceae	Nostocales	Rivulariaceae	Microchaete	Microchaete diplosiphon
Bacteria	Cyanobacteriota	Cyanophyceae	Coleofasciculales	Coleofasciculaceae	Allocoleopsis	Allocoleopsis franciscana
Bacteria	Cyanobacteriota	Cyanophyceae	Chroococcales	Microcystaceae	Microcystis	Microcystis sp.
Bacteria	Cyanobacteriota	Cyanophyceae	Chroococcales	Microcystaceae	Microcystis	Microcystis panniformis
Bacteria	Cyanobacteriota	Cyanophyceae	Oscillatoriales	Oscillatoriaceae	Moorena	Moorena producens
Bacteria	Cyanobacteriota	Cyanophyceae	Oscillatoriales	Microcoleaceae	Microcoleus	Microcoleus vaginatus
Bacteria	Cyanobacteriota	Cyanophyceae	Chroococcales	Microcystaceae	Microcystis	Microcystis viridis
Bacteria	Cyanobacteriota	Cyanophyceae	Nostocales	unclassified Nostocales family	unclassified Nostocales genus	Nostocales cyanobacterium
Bacteria	Cyanobacteriota	Cyanophyceae	Nostocales	Nostocaceae	Nostoc	Nostoc carneum
Bacteria	Cyanobacteriota	Cyanophyceae	Nostocales	Nostocaceae	Nostoc	Nostoc edaphicum
Bacteria	Cyanobacteriota	Cyanophyceae	Nostocales	Nostocaceae	Nostoc	Nostoc flagelliforme
Bacteria	Cyanobacteriota	Cyanophyceae	Nostocales	Nostocaceae	Nostoc	Nostoc linckia
Bacteria	Cyanobacteriota	Cyanophyceae	Nostocales	Nostocaceae	Nostoc	Nostoc punctiforme
Bacteria	Cyanobacteriota	Cyanophyceae	Nostocales	Nostocaceae	Nostoc	Nostoc piscinale
Bacteria	Cyanobacteriota	Cyanophyceae	Nostocales	Nostocaceae	Nostoc	Nostoc sphaeroides
Bacteria	Cyanobacteriota	Cyanophyceae	Nostocales	Nodulariaceae	Nodularia	Nodularia spumigena
Bacteria	Cyanobacteriota	Cyanophyceae	Nostocales	Nodulariaceae	Nodularia	Nodularia sphaerocarpa
Bacteria	Cyanobacteriota	Cyanophyceae	Oscillatoriales	Oscillatoriaceae	Oscillatoria	Oscillatoria acuminata
Bacteria	Cyanobacteriota	Cyanophyceae	Nostocales	Aphanizomenonaceae	Okeanomitos	Okeanomitos corallinicola
Bacteria	Cyanobacteriota	Cyanophyceae	Oscillatoriales	Oscillatoriaceae	Oscillatoria	Oscillatoria nigro-viridis
Bacteria	Cyanobacteriota	Cyanophyceae	Oscillatoriales	Oscillatoriaceae	Oxynema	Oxynema aestuarii
Bacteria	Cyanobacteriota	Cyanophyceae	Oscillatoriales	Microcoleaceae	Planktothrix	Planktothrix agardhii
Bacteria	Cyanobacteriota	Cyanophyceae	Pseudanabaenales	Pseudanabaenaceae	Pseudanabaena	Pseudanabaena galeata

Bacteria	Cyanobacteriota	Cyanophyceae	Oscillatoriales	Oscillatoriaceae	Phormidium	Phormidium sp.
Bacteria	Cyanobacteriota	Cyanophyceae	Chroococcales	Geminocystaceae	Picosynechococcus	unclassified Picosynechococcus species
Bacteria	Cyanobacteriota	Cyanophyceae	Pleurocapsales	Hyellaceae	Pleurocapsa	Pleurocapsa sp.
Bacteria	Cyanobacteriota	Cyanophyceae	Synechococcales	Prochlorococcaceae	Prochlorococcus	Prochlorococcus marinus
Bacteria	Cyanobacteriota	Cyanophyceae	Oscillatoriales	Microcoleaceae	Planktothrix	Planktothrix pseudagardhii
Bacteria	Cyanobacteriota	Cyanophyceae	Synechococcales	Prochlorococcaceae	Prochlorococcus	Prochlorococcus sp.
Bacteria	Cyanobacteriota	Cyanophyceae	Oscillatoriales	Microcoleaceae	Planktothrix	Planktothrix rubescens
Bacteria	Cyanobacteriota	Cyanophyceae	Pseudanabaenales	Pseudanabaenaceae	Pseudanabaena	Pseudanabaena sp.
Bacteria	Cyanobacteriota	Cyanophyceae	Oscillatoriales	Oscillatoriaceae	Phormidium	Phormidium yuhuli
Bacteria	Cyanobacteriota	Cyanophyceae	Nostocales	Aphanizomenonaceae	Raphidiopsis	Raphidiopsis curvata
Bacteria	Cyanobacteriota	Cyanophyceae	Nostocales	Nostocaceae	Richelia	Richelia sinica
Bacteria	Cyanobacteriota	Cyanophyceae	Pleurocapsales	Dermocarpellaceae	Stanieria	Stanieria cyanosphaera
Bacteria	Cyanobacteriota	Cyanophyceae	Nostocales	Scytonemataceae	Scytonema	Scytonema sp.
Bacteria	Cyanobacteriota	Cyanophyceae	Nostocales	Aphanizomenonaceae	Sphaerospermopsis	Sphaerospermopsis kisseleviana
Bacteria	Cyanobacteriota	Cyanophyceae	Acaryochloridales	Thermosynechococcaceae	Parathermosynechococcu	Parathermosynechococcus lividus
Bacteria	Cyanobacteriota	Cyanophyceae	Pleurocapsales	Dermocarpellaceae	Stanieria	Stanieria sp.
Bacteria	Cyanobacteriota	Cyanophyceae	Nostocales	Aphanizomenonaceae	Sphaerospermopsis	Sphaerospermopsis torques-reginae
Bacteria	Cyanobacteriota	Cyanophyceae	Synechococcales	Synechococcaceae	Synechococcus	Synechococcus elongatus
Bacteria	Cyanobacteriota	Cyanophyceae	Synechococcales	Merismopediaceae	Synechocystis	Synechocystis sp.
Bacteria	Cyanobacteriota	Cyanophyceae	Synechococcales	Prochlorococcaceae	Parasynechococcus	Parasynechococcus marenigrum
Bacteria	Cyanobacteriota	Cyanophyceae	Acaryochloridales	Thermosynechococcaceae	Thermosynechococcus	Thermosynechococcus vestitus
Bacteria	Cyanobacteriota	Cyanophyceae	Oscillatoriales	Microcoleaceae	Trichodesmium	Trichodesmium erythraeum
Bacteria	Cyanobacteriota	Cyanophyceae	Acaryochloridales	Thermosynechococcaceae	Thermosynechococcus	Thermosynechococcus sp.
Bacteria	Cyanobacteriota	Cyanophyceae	Oculatellales	Oculatellaceae	Thermoleptolyngbya	Thermoleptolyngbya sichuanensis
Bacteria	Cyanobacteriota	Cyanophyceae	Oculatellales	Oculatellaceae	Thermoleptolyngbya	Thermoleptolyngbya oregonensis
Bacteria	Cyanobacteriota	Cyanophyceae	Nostocales	Tolypothrichaceae	Tolypothrix	Tolypothrix sp.
Bacteria	Cyanobacteriota	Cyanophyceae	Oculatellales	Oculatellaceae	Thermocoleostomius	Thermocoleostomius sinensis
Bacteria	Cyanobacteriota	Cyanophyceae	Acaryochloridales	Thermosynechococcaceae	Thermosynechococcus	Thermosynechococcus sichuanensis
Bacteria	Cyanobacteriota	Cyanophyceae	Leptolyngbyales	Trichocoleusaceae	Trichothermofontia	Trichothermofontia sichuanensis
Bacteria	Cyanobacteriota	Cyanophyceae	Nostocales	Tolypothrichaceae	Tolypothrix	Tolypothrix tenuis
Bacteria	Cyanobacteriota	Cyanophyceae	Thermostichales	Thermostichaceae	Thermostichus	Thermostichus vulcanus
Bacteria	Cyanobacteriota	Cyanophyceae	Synechococcales	Coelosphaeriaceae	Woronichinia	Woronichinia naegeliana
Eukaryota	unclassified Eukaryota	Pelagophyceae	Pelagomonadales	Pelagomonadaceae	Aureococcus	Aureococcus anophagefferens
Eukaryota	Streptophyta	Magnoliopsida	Fabales	Fabaceae	Arachis	Arachis duranensis
Eukaryota	Streptophyta	Magnoliopsida	Ericales	Actinidiaceae	Actinidia	Actinidia eriantha
Eukaryota	Streptophyta	Magnoliopsida	Fabales	Fabaceae	Arachis	Arachis hypogaea
Eukaryota	Streptophyta	Magnoliopsida	Fabales	Fabaceae	Arachis	Arachis ipaensis
Eukaryota	Streptophyta	Magnoliopsida	Brassicales	Brassicaceae	Arabidopsis	Arabidopsis lyrata
Eukaryota	Streptophyta	Magnoliopsida	Asparagales	Asparagaceae	Asparagus	Asparagus officinalis
Eukaryota	Streptophyta	Magnoliopsida	Lamiales	Acanthaceae	Andrographis	Andrographis paniculata
Eukaryota	Streptophyta	Magnoliopsida	Fabales	Fabaceae	Abrus	Abrus precatorius

Eukaryota	Chlorophyta	Trebouxiophyceae	Chlorellales	Chlorellaceae	Auxenochlorella	Auxenochlorella protothecoides
Eukaryota	Streptophyta	Magnoliopsida	Brassicales	Brassicaceae	Arabidopsis	Arabidopsis thaliana
Eukaryota	Streptophyta	Magnoliopsida	Amborellales	Amborellaceae	Amborella	Amborella trichopoda
Eukaryota	Streptophyta	Magnoliopsida	Caryophyllales	Amaranthaceae	Amaranthus	Amaranthus tricolor
Eukaryota	Streptophyta	Magnoliopsida	Poales	Poaceae	Brachypodium	Brachypodium distachyon
Eukaryota	Streptophyta	Magnoliopsida	Cucurbitales	Cucurbitaceae	Benincasa	Benincasa hispida
Eukaryota	Streptophyta	Magnoliopsida	Brassicales	Brassicaceae	Brassica	Brassica napus
Eukaryota	Streptophyta	Magnoliopsida	Brassicales	Brassicaceae	Brassica	Brassica oleracea
Eukaryota	Chlorophyta	Mamiellophyceae	Mamiellales	Bathycoccaceae	Bathycoccus	Bathycoccus prasinos
Eukaryota	Streptophyta	Magnoliopsida	Brassicales	Brassicaceae	Brassica	Brassica rapa
Eukaryota	Streptophyta	Magnoliopsida	Fabales	Fabaceae	Cicer	Cicer arietinum
Eukaryota	Streptophyta	Magnoliopsida	Solanales	Solanaceae	Capsicum	Capsicum annuum
Eukaryota	Streptophyta	Magnoliopsida	Fagales	Betulaceae	Corylus	Corylus avellana
Eukaryota	Streptophyta	Magnoliopsida	Fabales	Fabaceae	Cajanus	Cajanus cajan
Eukaryota	Rhodophyta	Florideophyceae	Gigartinales	Gigartinaceae	Chondrus	Chondrus crispus
Eukaryota	Streptophyta	Magnoliopsida	Sapindales	Rutaceae	Citrus	Citrus sinensis
Eukaryota	Streptophyta	Magnoliopsida	Cucurbitales	Cucurbitaceae	Cucurbita	Cucurbita maxima
Eukaryota	Rhodophyta	Bangiophyceae	Cyanidiales	Cyanidiaceae	Cyanidioschyzon	Cyanidioschyzon merolae
Eukaryota	Streptophyta	Magnoliopsida	Cucurbitales	Cucurbitaceae	Cucurbita	Cucurbita moschata
Eukaryota	Streptophyta	Magnoliopsida	Brassicales	Caricaceae	Carica	Carica papaya
Eukaryota	Streptophyta	Magnoliopsida	Caryophyllales	Chenopodiaceae	Chenopodium	Chenopodium quinoa
Eukaryota	Streptophyta	Magnoliopsida	Brassicales	Brassicaceae	Capsella	Capsella rubella
Eukaryota	Chlorophyta	Chlorophyceae	Chlamydomonadales	Chlamydomonadaceae	Chlamydomonas	Chlamydomonas reinhardtii
Eukaryota	Streptophyta	Magnoliopsida	Brassicales	Brassicaceae	Camelina	Camelina sativa
Eukaryota	Streptophyta	Magnoliopsida	Ericales	Theaceae	Camellia	Camellia sinensis
Eukaryota	Chlorophyta	Trebouxiophyceae	unclassified Trebouxiophyceae	unclassified Trebouxiophyceae family	Coccomyxa	Coccomyxa subellipsoidea
Eukaryota	Streptophyta	Magnoliopsida	Cucurbitales	Cucurbitaceae	Cucumis	Cucumis sativus
Eukaryota	Chlorophyta	Trebouxiophyceae	Chlorellales	Chlorellaceae	Chlorella	Chlorella variabilis
Eukaryota	Streptophyta	Magnoliopsida	Apiales	Apiaceae	Daucus	Daucus carota
Eukaryota	Streptophyta	Magnoliopsida	Asparagales	Orchidaceae	Dendrobium	Dendrobium catenatum
Eukaryota	Chordata	Mammalia	Rodentia	Heteromyidae	Dipodomys	Dipodomys spectabilis
Eukaryota	Streptophyta	Magnoliopsida	Malvales	Malvaceae	Durio	Durio zibethinus
Eukaryota	Streptophyta	Magnoliopsida	Asterales	Asteraceae	Erigeron	Erigeron canadensis
Eukaryota	Streptophyta	Magnoliopsida	Myrtales	Myrtaceae	Eucalyptus	Eucalyptus grandis
Eukaryota	Streptophyta	Magnoliopsida	Arecales	Arecaceae	Elaeis	Elaeis guineensis
Eukaryota	Streptophyta	Magnoliopsida	Brassicales	Brassicaceae	Eutrema	Eutrema salsugineum
Eukaryota	Streptophyta	Magnoliopsida	Rosales	Rosaceae	Fragaria	Fragaria vesca
Eukaryota	Streptophyta	Magnoliopsida	Malvales	Malvaceae	Gossypium	Gossypium arboreum
Eukaryota	Streptophyta	Magnoliopsida	Malvales	Malvaceae	Gossypium	Gossypium hirsutum
Eukaryota	Streptophyta	Magnoliopsida	Fabales	Fabaceae	Glycine	Glycine max
Eukaryota	Streptophyta	Magnoliopsida	Malvales	Malvaceae	Gossypium	Gossypium raimondii

Eukaryota	Streptophyta	Magnoliopsida	Fabales	Fabaceae	Glycine	Glycine soja
Eukaryota	Rhodophyta	Bangiophyceae	Galdieriales	Galdieriaceae	Galdieria	Galdieria sulphuraria
Eukaryota	Streptophyta	Magnoliopsida	Asterales	Asteraceae	Helianthus	Helianthus annuus
Eukaryota	Streptophyta	Magnoliopsida	Malpighiales	Euphorbiaceae	Hevea	Hevea brasiliensis
Eukaryota	Streptophyta	Magnoliopsida	Malvales	Malvaceae	Hibiscus	Hibiscus syriacus
Eukaryota	Streptophyta	Magnoliopsida	Solanales	Convolvulaceae	Ipomoea	Ipomoea nil
Eukaryota	Streptophyta	Magnoliopsida	Solanales	Convolvulaceae	Ipomoea	Ipomoea triloba
Eukaryota	Streptophyta	Magnoliopsida	Malpighiales	Euphorbiaceae	Jatropha	Jatropha curcas
Eukaryota	Streptophyta	Magnoliopsida	Fagales	Juglandaceae	Juglans	Juglans regia
Eukaryota	Streptophyta	Magnoliopsida	Solanales	Solanaceae	Lycium	Lycium barbarum
Eukaryota	Streptophyta	Magnoliopsida	Poales	Poaceae	Lolium	Lolium perenne
Eukaryota	Streptophyta	Magnoliopsida	Asterales	Asteraceae	Lactuca	Lactuca sativa
Eukaryota	Streptophyta	Magnoliopsida	Cucurbitales	Cucurbitaceae	Momordica	Momordica charantia
Eukaryota	Streptophyta	Magnoliopsida	Malpighiales	Euphorbiaceae	Mercurialis	Mercurialis annua
Eukaryota	Streptophyta	Magnoliopsida	Malpighiales	Euphorbiaceae	Manihot	Manihot esculenta
Eukaryota	Streptophyta	Magnoliopsida	Poales	Poaceae	Miscanthus	Miscanthus floridulus
Eukaryota	Streptophyta	Magnoliopsida	Sapindales	Anacardiaceae	Mangifera	Mangifera indica
Eukaryota	Streptophyta	Magnoliopsida	Proteales	Proteaceae	Macadamia	Macadamia integrifolia
Eukaryota	Streptophyta	Magnoliopsida	Rosales	Moraceae	Morus	Morus notabilis
Eukaryota	Streptophyta	Magnoliopsida	Santalales	Ximeniaceae	Malania	Malania oleifera
Eukaryota	Streptophyta	Magnoliopsida	Magnoliales	Magnoliaceae	Magnolia	Magnolia sinica
Eukaryota	Streptophyta	Magnoliopsida	Rosales	Rosaceae	Malus	Malus sylvestris
Eukaryota	Streptophyta	Magnoliopsida	Fabales	Fabaceae	Medicago	Medicago truncatula
Eukaryota	Streptophyta	Magnoliopsida	Solanales	Solanaceae	Nicotiana	Nicotiana attenuata
Eukaryota	Streptophyta	Magnoliopsida	Nymphaeales	Nymphaeaceae	Nymphaea	Nymphaea colorata
Eukaryota	Streptophyta	Magnoliopsida	Proteales	Nelumbonaceae	Nelumbo	Nelumbo nucifera
Eukaryota	Streptophyta	Magnoliopsida	Solanales	Solanaceae	Nicotiana	Nicotiana sylvestris
Eukaryota	Streptophyta	Magnoliopsida	Solanales	Solanaceae	Nicotiana	Nicotiana tabacum
Eukaryota	Streptophyta	Magnoliopsida	Solanales	Solanaceae	Nicotiana	Nicotiana tomentosiformis
Eukaryota	Streptophyta	Magnoliopsida	Poales	Poaceae	Oryza	Oryza brachyantha
Eukaryota	Streptophyta	Magnoliopsida	Poales	Poaceae	Oryza	Oryza glaberrima
Eukaryota	Streptophyta	Magnoliopsida	Poales	Poaceae	Oryza	Oryza sativa
Eukaryota	Chlorophyta	Mamiellophyceae	Mamiellales	Bathycoccaceae	Ostreococcus	Ostreococcus tauri
Eukaryota	Streptophyta	Magnoliopsida	Malpighiales	Salicaceae	Populus	Populus alba
Eukaryota	Streptophyta	Magnoliopsida	Fabales	Fabaceae	Prosopis	Prosopis cineraria
Eukaryota	Streptophyta	Magnoliopsida	Arecales	Arecaceae	Phoenix	Phoenix dactylifera
Eukaryota	Streptophyta	Magnoliopsida	Rosales	Rosaceae	Prunus	Prunus dulcis
Eukaryota	Streptophyta	Magnoliopsida	Asparagales	Orchidaceae	Phalaenopsis	Phalaenopsis equestris
Eukaryota	Streptophyta	Magnoliopsida	Malpighiales	Salicaceae	Populus	Populus euphratica
Eukaryota	Streptophyta	Magnoliopsida	Rosales	Rosaceae	Prunus	Prunus mume
Eukaryota	Streptophyta	Magnoliopsida	Malpighiales	Salicaceae	Populus	Populus trichocarpa
Eukaryota	Streptophyta	Magnoliopsida	Rosales	Rosaceae	Prunus	Prunus persica

Eukaryota	Streptophyta	Bryopsida	Funariales	Funariaceae	Physcomitrium	Physcomitrium patens
Eukaryota	Streptophyta	Magnoliopsida	Fabales	Fabaceae	Pisum	Pisum sativum
Eukaryota	Streptophyta	Magnoliopsida	Ranunculales	Papaveraceae	Papaver	Papaver somniferum
Eukaryota	Streptophyta	Magnoliopsida	Poales	Poaceae	Panicum	Panicum virgatum
Eukaryota	Streptophyta	Magnoliopsida	Fabales	Fabaceae	Phaseolus	Phaseolus vulgaris
Eukaryota	Streptophyta	Magnoliopsida	Fabales	Quillajaceae	Quillaja	Quillaja saponaria
Eukaryota	Streptophyta	Magnoliopsida	Malpighiales	Euphorbiaceae	Ricinus	Ricinus communis
Eukaryota	Streptophyta	Magnoliopsida	Brassicales	Brassicaceae	Raphanus	Raphanus sativus
Eukaryota	Streptophyta	Magnoliopsida	Poales	Poaceae	Sorghum	Sorghum bicolor
Eukaryota	Streptophyta	Magnoliopsida	Solanales	Solanaceae	Solanum	Solanum dulcamara
Eukaryota	Streptophyta	Magnoliopsida	Lamiales	Lamiaceae	Salvia	Salvia hispanica
Eukaryota	Streptophyta	Magnoliopsida	Lamiales	Pedaliaceae	Sesamum	Sesamum indicum
Eukaryota	Streptophyta	Magnoliopsida	Poales	Poaceae	Setaria	Setaria italica
Eukaryota	Streptophyta	Magnoliopsida	Solanales	Solanaceae	Solanum	Solanum lycopersicum
Eukaryota	Streptophyta	Magnoliopsida	Lamiales	Lamiaceae	Salvia	Salvia miltiorrhiza
Eukaryota	Streptophyta	Lycopodiopsida	Selaginellales	Selaginellaceae	Selaginella	Selaginella moellendorffii
Eukaryota	Streptophyta	Magnoliopsida	Caryophyllales	Chenopodiaceae	Spinacia	Spinacia oleracea
Eukaryota	Streptophyta	Magnoliopsida	Solanales	Solanaceae	Solanum	Solanum tuberosum
Eukaryota	Streptophyta	Magnoliopsida	Solanales	Solanaceae	Solanum	Solanum pennellii
Eukaryota	Streptophyta	Magnoliopsida	Lamiales	Lamiaceae	Salvia	Salvia splendens
Eukaryota	Streptophyta	Magnoliopsida	Solanales	Solanaceae	Solanum	Solanum stenotomum
Eukaryota	Streptophyta	Magnoliopsida	Poales	Poaceae	Setaria	Setaria viridis
Eukaryota	Streptophyta	Magnoliopsida	Poales	Poaceae	Triticum	Triticum aestivum
Eukaryota	Streptophyta	Magnoliopsida	Malvales	Malvaceae	Theobroma	Theobroma cacao
Eukaryota	Streptophyta	Magnoliopsida	Poales	Poaceae	Triticum	Triticum dicoccoides
Eukaryota	Streptophyta	Magnoliopsida	Brassicales	Cleomaceae	Tarenaya	Tarenaya hassleriana
Eukaryota	Streptophyta	Magnoliopsida	Fabales	Fabaceae	Trifolium	Trifolium pratense
Eukaryota	Bacillariophyta	Coscinodiscophyceae	Thalassiosirales	Thalassiosiraceae	Thalassiosira	Thalassiosira pseudonana
Eukaryota	Streptophyta	Magnoliopsida	Proteales	Proteaceae	Telopea	Telopea speciosissima
Eukaryota	Streptophyta	Magnoliopsida	Poales	Poaceae	Triticum	Triticum urartu
Eukaryota	Streptophyta	Magnoliopsida	Fabales	Fabaceae	Vigna	Vigna angularis
Eukaryota	Streptophyta	Magnoliopsida	Vitales	Vitaceae	Vitis	Vitis riparia
Eukaryota	Streptophyta	Magnoliopsida	Fabales	Fabaceae	Vigna	Vigna umbellata
Eukaryota	Streptophyta	Magnoliopsida	Fabales	Fabaceae	Vigna	Vigna unguiculata
Eukaryota	Streptophyta	Magnoliopsida	Vitales	Vitaceae	Vitis	Vitis vinifera
Eukaryota	Streptophyta	Magnoliopsida	Fabales	Fabaceae	Vicia	Vicia villosa
Eukaryota	Streptophyta	Magnoliopsida	Rosales	Rhamnaceae	Ziziphus	Ziziphus jujuba
Eukaryota	Streptophyta	Magnoliopsida	Poales	Poaceae	Zea	Zea mays
Eukaryota	Streptophyta	Magnoliopsida	Zingiberales	Zingiberaceae	Zingiber	Zingiber officinale
